# Access to optically active tetrafluoroethylenated amines based on [1,3]-proton shift reaction

**DOI:** 10.3762/bjoc.20.233

**Published:** 2024-11-01

**Authors:** Yuta Kabumoto, Eiichiro Yoshimoto, Bing Xiaohuan, Masato Morita, Motohiro Yasui, Shigeyuki Yamada, Tsutomu Konno

**Affiliations:** 1 Faculty of Molecular Chemistry and Engineering, Kyoto Institute of Technology, Matsugasaki, Sakyo-ku, Kyoto 606-8585, Japan; 2 Department of Materials Science and Engineering, Graduate School of Science and Engineering, Ibaraki University, 4-12-1 Nakanarusawa, Hitachi, Ibaraki 316-8511, Japan

**Keywords:** amine, chirality transfer, [1,3]-proton shift reaction, tetrafluoroethylene fragment

## Abstract

Treatment of various (*R*)-*N*-(2,2,3,3-tetrafluoropent-4-en-1-ylidene)-1-phenylethylamine derivatives with 2.4 equiv of DBU in toluene at room temperature to 50 °C for 24 h led to a smooth [1,3]-proton shift reaction with a high chirality transfer, affording the corresponding rearranged products in acceptable yields. Without purification, these products were subjected to acid hydrolysis and the subsequent *N*-Cbz protection, providing the optically active tetrafluoroethylenated amides in moderate three-step yields.

## Introduction

A fluorine atom has quite peculiar chemical and physical properties compared to others, and hence changes in molecular properties resulting from the introduction of fluorine atom(s) into organic molecules are also significantly unique, and often extremely noticeable even when the number of the atom introduced is small [1–3]. By skillfully utilizing such characteristics, fluorine-containing organic molecules have established themselves as indispensable compounds in various frontlines, such as medicinal, agrochemical, and material fields [4–7].

**Figure 1:**
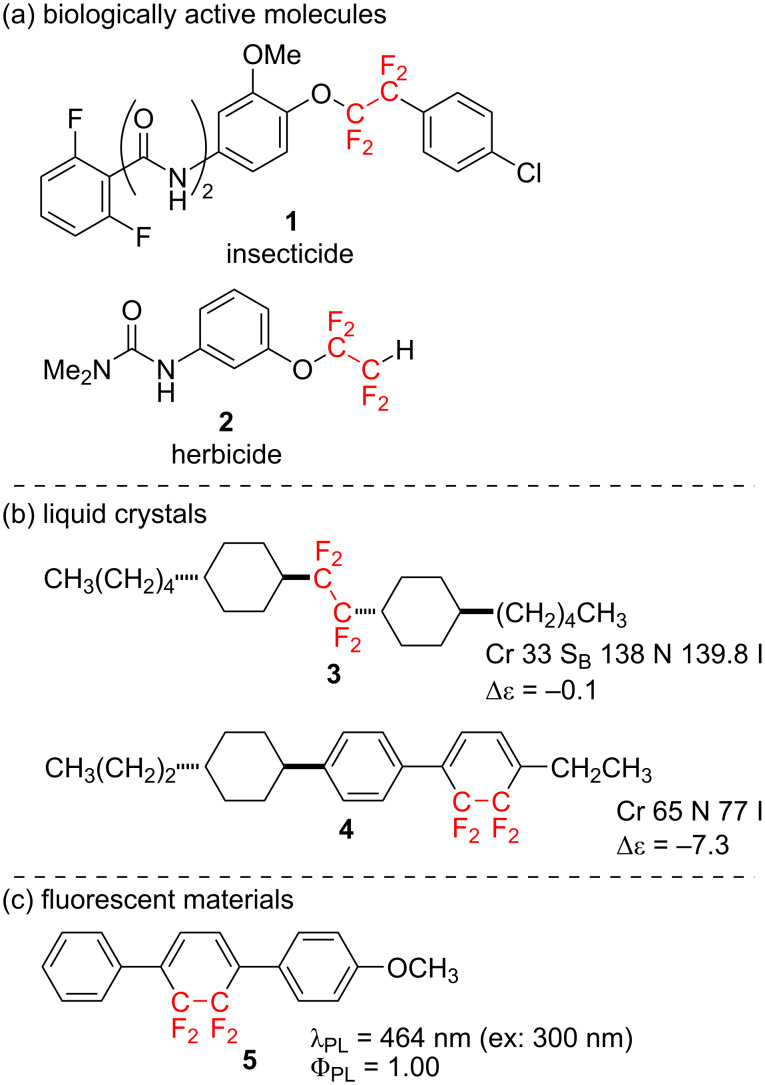
Various applications of tetrafluoroethylenated molecules.

In particular, tetrafluoroethylenated compounds possessing two fluorine atoms on each of two adjacent carbons, have been attracting an enormous attention these days. This stems from the fact that substances with a tetrafluoroethylene fragment exhibit significantly different molecular properties compared to monofluorinated, difluorinated, or trifluoromethylated molecules [8–9]. Therefore, more and more tetrafluoroethylenated molecules having a variety of applications, such as bioactive substances (Figure 1a, **1**, **2**) [10–12], liquid crystals (Figure 1b, **3**, **4**) [13–17], fluorescence materials (Figure 1c, **5**) [18–19], and so on, have been developed in recent years.

**Scheme 1:**
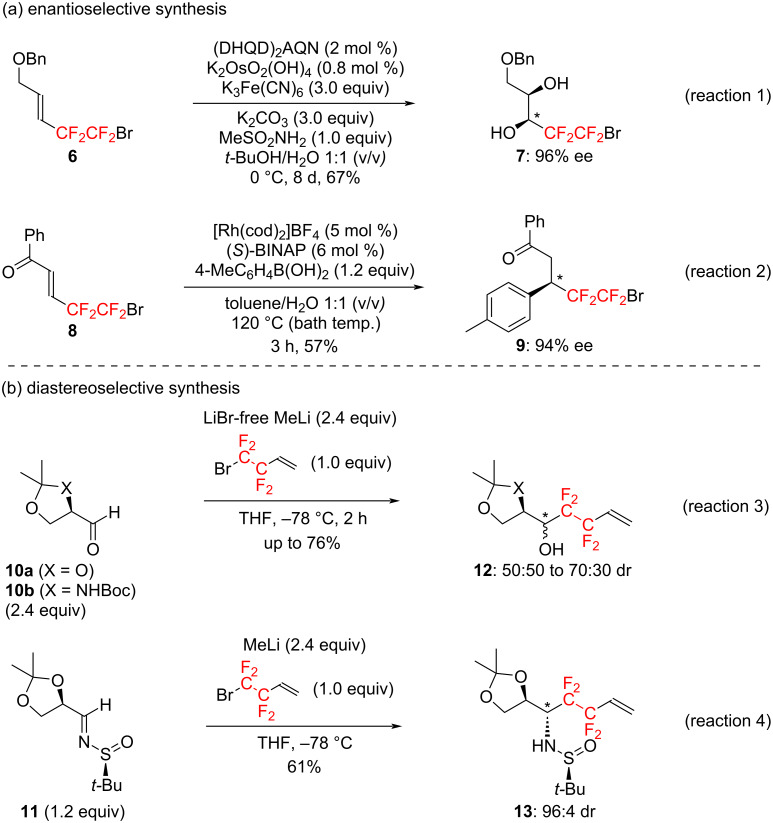
Precedent synthetic approaches to optically active compounds possessing a tetrafluoroethylene group on an asymmetric carbon center.

In sharp contrast to the major development of such non-chiral tetrafluoroethylenated compounds, there have been only few reports on the preparation of chiral molecules possessing a tetrafluoroethylene unit on an asymmetric carbon center in a high optical purity, and to the best of our knowledge, only the following have been published so far (Scheme 1).

As a highly enantioselective synthesis, there has been a pioneering work by Linclau et al. They have reported that the asymmetric Sharpless dihydroxylation of readily available (*E*)-5-bromo-4,4,5,5-tetrafluoro-2-penten-1-ol derivative **6** led to the corresponding chiral diols **7** with an excellent enantiomeric excess, 96% ee (reaction 1 in Scheme 1) [20–21]. It has also been published that the asymmetric conjugate addition of 4-methylphenylboronic acid towards (*E*)-5-bromo-4,4,5,5-tetrafluoro-1-phenyl-2-penten-1-one (**8**) in the presence of a rhodium catalyst coordinated with (*S*)-BINAP gave the corresponding Michael adduct **9** in 94% enantiomeric excess (reaction 2, Scheme 1) [22].

As a diastereoselective synthesis, reductive coupling reactions of commercially available 4-bromo-3,3,4,4-tetrafluoro-1-butene and glyceraldehyde **10a**, its imine derivative **11**, or Garner's aldehyde **10b** have been reported [23–24]. Although the diastereoselectivities were low in some cases, the diastereomers **12** and **13** are often easily separable, and each diastereomer of optically active alcohols or amines can be obtained with an excellent optical purity (reactions 3 and 4 in Scheme 1).

To the best of our knowledge, these are the only four works for the preparation of optically active substances having a tetrafluoroethylene group on an asymmetric carbon center. In order to overcome the current lack of synthetic methods for preparing such molecules, we came up with the idea of utilizing the [1,3]-proton shift reaction reported by Soloshonok et al.

**Scheme 2:**
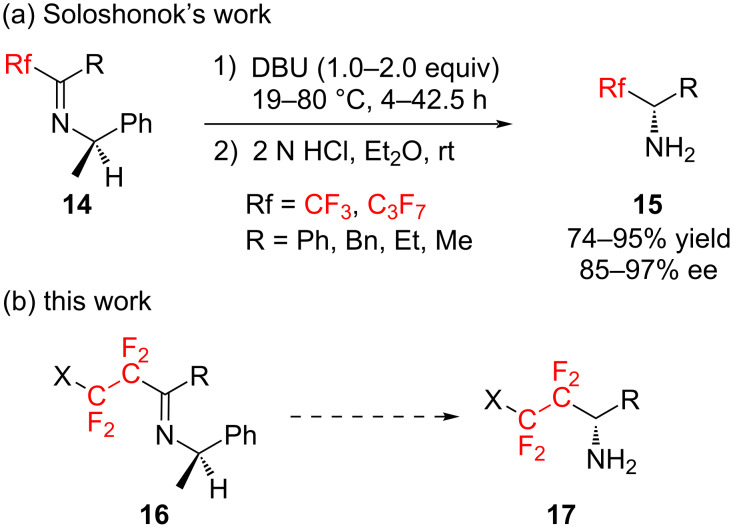
Synthetic strategy for preparing fluorine-containing amines via [1,3]-proton shift reactions.

In 1997, Soloshonok et al. reported that fluoroalkylated amines **15** with high optical purity could be easily prepared through [1,3]-proton shift reactions of optically active imines **14** which in turn were readily synthesized by condensation of various perfluoroalkyl ketones with optically active (*R*)-1-phenylethylamine (Scheme 2a) [25–32]. Therefore, we envisioned that optically active tetrafluoroethylenated amines **17** could be synthesized by applying the [1,3]-proton shift to optically active imines **16** derived from readily prepared tetrafluoroethylenated ketones (Scheme 2b).

In this paper, we describe the details of the [1,3]-proton shift reaction of various tetrafluoroethylenated imines.

## Results and Discussion

**Scheme 3:**
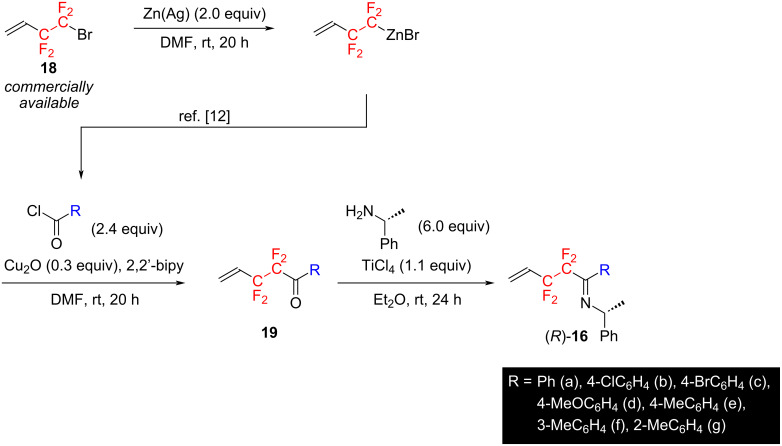
Preparation of the substrates used in this study.

The preparation of substrates used in this study was as outlined in Scheme 3. Namely, the zinc reagent was prepared from commercially available 3,3,4,4-tetrafluoro-1-butene (**18**) [33] and reacted with various acid chlorides in the presence of a copper catalyst to afford the corresponding tetrafluoroethylenated ketones **19**. The ketones were then condensed with (*R*)-1-phenylethylamine under the influence of TiCl_4_ [34–35] to prepare various optically active imines (*R*)-**16** in high yields (Scheme 3). Based on the result of the NOESY spectrum of the imine (*R*)-**16c,** the stereochemistry of the imines (*R*)-**16** was determined as *E* [36].

Among the imines thus obtained, (*R*)-**16b** was used to investigate the optimum reaction conditions (Table 1). Treatment of (*R*)-**16b** with 1.2 equiv of DBU (1,8-diazabicyclo[5.4.0]undec-7-ene) in THF at room temperature for 24 h gave the corresponding [1,3]-proton shift adduct (*S*)-**20b** in 31% yield (Table 1, entry 1). In this case, the HF-elimination product **21b** was also obtained in 16% [37], and the starting material was recovered in 53%. As shown in entries 2–7 of Table 1, the reactions in various solvents were next examined. When CH_3_CN or CH_2_Cl_2_ was used, 17% or 36% of the target product (*S*)-**20b** were obtained and almost no HF-elimination product **21b** was formed, while about 40% of the azocine derivative **22b** was afforded as a byproduct [38], along with the recovery of (*R*)-**16b**. In the case of diethyl ether, toluene, hexane, and cyclohexane, (*S*)-**20b** was obtained in 30% to 40% yield and significant amounts of unreacted substrate were still observed, although formation of the byproduct **22b** could be generally suppressed.

**Table 1:** Investigation of the reaction conditions.

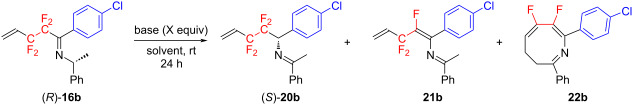						

Entry	Base/X equiv	Solvent	Yield^a^ [%] of (*S*)-**20b**	Yield^a^ [%] of **21b**	Yield^a^ [%] of **22b**	Recovery^a^ [%] of (*R*)-**16b**

1	DBU/1.2	THF	31	16	0	53
2	DBU/1.2	CH_3_CN	17	0	39	44
3	DBU/1.2	Et_2_O	38	14	17	31
4	DBU/1.2	toluene	37	13	0	50
5	DBU/1.2	hexane	26	12	0	62
6	DBU/1.2	CH_2_Cl_2_	36	3	38	23
7	DBU/1.2	cyclohexane	32	17	0	51
8	DBU/1.2	toluene	33	18	0	49
9	Et_3_N/1.2	toluene	0	0	0	100
10	DABCO/1.2	toluene	0	0	0	100
11	DBU/2.4	toluene	50	2	43	5
12	DBU/4.8	toluene	29	0	71	0
13	DBU/6.8	toluene	31	0	69	0
14^b^	DBU/2.4	toluene	8	13	0	79

^a^Determined by ^19^F NMR spectroscopy; ^b^the reaction was carried out at 0 °C.

We also examined the reaction using other bases instead of DBU. As shown in entries 9 and 10 of Table 1, the reaction did not proceed at all with triethylamine or DABCO, and (*R*)-**16b** was quantitatively recovered. The influence of the amount of DBU upon the reaction was also investigated (Table 1, entries 11–13). The results showed that when 2.4 equiv of DBU were used, the target compound (*S*)-**20b** was obtained in 50% yield, along with byproduct **22b** in 43% yield (Table 1, entry 11), while increasing the number of equivalents of DBU decreased the yield of the target product (*S*)-**20b** and increased the yield of the byproduct **22b** (Table 1, entries 12 and 13). Also carrying out the reaction at 0 °C gave unsatisfactory results and a large amount of (*R*)-**16b** was recovered (Table 1, entry 14).

Based on these results, the reaction conditions in entry 11 (Table 1) were determined as the optimum ones, which gave the highest yield, although the formation of the byproduct azocine derivative **22b** could not be completely suppressed.

**Scheme 4:**
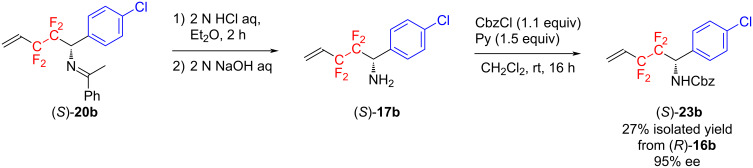
Derivatization of (*S*)-**20b** to (*S*)-**23b** for determining the optical purity of (*S*)-**20b**.

The thus obtained [1,3]-proton shift product (*S*)-**20b** was subjected to 2 N HCl aq in Et_2_O for 2 h, and subsequently 2 N NaOH aq, affording the corresponding free amine (*S*)-**17b**. Then, treatment of the amine with CbzCl and pyridine in CH_2_Cl_2_ gave the corresponding amide (*S*)-**23b** in 27% isolated yield over three-steps. The measurement of HPLC equipped with a chiral column, CHIRALPAK AD-H for (*S*)-**23b**, showed that the amide had an optical purity of 95% ee (Scheme 4).

**Scheme 5:**
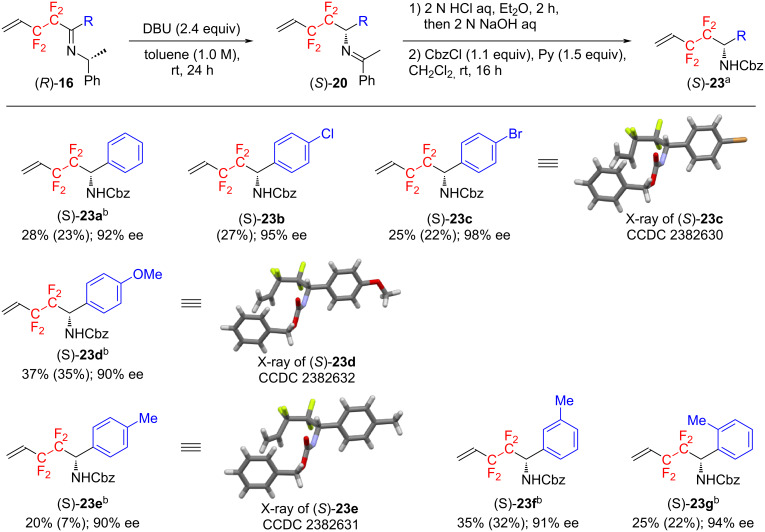
Substrate scope for the present [1,3]-proton shift reaction. ^a^Yields are determined by ^19^F NMR spectroscopy. Values in parentheses show isolated yields. Enantiomeric excesses are deteremined by HPLC equipped with DAICEL CHIRALPAK AD-H. ^b^Carried our at 50 °C in the [1,3]-proton shift reaction step.

On the next stage, the substrate scope for the present reaction was explored by using various imines (*R*)-**16** (Scheme 5). When the substituent R is an aromatic ring substituted by a halogen such as chlorine and bromine atoms, the amides (*S*)-**23** were obtained in 22–27% yield and with very high optical purity ((*S*)-**23b**, (*S*)-**23c**), although as for (*S*)-**23a**, a satisfactory result (92% ee) could be obtained when the reaction was performed at 50 °C. The substrates with an aromatic ring substituted by not only an electron-withdrawing halogen atom but also an electron-donating group such as methoxy and methyl group also smoothly underwent the [1,3]-proton shift reaction, affording the desired products with high enantiomeric excess (90% ee for (*S*)-**23d** and (*S*)-**23e**). Furthermore, it was found that the substituent position on the aromatic ring did not significantly influence the reaction efficiency as well as optical purity and the reaction proceeded in a highly enantioselective manner (91% ee for (*S*)-**23f** and 94% ee for (*S*)-**23g**). Disappointingly, when R is an alkyl group, the desired rearrangement products were rarely obtained, resulting in unidentified products together with a large amount of starting material. This may be due to the following reasons. Namely, when the substituent R is an aryl group, the negative charge generated on the imine carbon can be delocalized, hence the transition state is stabilized and the reaction proceeds smoothly. On the other hand, when the substituent R is an alkyl group, the negative charge generated on the imine carbon is an unstable factor, and therefore the transition state is not stabilized, leading to the increase of the activation energy of the reaction. As a result, the reaction does not proceed smoothly.

**Scheme 6:**
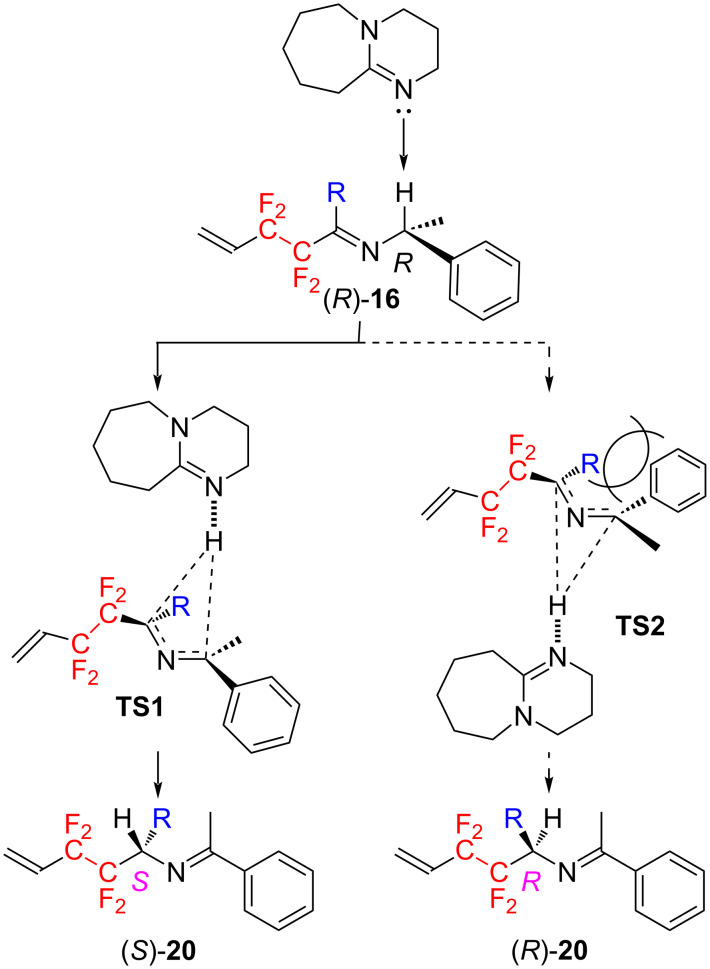
Proposed reaction mechanism.

The absolute configurations of product (*S*)-**23c**, (*S*)-**23d**, and (*S*)-**23e** were determined on the basis of their X-ray crystallographic analyses. The validity of their absolute configurations was confirmed by the convergence of the Flack parameters of three compounds to values close to 0, i.e. −0.006(12), 0.1(4) and 0.2(4), respectively. As indicated in Scheme 5, therefore, the absolute configurations of all compounds were determined as *S*. Then, the [1,3]-proton shift reaction in this study is expected to proceed via the reaction mechanism reported by Soloshonok [25–32], as shown in Scheme 6.

First, DBU interacts with the benzylic hydrogen of the imine (*R*)-**16**, and this hydrogen is about to be abstracted as a proton. This hydrogen which is being abstracted simultaneously interacts with the carbon possessing the tetrafluoroethylene fragment. At this time, transition states **TS1** or **TS2** are possible, but the reaction proceeds exclusively through the transition state **TS1** to avoid significant steric repulsion between the substituent R and the phenyl group. Therefore, the product (*S*)-**20** with *S* configuration is obtained preferentially.

## Conclusion

In summary, we have succeeded in synthesizing optically active amines having a tetrafluoroethylene group on the asymmetric carbon center by applying the [1,3]-proton shift reaction using various optically active imines, which can be easily prepared starting from commercially available 4-bromo-3,3,4,4-tetrafluoro-1-butene. In this reaction, although the formation of azocine derivatives as byproducts could not be completely suppressed, the [1,3]-proton shift reaction proceeded in relatively good yield and a high asymmetric transfer was achieved. As a result, it was found that various optically active amine derivatives could be obtained with high optical purity.

## Supporting Information

File 1 Full experimental details, ^1^H, ^13^C, ^19^F NMR spectra of **16a**–**g** and **23a**–**g**, and HPLC charts of racemic as well as chiral compounds **23a**–**g**.

File 2 Crystallographic information files (CIF) for compounds **23c**, **23d**, and **23e**.

## Data Availability

All data that supports the findings of this study will be available in the published article and/or the supporting information of this article.
