# Supplementary material for: Access to optically active tetrafluoroethylenated amines based on [1,3]-proton shift reaction
Source: Beilstein J Org Chem. 2024 Nov 1;20:2776–83. doi: 10.3762/bjoc.20.233 (PMC11533114; doi:10.3762/bjoc.20.233)
Supplement: File 1 — Full experimental details, 1H, 13C, 19F NMR spectra of 16a–g and 23a–g, and HPLC charts of racemic as well as chiral compounds 23a–g. [file Beilstein_J_Org_Chem-20-2776-s001.pdf]

BEILSTEIN JOURNAL OF ORGANIC CHEMISTRY

# Supporting Information

for

# Access to optically active tetrafluoroethylenated amines based on [1,3]-proton shift reaction

Yuta Kabumoto, Eiichiro Yoshimoto, Bing Xiaohuan, Masato Morita, Motohiro Yasui, Shigeyuki Yamada and Tsutomu Konno

*Beilstein J. Org. Chem.* **2024,** *20,* 2776–2783. doi:10.3762/bjoc.20.233

# Full experimental details, $^{1}H$, $^{13}C$, $^{19}F$ NMR spectra of 16a–g and 23a–g, and HPLC charts of racemic as well as chiral compounds 23a–g

License and Terms: This is a supporting information file under the terms of the Creative Commons Attribution License (https://creativecommons.org/licenses/by/4.0). Please note that the reuse, redistribution and reproduction in particular requires that the author(s) and source are credited and that individual graphics may be subject to special legal provisions.
The license is subject to the *Beilstein Journal of Organic Chemistry* terms and conditions: (https://www.beilstein-journals.org/bjoc/terms)

## Table of contents

Structural assignment of (*R*)-**16** .......... S4

Structural assignment of **21b** .......... S5

Structural assignment of **22b** .......... S8

Experimental section .......... S9

**Copies of $^{1}$H, $^{13}$C, and $^{19}$F NMR spectra for new compounds** .......... S19

$^{1}$H NMR Spectrum of (*R*)-*N*-(2,2,3,3-tetrafluoro-1-phenylpent-4-en-1-ylidene)-1-phenylethylamine ((*R*)-**16a**) .......... S19

$^{13}$C NMR Spectrum of (*R*)-*N*-(2,2,3,3-tetrafluoro-1-phenylpent-4-en-1-ylidene)-1-phenylethylamine ((*R*)-**16a**) .......... S19

$^{19}$F NMR Spectrum of (*R*)-*N*-(2,2,3,3-tetrafluoro-1-phenylpent-4-en-1-ylidene)-1-phenylethylamine ((*R*)-**16a**) .......... S20

$^{1}$H NMR Spectrum of (*R*)-*N*-(1-(4-chlorophenyl)-2,2,3,3-tetrafluoropent-4-en-1-ylidene)-1-phenyethylamine ((*R*)-**16b**) .......... S21

$^{13}$C NMR Spectrum of (*R*)-*N*-(1-(4-chlorophenyl)-2,2,3,3-tetrafluoropent-4-en-1-ylidene)-1-phenyethylamine ((*R*)-**16b**) .......... S21

$^{19}$F NMR Spectrum of (*R*)-*N*-(1-(4-chlorophenyl)-2,2,3,3-tetrafluoropent-4-en-1-ylidene)-1-phenyethylamine ((*R*)-**16b**) .......... S22

$^{1}$H NMR Spectrum of (*R*)-*N*-(1-(4-bromophenyl)-2,2,3,3-tetrafluoropent-4-en-1-ylidene)-1-phenyethylamine ((*R*)-**16c**) .......... S23

$^{13}$C NMR Spectrum of (*R*)-*N*-(1-(4-bromophenyl)-2,2,3,3-tetrafluoropent-4-en-1-ylidene)-1-phenyethylamine ((*R*)-**16c**) .......... S23

$^{19}$F NMR Spectrum of (*R*)-*N*-(1-(4-bromophenyl)-2,2,3,3-tetrafluoropent-4-en-1-ylidene)-1-phenyethylamine ((*R*)-**16c**) .......... S24

$^{1}$H NMR Spectrum of (*R*)-*N*-(2,2,3,3-tetrafluoro-1-(4-methoxyphenyl)pent-4-en-1-ylidene)-1-phenylethylamine ((*R*)-**16d**) .......... S25

$^{13}$C NMR Spectrum of (*R*)-*N*-(2,2,3,3-tetrafluoro-1-(4-methoxyphenyl)pent-4-en-1-ylidene)-1-phenylethylamine ((*R*)-**16d**) .......... S25

$^{19}$F NMR Spectrum of (*R*)-*N*-(2,2,3,3-tetrafluoro-1-(4-methoxyphenyl)pent-4-en-1-ylidene)-1-phenylethylamine ((*R*)-**16d**) .......... S26

$^{1}$H NMR Spectrum of (*R*)-*N*-(2,2,3,3-tetrafluoro-1-(4-methylphenyl)pent-4-en-1-ylidene)-1-phenylethylamine ((*R*)-**16e**) .......... S27

$^{13}$C NMR Spectrum of (*R*)-*N*-(2,2,3,3-tetrafluoro-1-(4-methylphenyl)pent-4-en-1-ylidene)-1-phenylethylamine ((*R*)-**16e**) .......... S27

$^{19}$F NMR Spectrum of (*R*)-*N*-(2,2,3,3-tetrafluoro-1-(4-methylphenyl)pent-4-en-1-ylidene)-1-phenylethylamine ((*R*)-**16e**) ........ S28
$^{1}$H NMR Spectrum of (*R*)-*N*-(2,2,3,3-tetrafluoro-1-(3-methylphenyl)pent-4-en-1-ylidene)-1-phenylethylamine ((*R*)-**16f**) ........ S29
$^{13}$C NMR Spectrum of (*R*)-*N*-(2,2,3,3-tetrafluoro-1-(3-methylphenyl)pent-4-en-1-ylidene)-1-phenylethylamine ((*R*)-**16f**) ........ S29
$^{19}$F NMR Spectrum of (*R*)-*N*-(2,2,3,3-tetrafluoro-1-(3-methylphenyl)pent-4-en-1-ylidene)-1-phenylethylamine ((*R*)-**16f**) ........ S30
$^{1}$H NMR Spectrum of (*R*)-*N*-(2,2,3,3-tetrafluoro-1-(2-methylphenyl)pent-4-en-1-ylidene)-1-phenylethylamine ((*R*)-**16g**) ........ S31
$^{13}$C NMR Spectrum of (*R*)-*N*-(2,2,3,3-tetrafluoro-1-(2-methylphenyl)pent-4-en-1-ylidene)-1-phenylethylamine ((*R*)-**16g**) ........ S31
$^{19}$F NMR Spectrum of (*R*)-*N*-(2,2,3,3-tetrafluoro-1-(2-methylphenyl)pent-4-en-1-ylidene)-1-phenylethylamine ((*R*)-**16g**) ........ S32
$^{1}$H NMR Spectrum of (*S*)-benzyl *N*-(2,2,3,3-tetrafluoro-1-phenylpent-4-en-1-yl) carbamate ((*S*)-**23a**) ........ S33
$^{13}$C NMR Spectrum of (*S*)-benzyl *N*-(2,2,3,3-tetrafluoro-1-phenylpent-4-en-1-yl) carbamate ((*S*)-**23a**) ........ S33
$^{19}$F NMR Spectrum of (*S*)-benzyl *N*-(2,2,3,3-tetrafluoro-1-phenylpent-4-en-1-yl) carbamate ((*S*)-**23a**) ........ S34
Chromatograph in HPLC for (*S*)-**23a** ........ S34
$^{1}$H NMR Spectrum of (*S*)-benzyl *N*-(2,2,3,3-tetrafluoro-1-(4-chlorophenylpent-4-en-1-yl) carbamate ((*S*)-**23b**) ........ S35
$^{13}$C NMR Spectrum of (*S*)-benzyl *N*-(2,2,3,3-tetrafluoro-1-(4-chlorophenylpent-4-en-1-yl) carbamate ((*S*)-**23b**) ........ S35
$^{19}$F NMR Spectrum of (*S*)-benzyl *N*-(2,2,3,3-tetrafluoro-1-(4-chlorophenylpent-4-en-1-yl) carbamate ((*S*)-**23b**) ........ S36
Chromatograph in HPLC for (*S*)-**23b** ........ S36
$^{1}$H NMR Spectrum of (*S*)-benzyl *N*-(2,2,3,3-tetrafluoro-1-(4-bromophenylpent-4-en-1-yl) carbamate ((*S*)-**23c**) ........ S37
$^{13}$C NMR Spectrum of (*S*)-benzyl *N*-(2,2,3,3-tetrafluoro-1-(4-bromophenylpent-4-en-1-yl) carbamate ((*S*)-**23c**) ........ S37
$^{19}$F NMR Spectrum of (*S*)-benzyl *N*-(2,2,3,3-tetrafluoro-1-(4-bromophenylpent-4-en-1-yl) carbamate ((*S*)-**23c**) ........ S38
Chromatograph in HPLC for (*S*)-**23c** ........ S38

$^{1}H$ NMR Spectrum of (*S*)-benzyl *N*-(2,2,3,3-tetrafluoro-1-(4-methoxyphenylpent-4-en-1-yl)carbamate ((*S*)-**23d**)) ........ S39
$^{13}C$ NMR Spectrum of (*S*)-benzyl *N*-(2,2,3,3-tetrafluoro-1-(4-methoxyphenylpent-4-en-1-yl)carbamate ((*S*)-**23d**)) ........ S39
$^{19}F$ NMR Spectrum of (*S*)-benzyl *N*-(2,2,3,3-tetrafluoro-1-(4-methoxyphenylpent-4-en-1-yl)carbamate ((*S*)-**23d**)) ........ S40
Chromatograph in HPLC for (*S*)-**23d** ........ S40
$^{1}H$ NMR Spectrum of (*S*)-benzyl *N*-(2,2,3,3-tetrafluoro-1-(4-methylphenylpent-4-en-1-yl) carbamate ((*S*)-**23e**) ........ S41
$^{13}C$ NMR Spectrum of (*S*)-benzyl *N*-(2,2,3,3-tetrafluoro-1-(4-methylphenylpent-4-en-1-yl) carbamate ((*S*)-**23e**) ........ S41
$^{19}F$ NMR Spectrum of (*S*)-benzyl *N*-(2,2,3,3-tetrafluoro-1-(4-methylphenylpent-4-en-1-yl) carbamate ((*S*)-**23e**) ........ S42
Chromatograph in HPLC for (*S*)-**23e** ........ S42
$^{1}H$ NMR Spectrum of (*S*)-benzyl *N*-(2,2,3,3-tetrafluoro-1-(3-methylphenylpent-4-en-1-yl) carbamate ((*S*)-**23f)** ........ S43
$^{13}C$ NMR Spectrum of (*S*)-benzyl *N*-(2,2,3,3-tetrafluoro-1-(3-methylphenylpent-4-en-1-yl) carbamate ((*S*)-**23f)** ........ S43
$^{19}F$ NMR Spectrum of (*S*)-benzyl *N*-(2,2,3,3-tetrafluoro-1-(3-methylphenylpent-4-en-1-yl) carbamate ((*S*)-**23f)** ........ S44
Chromatograph in HPLC for (*S*)-**23f** ........ S44
$^{1}H$ NMR Spectrum of (*S*)-benzyl *N*-(2,2,3,3-tetrafluoro-1-(2-methylphenylpent-4-en-1-yl) carbamate ((*S*)-**23g**) ........ S45
$^{13}C$ NMR Spectrum of (*S*)-benzyl *N*-(2,2,3,3-tetrafluoro-1-(2-methylphenylpent-4-en-1-yl) carbamate ((*S*)-**23g**) ........ S45
$^{19}F$ NMR Spectrum of (*S*)-benzyl *N*-(2,2,3,3-tetrafluoro-1-(2-methylphenylpent-4-en-1-yl) carbamate ((*S*)-**23g**) ........ S46
Chromatograph in HPLC for (*S*)-**23g** ........ S46

Structural assignment of (*R*)-**16**

The stereochemistry of (*R*)-**16** was determined based on the NOESY spectrum analysis of (*R*)-**16c**. First of all, the signals of the proton derived from the methyl group, the phenethylamine-derived methine proton $H_b$, and the $H_a$ proton at the *p*-$ClC_6H_4$ group, can be observed around 1.4 ppm, 4.5 ppm, and 7.1 ppm, respectively, in the proton spectrum of (*R*)-**16c**.

The NOESY spectrum shows cross-peaks between the methyl group protons and $H_a$ and between $H_a$ and $H_b$, indicating that the Ha proton is in three-dimensional proximity to the methyl group and Hb. Accordingly, the stereochemistry of the imine (*R*)-**16c** was determined as *anti*.

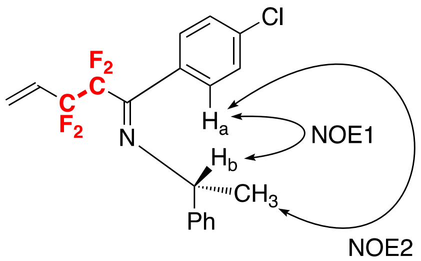


**(*R*)-16c**

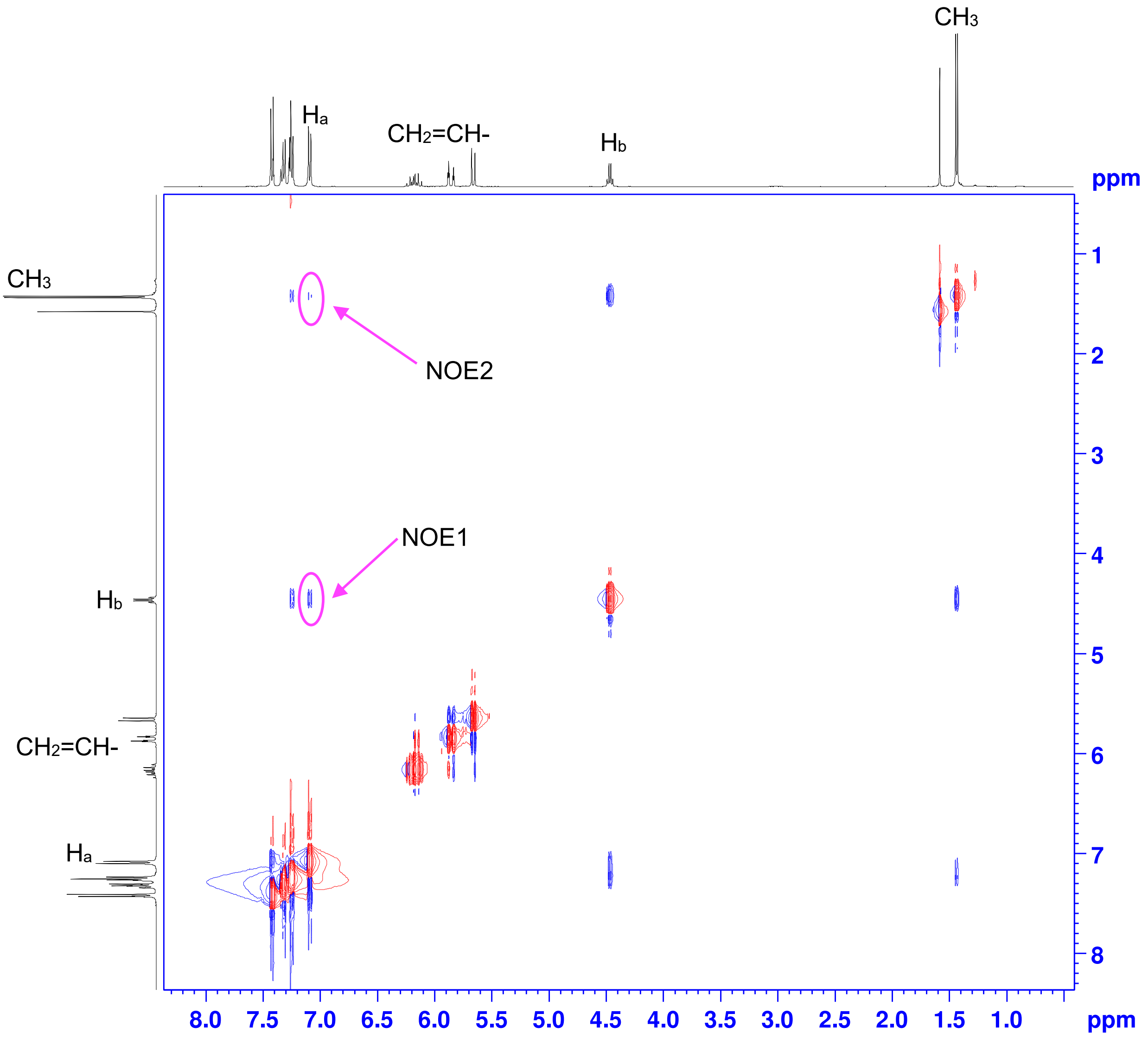

Structural assignment of **21b**

Spectra (a) and (b), shown below, are the $^{19}$F NMR ones of (*R*)-**16b** and **22b**, respectively. A spectrum (c) is the $^{19}$F NMR spectrum recorded immediately after the reaction finished when diethyl ether was used as a solvent (Table 2 Entry 3) in the investigation of the reaction conditions, and (d) is an expanded spectrum from −112 ppm to −116 ppm in (c).

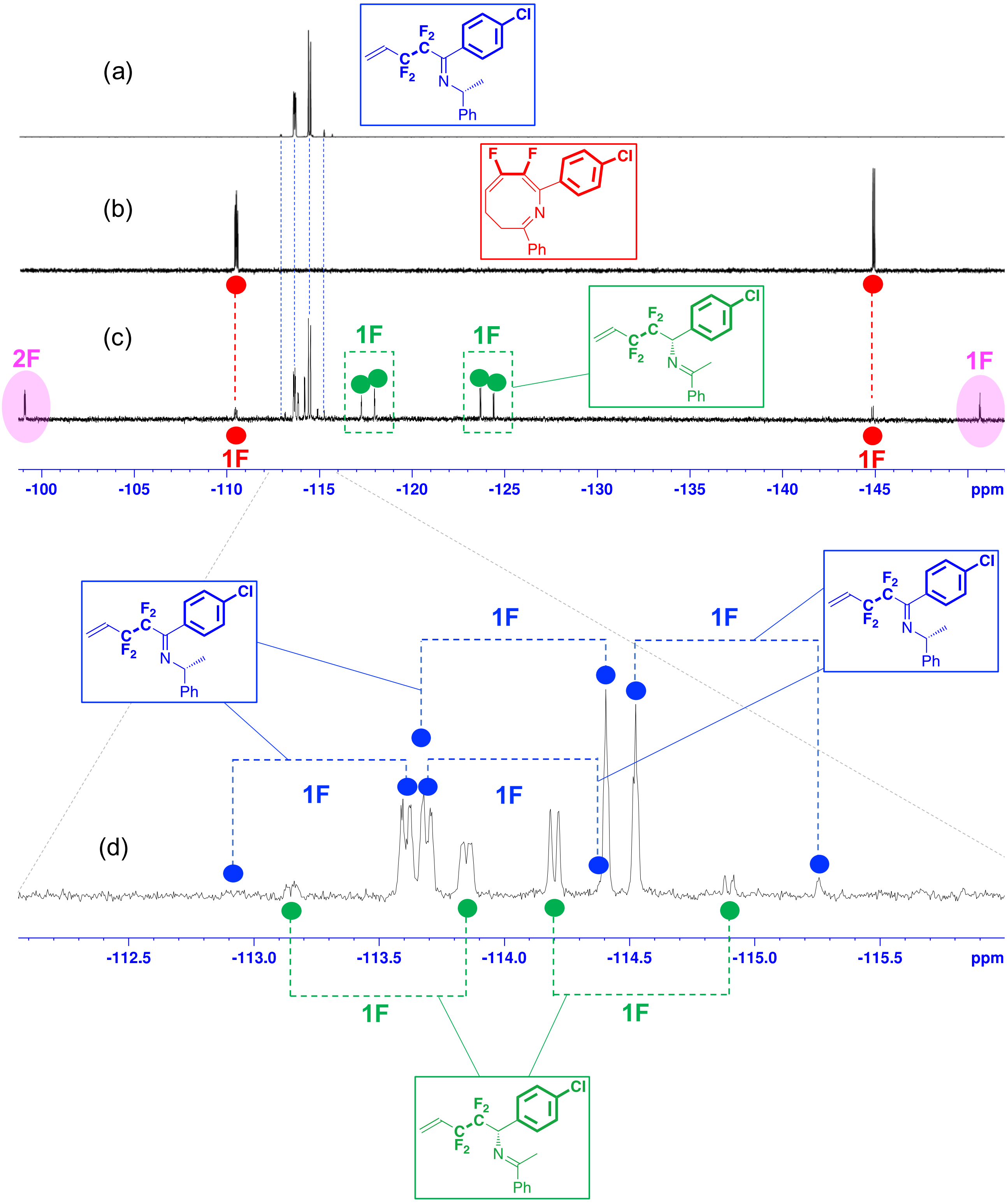

As can be seen from the $^{19}F$ NMR spectra, all signals except those marked in pink in the spectrum (c) can be attributed to (*R*)-**16b**, **20b**, and **22b**.
The signals marked in pink were analyzed as follows.

$^{19}F$ NMR ($CDCl_3$, $CFCl_3$): $\delta$ -99.10 to -99.00 (m, 1F), -150.66 (t, $J$ = 15.43 Hz, 2F).

This compound was found to be difficult to be isolated, and $^{1}H$ NMR and $^{13}C$ NMR measurements were not available.
On the other hand, it was possible to isolate *N*-benzylidene-1-phenyl-2,3,3-trifluoro-1,4-pentadienylamine, which was obtained *via* the reaction of *N*-(2,2,3,3-tetrafluoro-1-phenyl-4-pentenylidene)benzylamine with DBU. The structure of this compound could be unambiguously identified based on $^{1}H$ NMR and $^{13}C$ NMR spectra, as shown below.

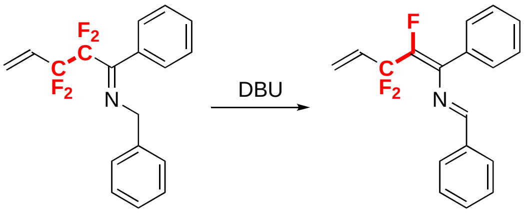


$^{1}H$ NMR ($CDCl_3$): $\delta$ 8.01 (s, 1H), 7.75 (d, $J$ = 6.39 Hz, 2H), 7.34-7.49 (m, 8H), 6.46 (tdd, $J$ = 17.38, 11.19, 10.79 Hz, 1H), 5.83 (td, $J$ = 17.38, 2.40 Hz, 1H), 5.55 (d, $J$ = 10.79 Hz, 1H); $^{13}C$ NMR ($CDCl_3$): $\delta$ 137.3 (dt, $J$ = 27.3, 4.1 Hz), 135.9, 132.1 (t, $J$ = 25.7 Hz), 131.7, 131.0, 129.7, 129.3 (d, $J$ = 3.3 Hz), 129.0, 128.9, 128.7, 128.7, 118.8 (t, $J$ = 9. Hz), 115.2 (dt, $J$ = 29.8, 239.7 Hz).

The $^{19}F$ NMR was also analyzed as follows.

$^{19}F$ NMR ($CDCl_3$, $CFCl_3$): $\delta$ -96.56 (t, $J$ = 14.67 Hz, 1F), -134.17 (t, $J$ = 14.67 Hz, 2F).

Despite some differences in chemical shifts, the $^{19}F$ NMR spectrum of *N*-benzylidene-1-phenyl-2,3,3-trifluoro-1,4-pentadienylamine is quite similar to the one marked in pink in spectrum (c).
Based on these results, the spectrum marked in pink in spectrum (c) was determined to be the HF eliminated product **21b**.

## $^{1}$H NMR Spectrum of *N*-(2,3,3-trifluoro-1-phenylpenta-1,4-dien-1-yl)benzylideneamine

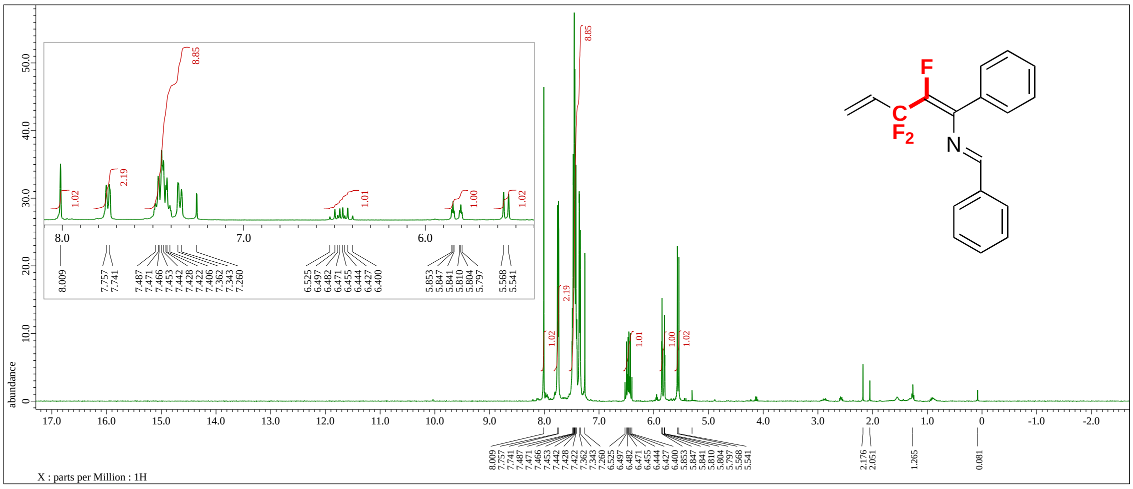


## $^{13}$C NMR Spectrum of *N*-(2,3,3-trifluoro-1-phenylpenta-1,4-dien-1-yl)benzylideneamine

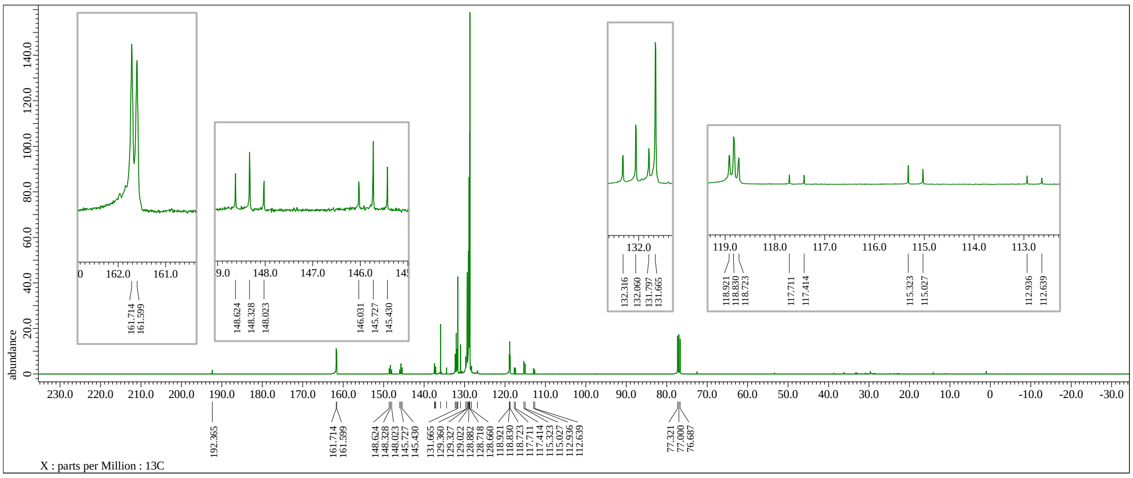


## $^{19}$F NMR Spectrum of *N*-(2,3,3-trifluoro-1-phenylpenta-1,4-dien-1-yl)benzylideneamine

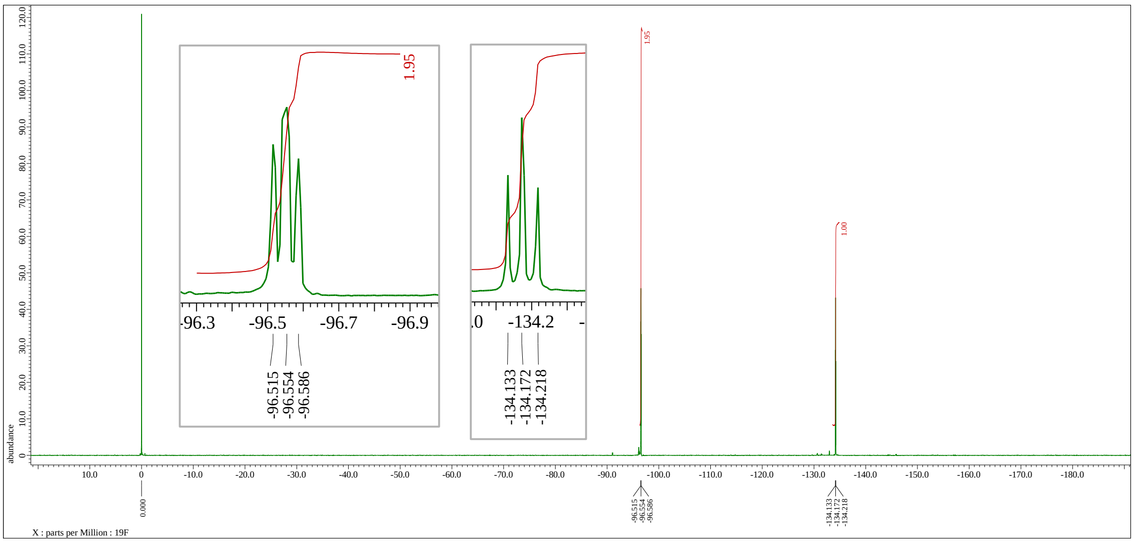

Structural assignment of **22b**

$^{1}$HNMR of azocine derivative **22b** and its analysis data are as follows.

$^{1}$H NMR Spectrum of 2-(4-chlorophenyl)-3,4-difluoro-6,7-dihydro-8-phenylazocine (**22b**)

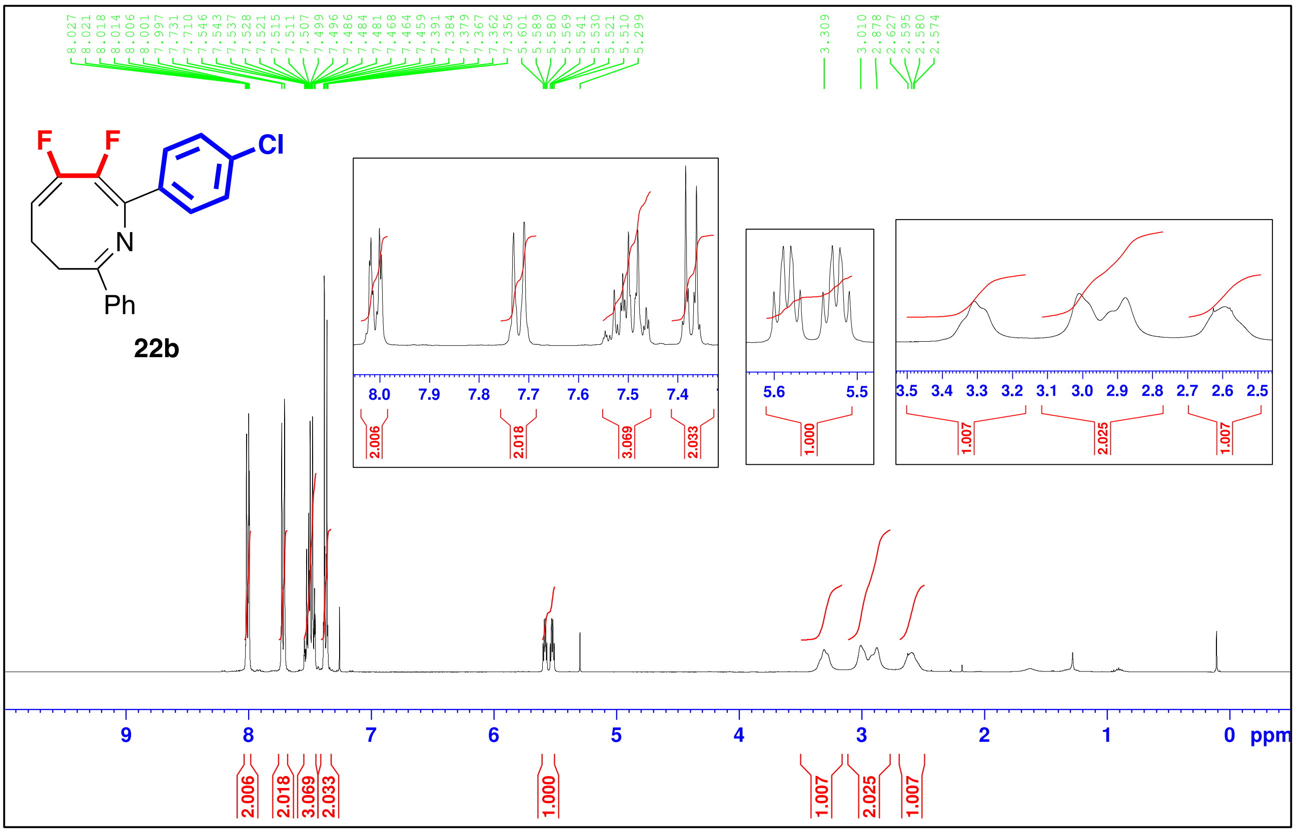


Yellow solid; M.P.: 114.0–114.3 °C (Hexane/AcOEt = 20/1, $R_f$ = 0.19); $^{1}$H NMR ($CDCl_3$): $\delta$ 7.99–8.02 (m, 2H, Ar–*H*), 7.70–7.72 (m, 2H, Ar–*H*), 7.48–7.52 (m, 3H, Ar–*H*), 7.36–7.38 (m, 2H, Ar–*H*), 5.56 (dtd, *J* = 23.69, 4.48, 3.64 Hz, 1H, CF=C*H*), 3.31 (brs, 1H, C$H_2$), 3.01 (brs, 1H, C$H_2$), 2.88 (brs, 1H, C$H_2$), 2.59 (brs, 1H, C$H_2$).

**Experimental**

**General information**

All air and/or moisture-sensitive reactions were carried out in anhydrous solvents under an Ar atmosphere in flame-dried glassware. All commercially available starting materials and reagents were used as received without further purification. Reactions were monitored by thin-layer chromatography (TLC) using Merck silica gel 60 $F_{254}$ plate, and column chromatography was carried out by using Wakogel® 60N (38–100 μm) as an adsorbent.

Specific optical rotations $[\alpha]_D$ were given in $10^{-1}$ deg $cm^2$ $g^{-1}$ and were measured using HORIBA SEPA-200 high sensitive polarimeter. Infrared spectra (IR) were determined in a liquid film on a NaCl plate or KBr method with a JASCO FT/IR-4100 type spectrometer, and reported in wavenumbers ($cm^{-1}$). High-resolution mass spectra (HRMS) were taken on a JEOL JMS-700MS spectrometer by fast atom bombardment (FAB) methods.

$^1$H NMR (400 MHz) and $^{13}$C NMR (100 MHz) were measured on a Bruker AVANCE III 400 NMR spectrometer, and chemical shifts were reported in parts per million (ppm, $\delta$) using the residual solvents peaks. Coupling constants ($J$) were reported in hertz (Hz). $^{19}$F NMR (376 MHz) was also measured on the Bruker AVANCE III 400 NMR spectrometer, and chemical shifts were reported ppm using hexafluorobenzene ($C_6F_6$) as an internal standard. $^{19}$F NMR spectra of the crude materials were used for determining the yield of the products with hexafluorobenzene ($C_6F_6$). HPLC was carried out on a Shimadzu LC-10AT *vp* Liquid Chromatograph equipped with a SPD-10A *vp* UV–vis detector using a chiral column (CHIRALPAK AD-H, DAICEL CEMICAL IND. Ltd., 0.46 cm $\phi$ × 25 cm).

**X-ray crystallography**

All measurements were made on a XtaLAB AFC10 diffractometer with filtered Mo Kα radiation (λ = 0.71073 Å) and a rotating anode generator using a VariMax with PILATUS/DW (Rigaku).

All calculations were performed using the CrysAlisPro ver. 1.171.41.117a (Rigaku, 2021) crystallographic software package. Empirical absorption corrections were applied using the SCALE 3 ABSPACK scaling algorithm (CrysAlisPro). The structure was solved by direct methods using SHELXT-2018/2 [1] and refined by a full-matrix least-squares method using SHELXL-2019/3 [2] visualized by Olex2 1.5 [3].

A colorless block crystal of (*R*)-**23c** having approximate dimensions of 0.52 × 0.24 × 0.19 mm was mounted on a Dual-Thickness MicroLoops LD (MiTeGen).

Crystal data for (*R*)-**23c**; orthorhombic, $a$ = 5.0939(5) Å, $b$ = 17.5523(12) Å, $c$ = 20.8204(14) Å, $V$ = 1861.5(3) Å$^3$, $T$ = 173 K, space group $P2_12_12_1$, $Z$ = 4 reflection measured. The final $R[(F^2>2\sigma(F^2)]$ and $wR(F^2)$ were 0.10 and 0.258. The flack's parameter was -0.006(12).

A colorless plate crystal of (*R*)-**23d** having approximate dimensions of 0.40 × 0.09 × 0.06 mm was mounted on a Dual-Thickness MicroLoops LD (MiTeGen).

Crystal data for (*R*)-**23d;** monoclinic, $a$ = 13.5582(6) Å, $b$ = 5,2746(2) Å, $c$ = 26.1316(11) Å, β = 101.708(5), $V$ = 1829.90(14) Å$^3$, $T$ = 173 K, space group *I*2, $Z$ = 4 reflection measured. The final $R[(F^2>2\sigma(F^2)]$ and $wR(F^2)$ were 0.039 and 0.097. The flack's parameter was -0.1(4).

A colorless plate crystal of (*R*)-**23e** having approximate dimensions of 0.53 × 0.16 × 0.09 mm was mounted on a Dual-Thickness MicroLoops LD (MiTeGen).

Crystal data for (*R*)-**23e**; monoclinic, $a$ = 13.4003(9) Å, $b$ = 5.1915(2) Å, $c$ = 26.9150(17) Å, β = 102.519(7), $V$ = 1827.89(19) Å$^3$, $T$ = 173 K, space group *I*2, $Z$ = 4 reflection measured. The final $R[(F^2>2\sigma(F^2)]$ and $wR(F^2)$ were 0.045 and 0.114. The flack's parameter was -0.2(4).

Crystallographic data for these compounds have been deposited with the Cambridge Crystallographic Data Centre as supplementary data no. CCDC 2382630 ((***R***)**-23c**), 2382632 ((***R***)**-23d**), and 2382631 ((***R***)**-23e**). Copy of the data can be obtained free of charge by applying to The Cambridge Crystallographic Data Centre, 12 Union Road, Cambridge CB2 1EZ, UK (https://summary.ccdc.cam.ac.uk/ structure-summary-form).

**Typical procedure for the preparation of imine (*R*)-16**

To a solution of the ketone **19a** (0.25 g, 1.09 mmol) and (*R*)-1-phenyethylamine (0.84 mL, 6.0 equiv, 6.54 mmol) in 5.0 mL of dry diethyl ether was added 0.13 mL of titanium (IV) chloride (1.1 equiv, 1.20 mmol) at 0 °C. After stirring the reaction mixture overnight, a mixture of 10 mL of an aqueous 0.5 M NaOH solution and 2.4 mL of diethyl ether was added into the reaction mixture. After separation of the organic phase, the aqueous phase was extracted with diethyl ether three times. The combined organic phases were dried over anhydrous sodium sulfate and the solvent was evaporated. The residue was purified by silica gel column chromatography to give the corresponding imine (*R*)-**16a** (0.30 g, 0.89 mmol, 82%).

**(*R*)-*N*-(2,2,3,3-Tetrafluoro-1-phenylpent-4-en-1-ylidene)-1-phenylethylamine ((*R*)-16a)**

Isolated yield: 82% (0.30 g, 0.89 mmol); Colorless oil; $[\alpha]^{28}{}_D$= +100.2 (*c* 1.44, $CHCl_3$); the solvent of the column chromatography, Hexane/AcOEt = 20/1, $R_f$ = 0.24; $^1$H NMR ($CDCl_3$): $\delta$ 7.15-7.50 (m, 10H, Ar-*H*), 6.24 (dq, $J$ = 17.21, 11.60 Hz, 1H, $CH_2$=C*H*$CF_2$), 5.90 (dt, $J$ = 17.21, 2.00 Hz, 1H, C$H_2$=CH$CF_2$), 5.67 (d, $J$ = 11.60 Hz, 1H, C$H_2$=CH$CF_2$), 4.55 (q, $J$ = 6.40 Hz, 1H, C=NC*H*($CH_3$)Ph), 1.47 (d, $J$ = 6.40 Hz, 3H, C=NCH(C$H_3$)Ph); $^{13}$C NMR ($CDCl_3$): $\delta$ 159.9 (t, $J$ = 27.5 Hz, *C*=NCH($CH_3$)Ph), 144.3 (Ar), 131.8 (Ar), 129.7 (Ar), 128.63 (Ar), 128.55 (Ar), 127.9 (t, $J$ = 23.9 Hz, $CH_2$=*C*H$CF_2$), 127.8 (Ar), 127.2 (Ar), 126.5 (Ar), 123.3 (t, $J$ = 9.7 Hz, *C*$H_2$=CH$CF_2$), 115.7 (tt, $J$ = 250.2, 32.8 Hz, *C*$F_2$), 113.1 (tt, $J$ = 255.4, 33.4 Hz, *C*$F_2$), 61.8 (C=N*C*H($CH_3$)Ph), 24.7 (C=NCH(*C*$H_3$)Ph); $^{19}$F NMR ($CDCl_3$): $\delta$ -112.51 (dd, $J$ = 261.64, 11.60 Hz, 1F, $CH_2$=CHC$F_2$$CF_2$), -113.20 (d, $J$ = 275.57 Hz, 1F, $CH_2$=CH$CF_2$C$F_2$), -113.30 (dd, $J$ = 261.64, 11.60 Hz, 1F,

$CH_2$=CHC$F_2$$CF_2$), -114.06 (d, $J$ = 275.57 Hz, 1F, $CH_2$=CH$CF_2$C$F_2$),; IR (neat): $\nu$ 3029, 2976, 2927, 1660, 1419, 1242, 1168, 1101, 1037, 1010, 866, 701 $cm^{-1}$; HRMS (FAB) Calcd for $C_{19}H_{18}F_4N$ $[M+H]^+$: 336.1375, Found 336.1366.

**(*R*)-*N*-(1-(4-Chlorophenyl)-2,2,3,3-tetrafluoropent-4-en-1-ylidene)-1-phenyethylamine ((*R*)-16b)**

Isolated yield: 86% (0.64 g, 1.74 mmol); Colorless oil; $[\alpha]^{31}_D$= +164.5 ($c$ 1.25, $CHCl_3$); the solvent of the column chromatography, Hexane/AcOEt = 20/1, $R_f$ = 0.26; $^1$H NMR ($CDCl_3$): $\delta$ 7.50 (d, $J$ = 8.80 Hz, 2H, Ar-*H*), 7.30-7.46 (m, 5H, Ar-*H*), 7.21 (d, $J$ = 8.80 Hz, 2H, Ar-*H*), 6.32 (dq, $J$ = 17.61, 11.60 Hz, 1H, $CH_2$=C*H*$CF_2$), 5.96 (dt, $J$ = 17.61, 2.40 Hz, 1H, C$H_2$=CH$CF_2$), 5.74 (d, $J$ = 11.60 Hz, 1H, C$H_2$=CH$CF_2$), 4.61 (q, $J$ = 6.40 Hz, 1H, C=NC*H*($CH_3$)Ph), 1.56 (d, $J$ = 6.40 Hz, 3H, C=NCH(C$H_3$)Ph); $^{13}$C NMR ($CDCl_3$): $\delta$ 158.6 (t, $J$ = 27.7 Hz, *C*=NCH($CH_3$)Ph), 143.9 (Ar), 135.8 (Ar), 130.0 (Ar), 129.2 (Ar), 128.8 (Ar), 128.6 (Ar), 127.5 (t, $J$ = 23.9 Hz, $CH_2$=*C*H$CF_2$), 127.1 (Ar), 126.3 (Ar), 123.3 (t, $J$ = 9.3 Hz, *C*$H_2$=CH$CF_2$), 115.4 (tt, $J$ = 250.2, 32.9 Hz, *C*$F_2$), 112.8 (tt, $J$ = 255.5, 33.7 Hz, *C*$F_2$), 61.9 (C=N*C*H($CH_3$)Ph), 24.6 (C=NCH(*C*$H_3$)Ph); $^{19}$F NMR ($CDCl_3$): $\delta$ -112.43 (dm, $J$ = 262.77 Hz, 1F, $CH_2$=CHC$F_2$$CF_2$), -113.19 (dm, $J$ = 262.77 Hz, 1F, $CH_2$=CHC$F_2$$CF_2$), -113.20 (d, $J$ = 275.19 Hz, 1F, $CH_2$=CH$CF_2$C$F_2$), -114.05 (d, $J$ = 275.19, 1F, $CH_2$=CH$CF_2$C$F_2$); IR (neat): $\nu$ 3060, 3032, 2973, 1662, 1595, 1491, 1243, 1093, 1011, 877, 821, 700 $cm^{-1}$; HRMS (FAB) Calcd for $C_{19}H_{17}{}^{35}ClF_4N$ $[M+H]^+$: 370.0986, Found 370.0994.

**(*R*)-*N*-(1-(4-Bromophenyl)-2,2,3,3-tetrafluoropent-4-en-1-ylidene)-1-phenyethylamine ((*R*)-16c)**

Isolated yield: 88% (0.62 g, 1.99 mmol); Colorless oil; $[\alpha]^{31}_D$= +88.0 ($c$ 1.30, $CHCl_3$); the solvent of the column chromatography, Hexane/AcOEt = 10/1, $R_f$ = 0.46; $^1$H NMR ($CDCl_3$): $\delta$ 7.57 (d, $J$ = 8.80 Hz, 2H, Ar-*H*), 7.22-7.35 (m, 5H, Ar-*H*), 7.02 (d, $J$ = 8.80 Hz, 2H, Ar-*H*), 6.17 (dq, $J$ = 17.21, 12.00 Hz, 1H, $CH_2$=C*H*$CF_2$), 5.85 (dt, $J$ = 17.21, 2.40 Hz, 1H, C$H_2$=CH$CF_2$), 5.65 (d, $J$ = 12.00 Hz, 1H, C$H_2$=CH$CF_2$), 4.46 (q, $J$ = 6.40 Hz, 1H, C=NC*H*($CH_3$)Ph), 1.42 (d, $J$ = 6.40 Hz, 3H, C=NCH(C$H_3$)Ph); $^{13}$C NMR ($CDCl_3$): $\delta$ 158.6 (t, $J$ = 27.6 Hz, *C*=NCH(C$H_3$)Ph), 143.8 (Ar), 131.7 (Ar), 130.5 (Ar), 129.4 (Ar), 128.6 (Ar), 127.5 (t, $J$ = 23.8 Hz, $CH_2$=*C*H$CF_2$), 127.1 (Ar), 126.3 (Ar), 124.1 (Ar), 123.4 (t, $J$ = 9.5 Hz, *C*$H_2$=CH$CF_2$), 115.4 (tt, $J$ = 250.1, 33.0 Hz, $CF_2$), 112.7 (tt, $J$ = 255.6, 33.9 Hz, $CF_2$), 61.9 (C=N*C*H($CH_3$)Ph), 24.6 (C=NCH(*C*$H_3$)Ph); $^{19}$F NMR ($CDCl_3$): $\delta$ -113.27 (dm, $J$ = 262.77 Hz, 1F, $CH_2$=CHC$F_2$$CF_2$), -114.04 (dm, $J$ = 262.77 Hz), -114.05 (d, $J$ = 275.57 Hz, 1F, $CH_2$=CH$CF_2$C$F_2$), -114.90 (d, $J$ = 275.57 Hz, 1F, $CH_2$=CH$CF_2$C$F_2$); IR (neat): $\nu$ 2974, 1726, 1653, 1589, 1486, 1451, 1419, 1393, 1243, 1167, 1104, 1072, 1010, 911, 877, 816, 736 $cm^{-1}$; HRMS (FAB) Calcd for $C_{19}H_{17}{}^{79}BrF_4N$ $[M^+]$: 413.0402, Found 413.0394.

**(*R*)-*N*-(2,2,3,3-Tetrafluoro-1-(4-methoxyphenyl)pent-4-en-1-ylidene)-1-phenylethylamine ((*R*)-16d)**

Isolated yield d: 72% (1.06 g, 2.90 mmol); Colorless oil; $[\alpha]^{28}_{D}$= +165.1 (*c* 1.10, $CHCl_3$); the solvent of the column chromatography, Hexane/AcOEt = 20/1, $R_f$ = 0.28; $^1$H NMR ($CDCl_3$): $\delta$ 7.35-7.55 (m, 5H, Ar-*H*), 7.28 (d, *J* = 8.80 Hz, 2H, Ar-*H*), 7.09 (d, *J* = 8.80 Hz, 2H, Ar-*H*), 6.39 (dq, *J* = 17.61, 11.60 Hz, 1H, $CH_2$=C*H*$CF_2$), 5.99 (dt, *J* = 17.61, 2.00 Hz, 1H, C$H_2$=CH$CF_2$), 5.75 (d, *J* = 11.60 Hz, 1H, C$H_2$=CH$CF_2$), 4.78 (q, *J* = 6.40 Hz, 1H, C=NC*H*($CH_3$)Ph), 3.91 (s, 3H, OC$H_3$), 1.61 (d, *J* = 6.40 Hz, 3H, C=NCH(C$H_3$)Ph); $^{13}$C NMR ($CDCl_3$): $\delta$ 160.4 (Ar), 159.5 (t, *J* = 27.4 Hz, *C*=NCH($CH_3$)Ph), 144.3 (Ar), 129.2 (Ar), 128.4 (Ar), 127.9 (t, *J* = 23.6 Hz, $CH_2$=*C*H$CF_2$), 126.9 (Ar), 126.3 (Ar), 123.6 (Ar), 122.9 (t, *J* = 9.6 Hz, *C*$H_2$=CH$CF_2$), 115.5 (tt, *J* = 250.2, 32.6 Hz, *C*$F_2$), 113.8 (Ar), 113.0 (tt, *J* = 255.4, 33.2 Hz, *C*$F_2$), 61.5 (C=N*C*H($CH_3$)Ph), 54.9 (O*C*$H_3$), 24.6 (C=NCH(*C*$H_3$)Ph); $^{19}$F NMR ($CDCl_3$): $\delta$ -112.54 (dm, *J* = 276.70 Hz, 1F, $CH_2$=CHC$F_2$$CF_2$), -113.28 (d, *J* = 274.44 Hz, 1F, $CH_2$=CH$CF_2$C$F_2$), -113.36 (dm, *J* = 276.70 Hz, 1F, $CH_2$=CHC$F_2$$CF_2$), -114.15 (d, *J* = 274.44 Hz, 1F, $CH_2$=CH$CF_2$C$F_2$); IR (neat): $\nu$ 2971, 2930, 1608, 1511, 1296, 1254, 1168, 1103, 1035, 1008, 700 $cm^{-1}$; HRMS (FAB) Calcd for $C_{20}H_{20}F_4NO$ $[M+H]^+$: 366.1481, Found 366.1479.

**(*R*)-*N*-(2,2,3,3-Tetrafluoro-1-(4-methylphenyl)pent-4-en-1-ylidene)-1-phenylethylamine ((*R*)-16e)**

Isolated yield: 63%; Colorless oil ; $[\alpha]^{28}_{D}$ = +148.9 (*c* 1.33, $CHCl_3$); the solvent of the column chromatography, Hexane/AcOEt = 20/1, $R_f$ = 0.24; $^1$H NMR ($CDCl_3$): $\delta$ 7.32–7.45 (m, 7H, Ar-*H*), 7.18 (d, *J* = 8.00 Hz, 2H, Ar-*H*), 6.33 (dq, *J* = 17.61, 11.60 Hz, 1H, $CH_2$=C*H*$CF_2$), 5.95 (dt, *J* = 17.61, 2.40 Hz, 1H, C$H_2$=CH$CF_2$), 5.72 (d, *J* = 11.60 Hz, 1H, C$H_2$=CH$CF_2$), 4.68 (q, *J* = 6.40 Hz, 1H, C=NC*H*($CH_3$)Ph), 2.49 (s, 3H, Ar-C$H_3$), 1.55 (d, *J* = 6.40 Hz, 3H, C=NCH(C$H_3$)Ph); $^{13}$C NMR ($CDCl_3$): $\delta$ 159.8 (t, *J* = 27.3 Hz, *C*=NCH($CH_3$)Ph), 144.2 (Ar), 139.6 (Ar), 129.1 (Ar), 128.7 (Ar), 128.5 (Ar), 127.8 (t, *J* = 25.1 Hz, $CH_2$=*C*H$CF_2$), 127.6 (Ar), 127.0 (Ar), 126.4 (Ar), 123.0 (t, *J* = 9.5 Hz, *C*$H_2$=CH$CF_2$), 115.5 (tt, *J* = 250.2, 32.8 Hz, *C*$F_2$), 112.9 (tt, *J* = 255.5, 33.4 Hz, *C*$F_2$), 61.6 (C=N*C*H($CH_3$)Ph), 24.6 (Ar-*C*$H_3$), 21.2 (C=NCH(*C*$H_3$)Ph); $^{19}$F NMR ($CDCl_3$): $\delta$ -113.36 (dm, *J* = 261.64 Hz, 1F, $CH_2$=CHC$F_2$$CF_2$), -114.11 (d, *J* = 274.44 Hz, 1F, $CH_2$=CH$CF_2$C$F_2$), -114.17 (dm, *J* = 261.64 Hz, 1F, $CH_2$=CHC$F_2$$CF_2$), -114.98 (d, *J* = 274.44 Hz, 1F, $CH_2$=CH$CF_2$C$F_2$); IR (neat): $\nu$ 3032, 2975, 2925, 1658, 1451, 1418, 1243, 1165, 1100, 1029, 1009, 876, 700 $cm^{-1}$; HRMS (FAB) Calcd for $C_{20}H_{20}F_4N$ $[M+H]^+$: 350.1532, Found 350.1532.

***N*-(2,2,3,3-Tetrafluoro-1-(3-methylphenyl)pent-4-en-1-ylidene)-1-phenylethylamine ((*R*)-16f)**

Isolated yield: 96%; Colorless oil; $[\alpha]^{31}_{D}$ = +154.4 (*c* 1.36, $CHCl_3$); the solvent of the column chromatography, Hexane/AcOEt = 10/1, $R_f$ = 0.47; $^1$H NMR ($CDCl_3$): $\delta$ 7.22–7.37 (m, 7H Ar-*H*),

6.94-6.99 (m, 2H, Ar-*H*), 6.22 (dq, *J* = 17.21, 12.00 Hz, 1H, $CH_2$=C*H*$CF_2$), 5.86 (dt, *J* = 17.21, 2.00 Hz, 1H, C*H*$_2$=CH$CF_2$), 5.65 (d, *J* = 12.00 Hz, 1H, C*H*$_2$=CH$CF_2$), 4.51 (q, *J* = 6.40 Hz, 1H, C=NC*H*($CH_3$)Ph), 2.38 (s, 3H, Ar-C*H*$_3$), 1.44 (d, *J* = 6.40 Hz, 3H, C=NCH(C*H*$_3$)Ph); $^{13}$C NMR ($CDCl_3$): $\delta$ 159.9 (t, *J* = 27.5 Hz, *C*=NCH($CH_3$)Ph), 144.3 (Ar), 138.2 (Ar), 131.7 (Ar), 130.2 (Ar), 128.4 (Ar), 128.3 (Ar), 128.1 (Ar), 127.9 (t, *J* = 23.7 Hz, $CH_2$=*C*H$CF_2$), 127.0 (Ar), 126.4 (Ar), 124.7 (Ar), 123.0 (t, *J* = 9.4 Hz, *C*H$_2$=CH$CF_2$), 115.5 (tt, *J* = 250.0, 32.6 Hz, $CF_2$), 112.9 (tt, *J* = 255.3, 33.5 Hz, $CF_2$), 61.6 (C=N*C*H($CH_3$)Ph), 24.6 (Ar-*C*H$_3$), 21.3 (C=NCH(*C*H$_3$)Ph); $^{19}$F NMR ($CDCl_3$): $\delta$ -113.31 (dm, *J* = 262.02 Hz, 1F, $CH_2$=CHC*F*$_2$$CF_2$), -113.94 (dm, *J* = 262.02 Hz, 1F, $CH_2$=CHC*F*$_2$$CF_2$), -113.94 (dm, *J* = 276.57 Hz, 1F, $CH_2$=CH$CF_2$C*F*$_2$), -114.82 (dm, *J* = 276.57 Hz, 1F, $CH_2$=CH$CF_2$C*F*$_2$); IR (neat): $\nu$ 3028, 2974, 2929, 1662, 1652, 1604, 1585, 1493, 1451, 1419, 1370, 1249, 1209, 1099, 1029, 1008, 959, 926, 841, 772, 717 cm$^{-1}$; MS (FAB): *m/z* 350 (M$^+$, 44), 272 (M$^+$-PhH, 14), 246 ([M+H]$^+$-Ph$C_2H_4$, 31), 105 (Ph$C_2H_4^+$, 100), 77 (Ph$^+$, 15).

**(*R*)-*N*-(2,2,3,3-Tetrafluoro-1-(2-methylphenyl)pent-4-en-1-ylidene)-1-phenylethylamine ((*R*)-16g)**

Isolated yield: 45%, This is a mixture of atropisomers.; Colorless oil; $[\alpha]^{28}_D$ = +96.7 (*c* 0.97, $CHCl_3$); the solvent of the column chromatography, Hexane/AcOEt = 10/1, $R_f$ = 0.54; $^1$H NMR ($CDCl_3$): $\delta$ 7.13-7.40 (m, 9H, Ar-*H*), **Major isomer**: 6.32 (dq, *J* = 17.21, 11.60 Hz, 1H, $CH_2$=C*H*$CF_2$), 5.91 (dt, *J* = 17.21, 2.40 Hz, 1H, C*H*$_2$=CH$CF_2$), 5.68 (d, *J* = 11.60 Hz, 1H, C*H*$_2$=CH$CF_2$), 4.31 (q, *J* = 6.40 Hz, 1H, C=NC*H*($CH_3$)Ph), 1.93 (s, 3H, Ar-C*H*$_3$), 1.36 (d, *J* = 6.40 Hz, 3H, C=NCH(C*H*$_3$)Ph); **Minor isomer**: 6.22 (dq, *J* = 17.21, 11.60 Hz, 1H, $CH_2$=C*H*$CF_2$), 5.84 (dt, *J* = 17.21, 2.40 Hz, 1H, C*H*$_2$=CH$CF_2$), 5.64 (d, *J* = 11.60 Hz, 1H, C*H*$_2$=CH$CF_2$), 4.34 (q, *J* = 6.40 Hz, 1H, C=NC*H*($CH_3$)Ph), 2.33 (s, 3H, Ar-C*H*$_3$), 1.48 (d, *J* = 6.40 Hz, 3H, C=NCH(C*H*$_3$)Ph); $^{13}$C NMR ($CDCl_3$): Only two signals were detected for a $CF_2CF_2$ unit. 115.6 (tt, *J* = 249.8, 32.5 Hz, $CF_2$), 113.1 (tt, *J* = 256.0, 32.6 Hz, $CF_2$); **Major isomer**: $\delta$ 160.3 (dd, *J* = 32.4, 27.8 Hz, *C*=NCH($CH_3$)Ph), 143.9 (Ar), 136.3 (Ar), 132.7 (Ar), 131.6 (Ar), 130.2 (Ar), 129.5 (Ar), 128.4 (Ar), 128.2 (t, *J* = 23.6 Hz, $CH_2$=*C*H$CF_2$), 127.5 (Ar), 126.5 (Ar), 125.7 (Ar), 122.8 (t, *J* = 9.5 Hz, *C*H$_2$=CH$CF_2$), 62.0 (C=N*C*H($CH_3$)Ph), 24.7 (Ar-*C*H$_3$), 19.3 (C=NCH(*C*H$_3$)Ph); **Minor isomer**: $\delta$ 160.8 (dd, *J* = 32.1, 25.6 Hz, *C*=NCH($CH_3$)Ph), 143.9 (Ar), 139.8 (Ar), 135.8 (Ar), 131.9 (Ar), 130.2 (Ar), 129.4 (Ar), 128.0 (t, *J* = 23.8 Hz, $CH_2$=*C*H$CF_2$), 127.7 (Ar), 127.0 (Ar), 126.4 (Ar), 125.6 (Ar), 123.0 (t, *J* = 9.7 Hz, *C*H$_2$=CH$CF_2$), 62.1 (C=N*C*H($CH_3$)Ph), 24.0 (Ar-*C*H$_3$), 19.7 (C=NCH(*C*H$_3$)Ph); $^{19}$F NMR ($CDCl_3$): $\delta$ **Major isomer**: -114.50 to -113.50 (m, 2F, $CH_2$=CHC*F*$_2$$CF_2$), -113.77 (dt, *J* = 281.59, 4.14 Hz, 1F, $CH_2$=CH$CF_2$C*F*$_2$), -115.60 (dt, *J* = 281.59, 4.52 Hz, 1F, $CH_2$=CH$CF_2$C*F*$_2$); **Minor isomer**: -112.67 (dm, *J* = 276.70 Hz, 1F, $CH_2$=CH$CF_2$C*F*$_2$), -113.38 (dm, *J* = 259.38 Hz, 1F, $CH_2$=CHC*F*$_2$$CF_2$), -114.21 (dm, *J* = 259.38 Hz, 1F, $CH_2$=CHC*F*$_2$$CF_2$), -116.58 (dt, *J* = 276.70, 4.14 Hz, 1F, $CH_2$=CH$CF_2$C*F*$_2$); IR (neat): $\nu$ 3064,

3029, 2975, 2929, 2868, 1710, 1653, 1602, 1494, 1451, 1419, 1385, 1370, 1354, 1305, 1242, 1204, 1166, 1104, 1049, 1007, 980, 959, 911, 879, 861, 764 $cm^{-1}$; MS (FAB): *m/z* 350 ($M^+$, 39), 272 ($M^+$-PhH, 8), 246 ($[M+H]^+$-$PhC_2H_4$, 24), 105 ($PhC_2H_4^+$, 100), 77 ($Ph^+$, 14).

**Typical procedure for the synthesis of carbamate (*S*)-23b via [1,3]-proton shift reaction**

To a solution of imine (*R*)-**16b** (0.37 g, 1.00 mmol) in toluene (1.0 mL) was added 0.36 mL of DBU (2.4 equiv, 2.40 mmol) at room temperature, and the mixture was stirred at that temperature for 24 h. Then, the whole was diluted with MeOH (5.0 mL) and to this mixture was added 2 N HCl aqueous solution (5.0 mL) at room temperature. After 2 h, a large amount of 2 N NaOH aqueous solution was added, and then the whole was extracted with diethyl ether three times. The combined organic layers were dried over anhydrous sodium sulfate and the solvent was evaporated. The residue was purified by silica gel column chromatography (elution: hexane/EtOAc 5:1) to give the corresponding amine (0.10 g, 0.39 mmol).

The above amine (0.10 g, 0.39 mmol) was dissolved in $CH_2Cl_2$, and benzyl chloroformate (0.061 mL, 1.1 equiv, 0.43 mmol) and pyridine (0.048 mL, 1.5 equiv, 0.59 mmol) was gradually added into the above solution at 0 °C. After stirring for 16 h at room temperature, the mixture was poured into crushed ice and diluted with $CH_2Cl_2$. The organic phase was separated, washed with water three times, dried over anhydrous sodium sulfate, and then evaporated in vacuo. The residue was purified by silica gel column chromatography to give the corresponding carbamate (*S*)-**23b** as a crystal (0.11 g, 0.27 mmol, 27% for three-step yield).

**(*S*)-Benzyl *N*-(2,2,3,3-tetrafluoro-1-phenylpent-4-en-1-yl)carbamate ((*S*)-23a)**

Isolated yield: 23% (three-step yield): White solid; M.P.: 80.7-82.2 °C; $[\alpha]^{28}_D$ = +16.9 (*c* 0.33, $CHCl_3$); Enantiomeric excess was established by HPLC analysis, ee = 92% [CHIRALPAK AD-H; Hexane/*i*-PrOH = 80/20, 254 nm, 0.7 mL/m ($t_{S\ isomer}$ = 11.3 min, $t_{R\ isomer}$ = 17.8 min)]; the solvent of the column chromatography, Hexane/AcOEt = 3/1, $R_f$ = 0.57; $^1$H NMR ($CDCl_3$): $\delta$ 7.30-7.43 (m, 10H, Ar-*H*), 5.94 (dq, *J* = 17.61, 11.60 Hz, 1H, $CH_2$=C*H*$CF_2$), 5.81 (d, *J* = 17.61 Hz, 1H, C*H*$_2$=CH$CF_2$), 5.62 (d, *J* = 11.60 Hz, 1H, C*H*$_2$=CH$CF_2$), 5.56-5.65 (m, 1H, CHN*H*Cbz), 5.37-5.53 (m, 1H, C*H*NHCbz), 5.15 (d, *J* = 12.00 Hz, 1H, NHCOOC*H*$_2$Ph), 5.07 (d, *J* = 12.00 Hz, 1H, NHCOOC*H*$_2$Ph); $^{13}$C NMR ($CDCl_3$): $\delta$ 155.2 (NH*C*OOCH$_2$Ph), 135.8 (Ar), 134.0 (Ar), 128.8 (Ar), 128.6 (Ar), 128.5 (Ar), 128.3 (Ar), 128.2 (Ar), 128.1 (Ar), 126.3 (t, *J* = 24.9 Hz, $CH_2$=*C*H$CF_2$), 124.0 (t, *J* = 9.1 Hz, *C*H$_2$=CH$CF_2$), 115.9 (tt, *J* = 256.8, 33.2 Hz, $CF_2$), 115.3 (tt, *J* = 250.9, 33.1 Hz, $CF_2$), 67.4 (NHCOO*C*H$_2$Ph), 55.0 (dd, *J* = 28.2, 22.3 Hz, *C*HNHCbz); $^{19}$F NMR ($CDCl_3$): $\delta$ -112.58 (dd, *J* = 263.52, 9.79 Hz, 1F, CH=CHC*F*$_2$$CF_2$), -114.49 (dd, *J* = 263.52, 9.79 Hz, 1F, CH=CHC*F*$_2$$CF_2$), -117.03 (d, *J* = 273.69 Hz, 1F, CH=CH$CF_2$C*F*$_2$), -122.80 (dd, *J* = 273.69, 19.58 Hz, 1F,

$CH{=}CHCF_2C\mathit{F}_2$); IR (KBr): $\nu$ 3355, 2960, 2923, 1694, 1540, 1326, 1251, 1184, 1112, 1043, 1029, 979, 711 $cm^{-1}$; HRMS (FAB) Calcd for $C_{19}H_{18}F_4NO_2$ $[M+H]^+$: 368.1274, Found 368.1270.

**(*S*)-Benzyl *N*-(2,2,3,3-tetrafluoro-1-(4-chlorophenylpent-4-en-1-yl)carbamate ((*S*)-23b)**

Isolated yield: 27% (three-step yield): White solid; M.P: 113.6-115.4 °C; $[\alpha]^{31}_D$ = +30.6 (*c* 1.08, $CHCl_3$); Enantiomeric excess was established by HPLC analysis, ee = 95% [CHIRALPAK AD-H; Hexane/*i*-PrOH = 80/20, 254 nm, 0.7 mL/m ($t_{S\ isomer}$ = 9.86 min, $t_{R\ isomer}$ = 15.1 min)]; the solvent of the column chromatography, Hexane/AcOEt = 5/1, $R_f$ = 0.34; $^1H$ NMR ($CDCl_3$): $\delta$ 7.25-7.39 (m, 9H, Ar-*H*), 5.93 (dq, *J* = 17.61, 11.20 Hz, 1H, $C\mathit{H}_2{=}C\mathit{H}CF_2$), 5.81 (d, *J* = 17.61 Hz, 1H, $C\mathit{H}_2{=}CHCF_2$), 5.64 (d, *J* = 11.20 Hz, 1H, $C\mathit{H}_2{=}CHCF_2$), 5.56 (br d, *J* = 9.20 Hz, 1H, N*H*Cbz), 5.35-5.50 (m, 1H, C*H*NHCbz), 5.14 (d, *J* = 12.40 Hz, 1H, $NHCOOC\mathit{H}_2Ph$), 5.08 (d, *J* = 12.40 Hz, 1H, $NHCOOC\mathit{H}_2Ph$); $^{13}C$ NMR ($CDCl_3$): $\delta$ 155.3 ($NH\mathit{C}OOCH_2Ph$), 135.9 (Ar), 135.1 (Ar), 132.8 (Ar), 129.8 (Ar), 129.1 (Ar), 128.7 (Ar), 128.5 (Ar), 128.4 (Ar), 126.3 (t, *J* = 24.2 Hz, $CH_2{=}\mathit{C}HCF_2$), 124.5 (t, *J* = 9.6 Hz, $\mathit{C}H_2{=}CHCF_2$), 115.9 (tt, *J* = 256.0, 34.6 Hz, $CF_2$), 115.4 (tt, *J* = 250.3, 33.3 Hz, $CF_2$), 67.8 ($NHCOO\mathit{C}H_2Ph$), 54.7 (t, *J* = 24.8 Hz, *C*HNHCbz); $^{19}F$ NMR ($CDCl_3$): $\delta$ -113.26 (dm, *J* = 270.67 Hz, 1F), -115.08 (dm, *J* = 270.67 Hz, 1F), -117.48 (d, *J* = 275.57 Hz 1F), -123.66 (dd, *J* = 275.57, 18.07 Hz, 1F); IR (KBr): $\nu$ 3744, 3347, 3038, 2980, 2778, 1699, 1652, 1597, 1532, 1493, 1457, 1419, 1377, 1335, 1264, 1180, 1117, 1040, 1014, 992, 941, 916, 857, 829, 787 $cm^{-1}$; HRMS (FAB) Calcd for $C_{19}H_{17}{}^{35}ClF_4NO_2$ $[M+H]^+$: 402.0884, Found 402.0879.

**(*S*)-Benzyl *N*-(2,2,3,3-tetrafluoro-1-(4-bromophenylpent-4-en-1-yl)carbamate ((*S*)-23c)**

Isolated yield: 22% (three-step yield): White solid; M.P.: 110.4-111.6 °C; $[\alpha]^{28}_D$ = +20.8 (*c* 0.92, $CHCl_3$); 98% ee [CHIRALPAK AD-H; Hexane/*i*-PrOH = 80/20, 254 nm, 0.7 mL/m ($t_{S\ isomer}$ = 14.6 min, $t_{R\ isomer}$ = 25.4 min)]; the solvent of the column chromatography, Hexane/AcOEt = 10/1, $R_f$ = 0.40; $^1H$ NMR ($CDCl_3$): $\delta$ 7.51 (d, *J* = 8.20 Hz, 2H, Ar-*H*), 7.30-7.39 (m, 5H, Ar-*H*), 7.22 (d, *J* = 8.20 Hz, 2H, Ar-*H*), 5.95 (dq, *J* = 17.21, 11.20 Hz, 1H, $CH_2{=}C\mathit{H}CF_2$), 5.81 (d, *J* = 17.21 Hz, 1H, $C\mathit{H}_2{=}CHCF_2$), 5.65 (d, *J* = 11.20 Hz, 1H, $C\mathit{H}_2{=}CHCF_2$), 5.50 (d, *J* = 8.80 Hz, 1H, N*H*Cbz), 5.32-5.46 (m, 1H, C*H*NHCbz), 5.14 (d, *J* = 12.00 Hz, 1H, $NHCOOC\mathit{H}_2Ph$), 5.07 (d, *J* = 12.00 Hz, $NHCOOC\mathit{H}_2Ph$); $^{13}C$ NMR ($CDCl_3$): $\delta$ 155.3 ($NH\mathit{C}OOCH_2Ph$), 135.9 (Ar), 133.3 (Ar), 132.0 (Ar), 130.1 (Ar), 128.7 (Ar), 128.5 (Ar), 128.3 (Ar), 126.3 (t, *J* = 18.6 Hz, $CH_2{=}\mathit{C}HCF_2$), 124.5 (t, *J* = 9.5 Hz, $\mathit{C}H_2{=}CHCF_2$), 123.3 (Ar), 115.8 (tt, *J* = 259.5, 32.4 Hz, $CF_2$), 115.4 (tt, *J* = 250.6, 33.6 Hz, $CF_2$), 67.7 ($NHCOO\mathit{C}H_2Ph$), 54.8 (t, *J* = 24.0 Hz, *C*HNHCbz); $^{19}F$ NMR ($CDCl_3$): $\delta$ -113.16 (dm, *J* = 265.78 Hz, 1F), -114.96 (dm, *J* = 265.78 Hz, 1F), -117.33 (d, *J* = 276.70 Hz 1F), -123.59 (dd, *J* = 276.70, 15.43 Hz, 1F); IR (KBr): $\nu$ 3350, 3090, 3066, 3035, 2976, 2778, 1692, 1531, 1491, 1456, 1419, 1409, 1377, 1331, 1269, 1181, 1103, 1040, 1010, 991, 964, 917, 828, 781 $cm^{-1}$; HRMS (FAB)

Calcd for $C_{19}H_{17}{}^{79}BRF_4NO_2$ $[M+H]^+$: 446.0379, Found 446.0380.

**(*S*)-Benzyl *N*-(2,2,3,3-tetrafluoro-1-(4-methoxyphenylpent-4-en-1-yl)carbamate ((*S*)-23d)**

Isolated yield: 35% (three-step yield): White solid; M.P.: 65.6-67.2 °C; $[\alpha]^{28}{}_D$ = +26.1 (*c* 0.51, $CHCl_3$); 90% ee [CHIRALPAK AD-H; Hexane/*i*-PrOH = 80/20, 254 nm, 0.7 mL/m ($t_{S\ isomer}$ = 19.7 min, $t_{R\ isomer}$ = 49.4 min)]; the solvent of the column chromatography, Hexane/AcOEt =3/1, $R_f$ = 0.39; $^1H$ NMR ($CDCl_3$): $\delta$ 7.30-7.38 (m, 5H, Ar-*H*), 7.26 (d, *J* = 8.20 Hz, 2H, Ar-*H*), 6.89 (d, *J* = 8.20 Hz, 2H, Ar-*H*), 5.91 (dq, *J* = 17.21, 11.20 Hz, 1H, $CH_2$=C*H*$CF_2$), 5.79 (d, *J* = 17.21 Hz, 1H, C*H*$_2$=$CHCF_2$), 5.61 (d, *J* = 11.20 Hz, 1H, C*H*$_2$=$CHCF_2$), 5.51 (br d, *J* = 10.00 Hz, 1H, N*H*Cbz), 5.32-5.47 (m, 1H, C*H*NHCbz), 5.14 (d, *J* = 12.100 Hz, 1H, NHCOOC*H*$_2$Ph), 5.07 (d, *J* = 12.00 Hz, 1H, NHCOOC*H*$_2$Ph), 3.81 (s, 3H, ArOC*H*$_3$); $^{13}C$ NMR ($CDCl_3$): $\delta$ 159.9 (NH*C*OOCH$_2$Ph), 155.2 (Ar), 135.9 (Ar), 129.5 (Ar), 128.5 (Ar), 128.3 (Ar), 128.2 (Ar), 126.4 (t, *J* = 24.2 Hz, $CH_2$=*C*H$CF_2$), 126.1 (Ar), 123.9 (t, *J* = 9.5 Hz), 116.0 (tt, *J* = 255.4, 36.1 Hz, $CF_2$), 115.3 (tt, *J* = 250.5, 32.4 Hz, $CF_2$), 114.1 (Ar), 67.4 (NHCOO*C*H$_2$Ph), 55.2 (ArO*C*H$_3$), 54.5 (dd, *J* = 28.4, 22.0 Hz, *C*HNHCbz); $^{19}F$ NMR ($CDCl_3$): $\delta$ -112.57 (dd, *J* = 264.65, 10.16 Hz, 1F), -114.49 (dd, *J* = 264.65, 10.16 Hz, 1F), -117.48 (dd, *J* = 272.18, 7.91 Hz ,1F), -122.70 (dd, *J* = 272.18, 17.32 Hz, 1F); IR (KBr): $\nu$ 3371, 2963, 1699, 1533, 1516, 1243, 1122, 1093, 1038, 993, 798 $cm^{-1}$; HRMS (FAB) Calcd for $C_{19}H_{18}F_4NO_2$ $[M+H]^+$: 398.1379, Found 398.1366.

**(*S*)-Benzyl *N*-(2,2,3,3-tetrafluoro-1-(4-methylphenylpent-4-en-1-yl)carbamate ((*S*)-23e)**

Isolated yield: 7% (three-step yield): White solid; M.P.: 92.8-94.1 °C; $[\alpha]^{28}{}_D$ = +26.3 (*c* 0.07, $CHCl_3$); 90% ee [CHIRALPAK AD-H; Hexane/*i*-PrOH = 80/20, 254 nm, 0.7 mL/m ($t_{S\ isomer}$ = 10.5 min, $t_{R\ isomer}$ = 20.7 min)]; the solvent of the column chromatography, Hexane/AcOEt =3/1, $R_f$ = 0.62; $^1H$ NMR ($CDCl_3$): $\delta$ 7.30-7.40 (m, 5H, Ar-*H*), 7.25 (d, *J* = 8.00 Hz, 2H, Ar-*H*), 7.19 (d, *J* = 8.00 Hz, 2H, Ar-*H*), 5.94 (dq, *J* = 17.21, 11.20 Hz, 1H, C*H*$_2$=C*H*$CF_2$), 5.81 (d, *J* = 17.21 Hz, 1H, C*H*$_2$=$CHCF_2$), 5.62 (d, *J* = 11.20 Hz, 1H, C*H*$_2$=$CHCF_2$), 5.58 (br d, *J* = 10.00 Hz, 1H, N*H*Cbz), 5.35-5.49 (m, 1H, C*H*NHCbz), 5.15 (d, *J* = 12.00 Hz, 1H, NHCOOC*H*$_2$Ph), 5.07 (d, *J* = 12.00 Hz, 1H, NHCOOC*H*$_2$Ph), 2.37 (s, 3H, ArC*H*$_3$); $^{13}C$ NMR ($CDCl_3$): $\delta$ 155.2 (NH*C*OOCH$_2$Ph), 138.8 (Ar), 135.9 (Ar), 131.1 (Ar), 129.4 (Ar), 128.5 (Ar), 128.3 (Ar), 128.2 (Ar), 128.1 (Ar), 126.4 (t, *J* = 24.1 Hz, $CH_2$=*C*H$CF_2$), 124.0 (t, *J* = 9.6 Hz, *C*H$_2$=$CHCF_2$), 116.0 (tt, *J* = 257.4, 32.6 Hz, $CF_2$), 115.3 (tt, *J* = 250.5, 33.6 Hz, $CF_2$), 67.4 (NHCOO*C*H$_2$Ph), 54.8 (dd, *J* = 27.7, 21.9 Hz, *C*HNHCbz), 21.1 (Ar*C*H$_3$); $^{19}F$ NMR ($CDCl_3$): $\delta$ -112.52 (dd, *J* = 263.52, 9.79 Hz, 1F), -114.55 (dd, *J* = 263.52, 11.67 Hz, 1F), -117.23 (dm, *J* = 272.18 Hz ,1F), -122.82 (dd, *J* = 272.18, 18.07 Hz, 1F),; IR (KBr): $\nu$ 3366, 3037, 2962, 1704, 1324, 1244, 1208, 1098, 1034, 991, 791, 696 $cm^{-1}$; HRMS (FAB) Calcd for $C_{19}H_{18}F_4NO_2$ $[M+H]^+$: 382.1430, Found 382.1434.

**(*S*)-Benzyl N-(2,2,3,3-tetrafluoro-1-(3-methylphenyl)pent-4-en-1-yl)carbamate ((*S*)-23f)**

Isolated yield: 32% (three-step yield): White solid; M.P.: 73.5-75.3 °C; $[\alpha]^{28}_{D}$ = +43.1 (*c* 0.44, $CHCl_3$); 91% ee [CHIRALPAK AD-H; Hexane/*i*-PrOH = 80/20, 254 nm, 0.7 mL/m ($t_{S\ isomer}$ = 10.4 min, $t_{R\ isomer}$ = 15.1 min)]; the solvent of the column chromatography, Hexane/AcOEt =5/1, $R_f$ = 0.41; $^1$H NMR ($CDCl_3$): $\delta$ 7.15-7.44 (m, 9H, Ar-*H*), 5.97 (dq, *J* = 17.21, 11.60 Hz, 1H, $CH_2$=C*H*$CF_2$), 5.84 (d, *J* = 17.21 Hz, 1H, C*H*$_2$=CH$CF_2$), 5.70 (d, *J* = 9.60 Hz, 1H, CHN*H*Cbz), 5.63 (d, *J* = 11.60 Hz, 1H, C*H*$_2$=CH$CF_2$), 5.40-5.53 (m, 1H, C*H*NHCbz), 5.17 (d, *J* = 12.00 Hz, 1H, NH$CO_2$C*H*$_2$Ph), 5.09 (d, *J* = 12.00 Hz, 1H, NH$CO_2$C*H*$_2$Ph), 2.38 (s, 3H, Ar-C*H*$_3$); $^{13}$C NMR ($CDCl_3$): $\delta$ 155.2 (NH*C*OO$CH_2$Ph), 138.3 (Ar), 135.9 (Ar), 134.0 (Ar), 129.6 (Ar), 129.0 (Ar), 128.50 (Ar), 128.47 (Ar), 128.2 (Ar), 128.1 (Ar), 126.4 (t, *J* = 24.2 Hz, $CH_2$=*C*H$CF_2$), 125.2 (Ar), 123.9 (t, *J* = 9.6 Hz, *C*$H_2$=CH$CF_2$), 116.0 (tt, *J* = 256.6, 31.7 Hz, $CF_2$), 115.3 (tt, *J* = 250.6, 33.6 Hz, $CF_2$), 67.4 (NHCOO*C*$H_2$Ph), 55.0 (dd, *J* = 28.1, 21.5, Hz, *C*HNHCbz), 21.2 (Ar-*C*$H_3$); $^{19}$F NMR ($CDCl_3$): $\delta$ -113.03 (dd, *J* = 264.27, 10.16 Hz, 1F), -115.10 (dd, *J* = 264.27, 9.41 Hz, 1F), -117.33 (d, *J* = 272.56 Hz ,1F), -123.48 (dd, *J* = 272.56, 18.45 Hz, 1F); IR (KBr): $\nu$ 3354, 3034, 2961, 2898, 1683, 1530, 1465, 1455, 1415, 1335, 1247, 1171, 1118, 1045, 1009, 962, 913, 898, 855, 828, 779 cm$^{-1}$; HRMS (FAB) Calcd for $C_{19}H_{18}F_4NO_2$ $[M+H]^+$: 382.1430, Found 382.1426.

**(*S*)-Benzyl N-(2,2,3,3-tetrafluoro-1-(2-methylphenylpent-4-en-1-yl)carbamate ((*S*)-23g)**

Isolated yield: 22% (three-step yield): White solid; M.P.: 51.9-52.5 °C; $[\alpha]^{28}_{D}$ = +23.0 (*c* 0.43, $CHCl_3$); 94% ee [CHIRALPAK AD-H; Hexane/*i*-PrOH = 80/20, 254 nm, 0.7 mL/m ($t_{S\ isomer}$ = 9.3 min, $t_{R\ isomer}$ = 16.1 min)]; the solvent of the column chromatography, Hexane/AcOEt = 5/1 $R_f$ = 0.47; $^1$H NMR ($CDCl_3$): $\delta$ 7.20-7.38 (m, 9H, Ar-*H*), 5.95 (dq, *J* = 17.21, 10.80, Hz, 1H, $CH_2$=C*H*$CF_2$), 5.83 (d, *J* = 17.21 Hz, 1H, C*H*$_2$=CH$CF_2$), 5.75-5.85 (m, 1H, C*H*NHCbz), 5.62 (d, *J* = 10.80 Hz, 1H, C*H*$_2$=CH$CF_2$), 5.51 (d, *J* = 9.60 Hz, 1H, CHN*H*Cbz), 5.14 (d, *J* = 12.40 Hz, 1H, NHCOOC*H*$_2$Ph), 5.04 (d, *J* = 12.40 Hz, 1H, NHCOOC*H*$_2$Ph), 2.44 (s, 3H, Ar-C*H*$_3$); $^{13}$C NMR ($CDCl_3$): $\delta$ 155.2 (C=O), 137.1 (Ar), 135.9 (Ar), 133.1 (Ar), 130.7 (Ar), 128.7 (Ar), 128.6 (Ar), 128.3 (Ar), 128.2 (Ar), 127.1 (Ar), 126.37 (Ar), 126.35 (t, *J* = 24.3 Hz, $CH_2$=*C*H$CF_2$), 124.0 (t, *J* = 9.5 Hz, *C*$H_2$=CH$CF_2$), 116.2 (tt, *J* = 253.8, 33.3 Hz, $CF_2$), 115.3 (tt, *J* = 252.0, 33.6 Hz, $CF_2$), 67.5 ($CO_2$*C*$H_2$Ph), 50.1 (dd, *J* = 29.4, 21.5 Hz, *C*HNHCbz), 19.5 (Ar-*C*$H_3$); $^{19}$F NMR ($CDCl_3$): $\delta$ -114.10 (dm, *J* = 270.67 Hz, 1F), -115.87 (dm, *J* = 270.67 Hz, 1F), -117.67 (d, *J* = 275.57 Hz ,1F), -124.70 (dm, *J* = 275.57 Hz, 1F); IR (KBr): $\nu$ 3382, 3037, 2963, 2925, 1707, 1532, 1451, 1421, 1377, 1343, 1321, 1248, 1189, 1126, 1095, 1035, 1011, 987, 957, 917, 824, 748 cm$^{-1}$; HRMS (FAB) Calcd for $C_{20}H_{19}F_4NNaO_2$ $[M+Na]^+$: 404.1250, Found 404.1241.

**References**


1. Sheldrick, G. M. *Acta Crystallogr., Sect. A: Struct. Chem*. **2015**, *71*, 3-8.
2. Sheldrick, G. M. *Acta Crystallogr., Sect. C: Struct. Chem*. **2015**, 71, 3-8.
3. Dolomanov, O. V.; Bourhis, L. J.; Gildea, R. J.; Howard , J. A. K.; Puschmann, H. *J. Appl. Crystallogr.* **2009**, *42*, 339–341.

$^{1}$H NMR Spectrum of (*R*)-*N*-(2,2,3,3-tetrafluoro-1-phenylpent-4-en-1-ylidene)-1-phenylethylamine ((*R*)-**16a**)

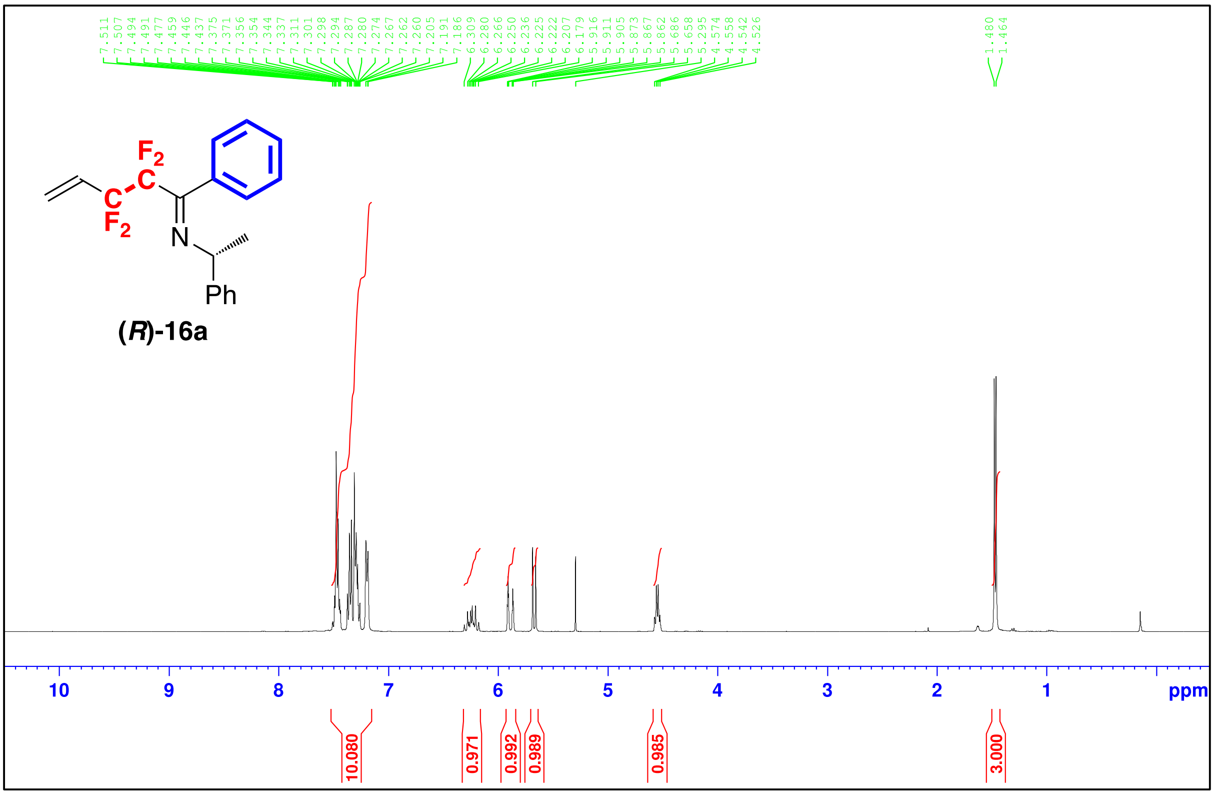


$^{13}$C NMR Spectrum of (*R*)-*N*-(2,2,3,3-tetrafluoro-1-phenylpent-4-en-1-ylidene)-1-phenylethylamine ((*R*)-**16a**)

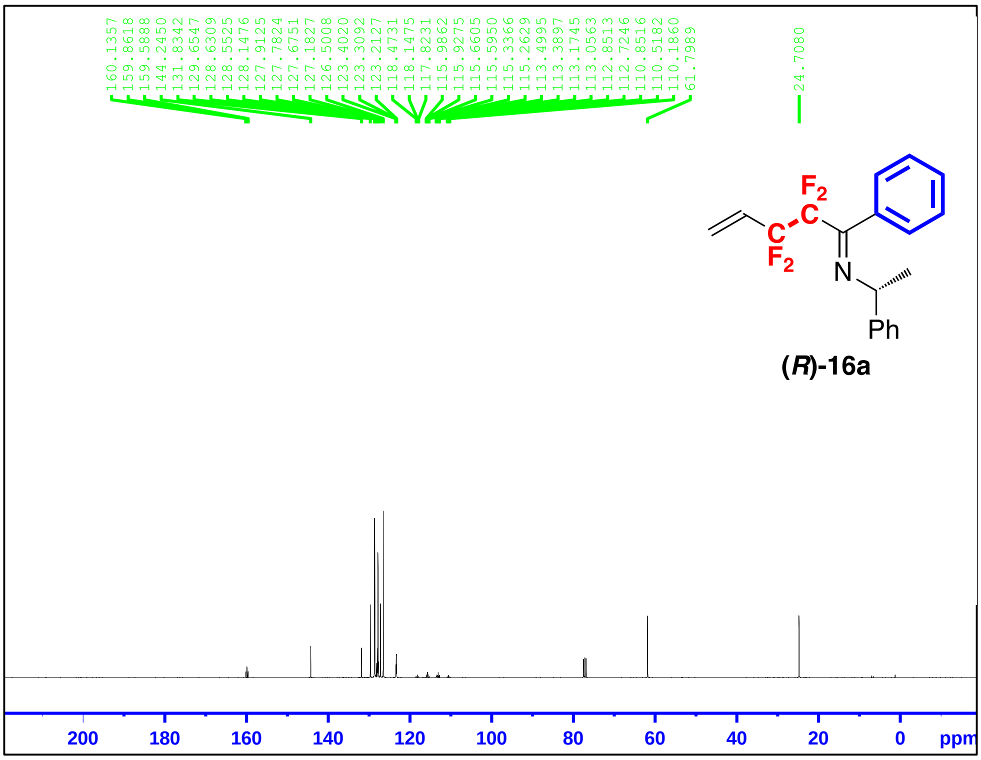

$^{19}$F NMR Spectrum of (*R*)-*N*-(2,2,3,3-tetrafluoro-1-phenylpent-4-en-1-ylidene)-1-phenylethylamine ((*R*)-**16a**)

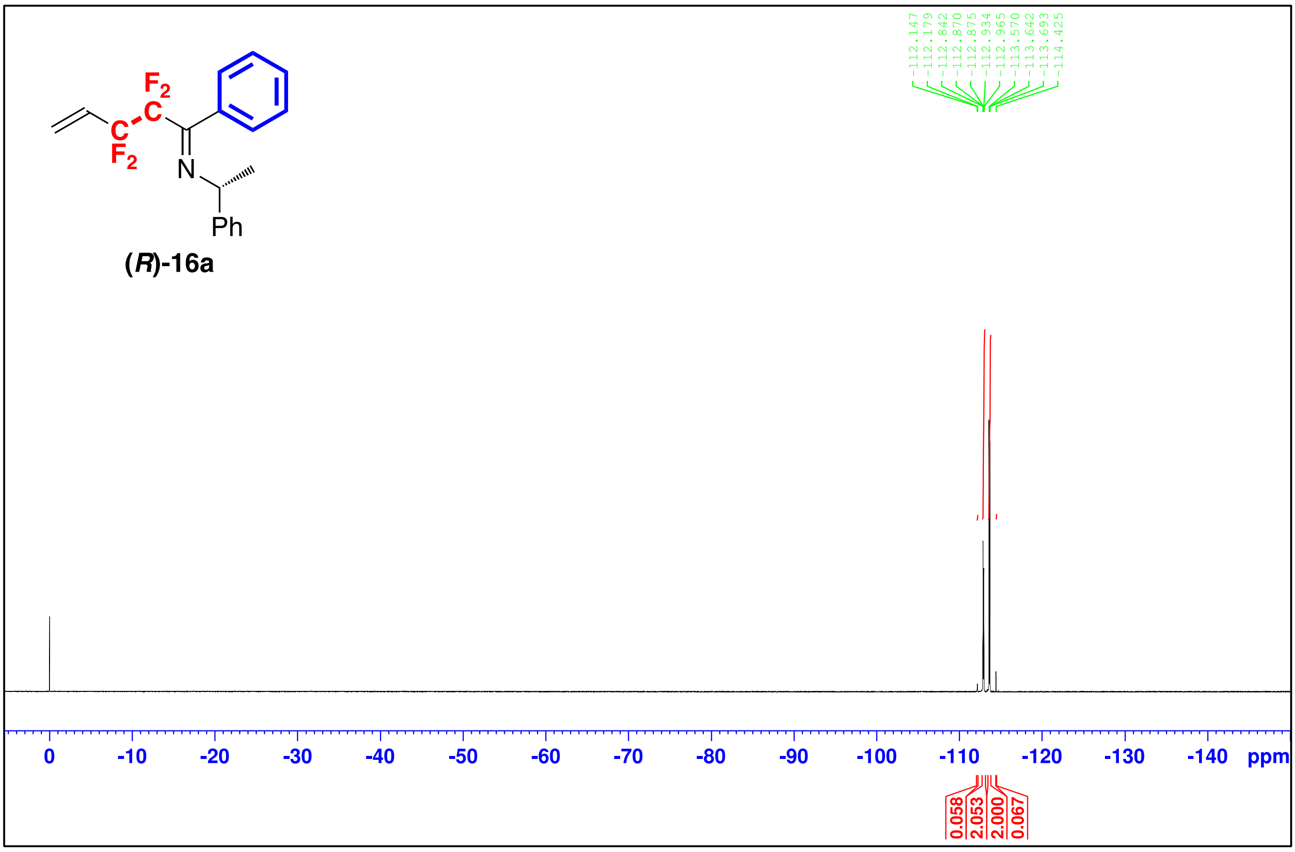

$^{1}H$ NMR Spectrum of (*R*)-*N*-(1-(4-chlorophenyl)-2,2,3,3-tetrafluoropent-4-en-1-ylidene)-1-phenyethylamine ((*R*)-**16b**)

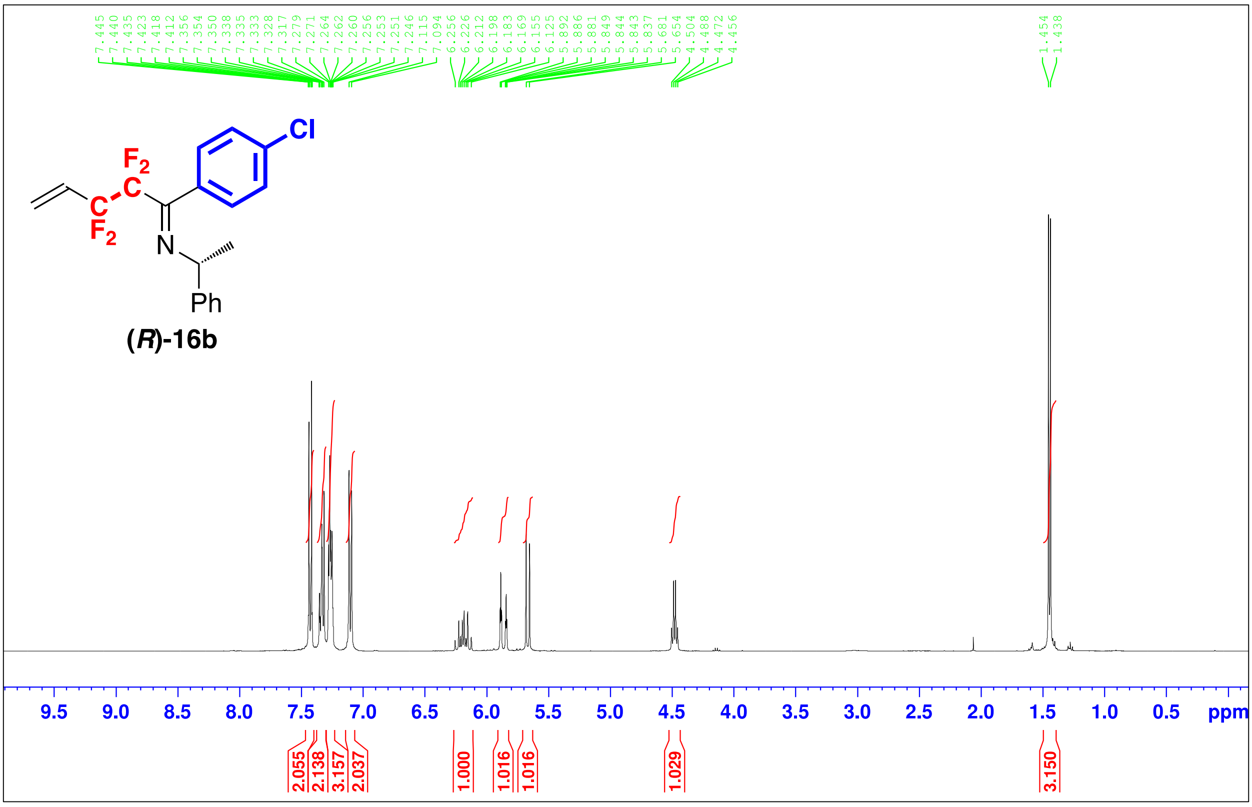


$^{13}C$ NMR Spectrum of (*R*)-*N*-(1-(4-chlorophenyl)-2,2,3,3-tetrafluoropent-4-en-1-ylidene)-1-phenyethylamine ((*R*)-**16b**)

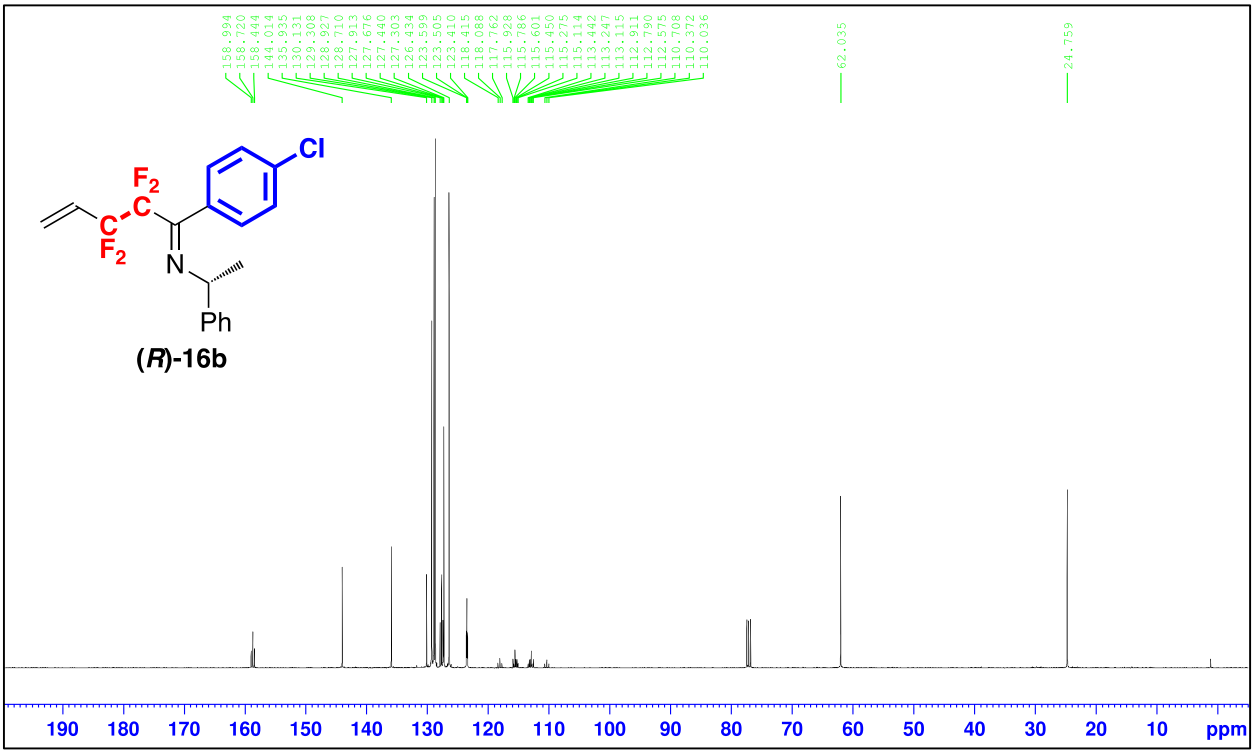

$^{19}$F NMR Spectrum of (*R*)-*N*-(1-(4-chlorophenyl)-2,2,3,3-tetrafluoropent-4-en-1-ylidene)-1-phenyethylamine ((*R*)-**16b**)

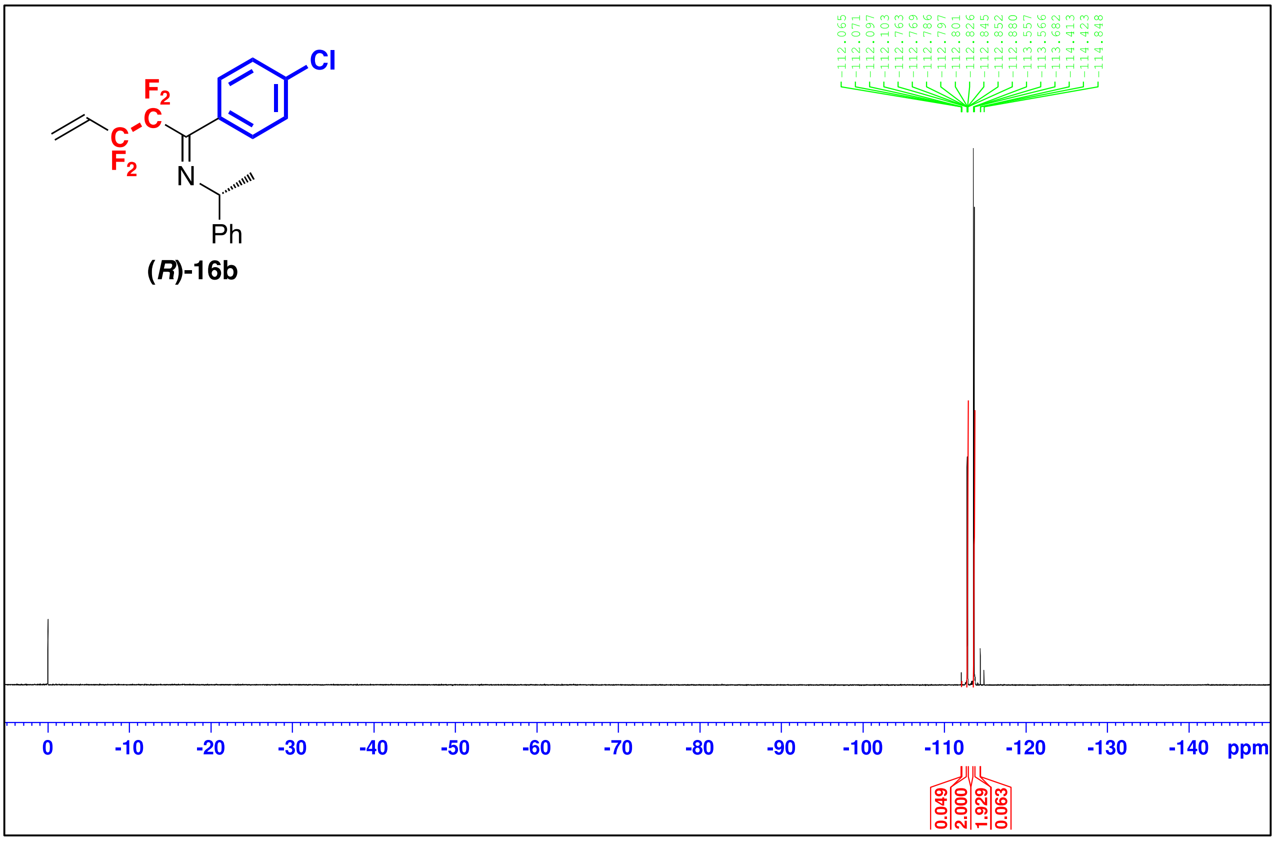

$^{1}$H NMR Spectrum of (*R*)-*N*-(1-(4-bromophenyl)-2,2,3,3-tetrafluoropent-4-en-1-ylidene)-1-phenyethylamine ((*R*)-**16c**)

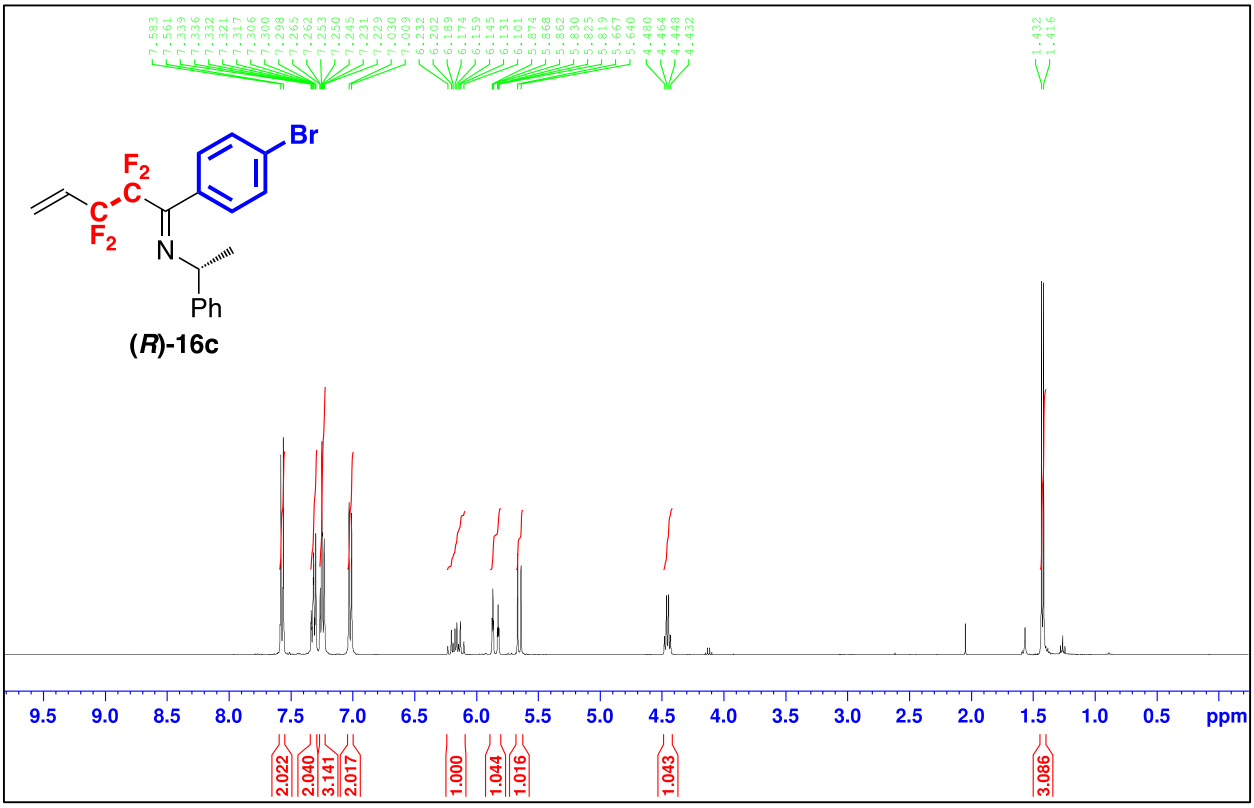

$^{13}$C NMR Spectrum of (*R*)-*N*-(1-(4-bromophenyl)-2,2,3,3-tetrafluoropent-4-en-1-ylidene)-1-phenyethylamine ((*R*)-**16c**)

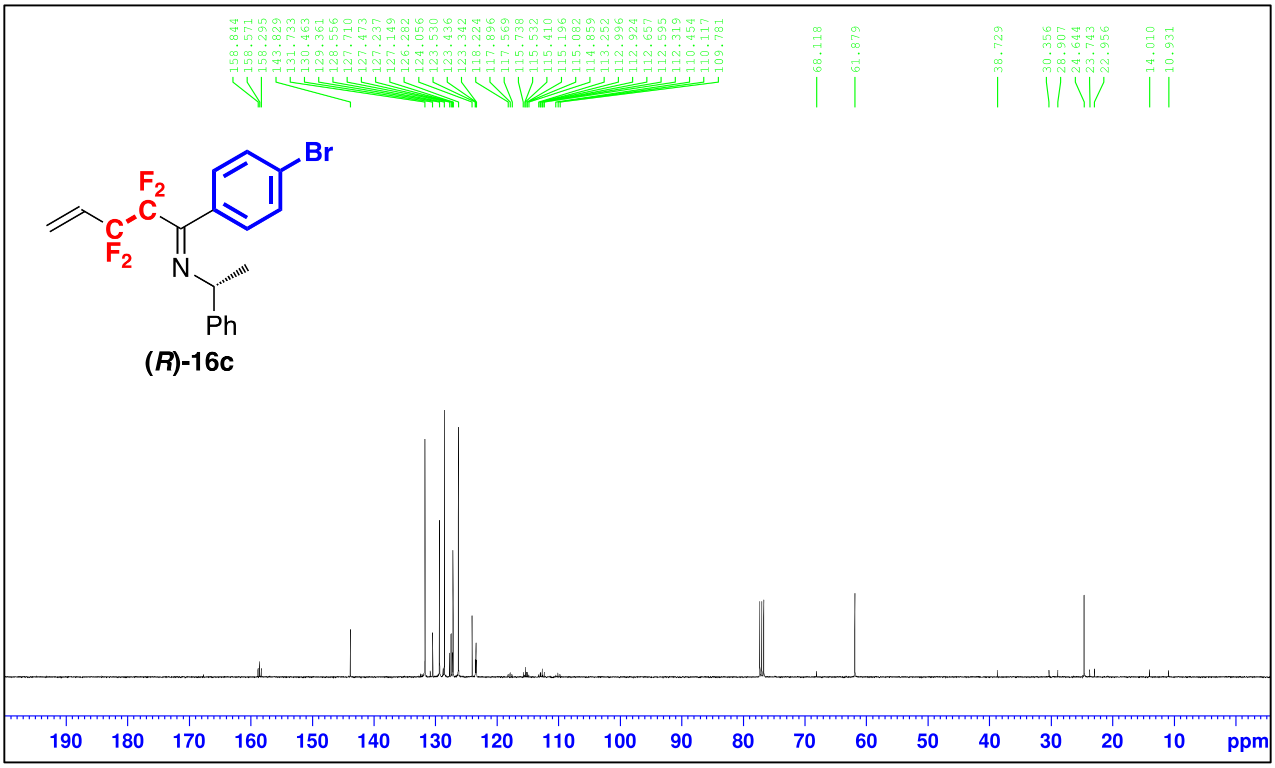

$^{19}F$ NMR Spectrum of (*R*)-*N*-(1-(4-bromophenyl)-2,2,3,3-tetrafluoropent-4-en-1-ylidene)-1-phenyethylamine ((*R*)-**16c**)

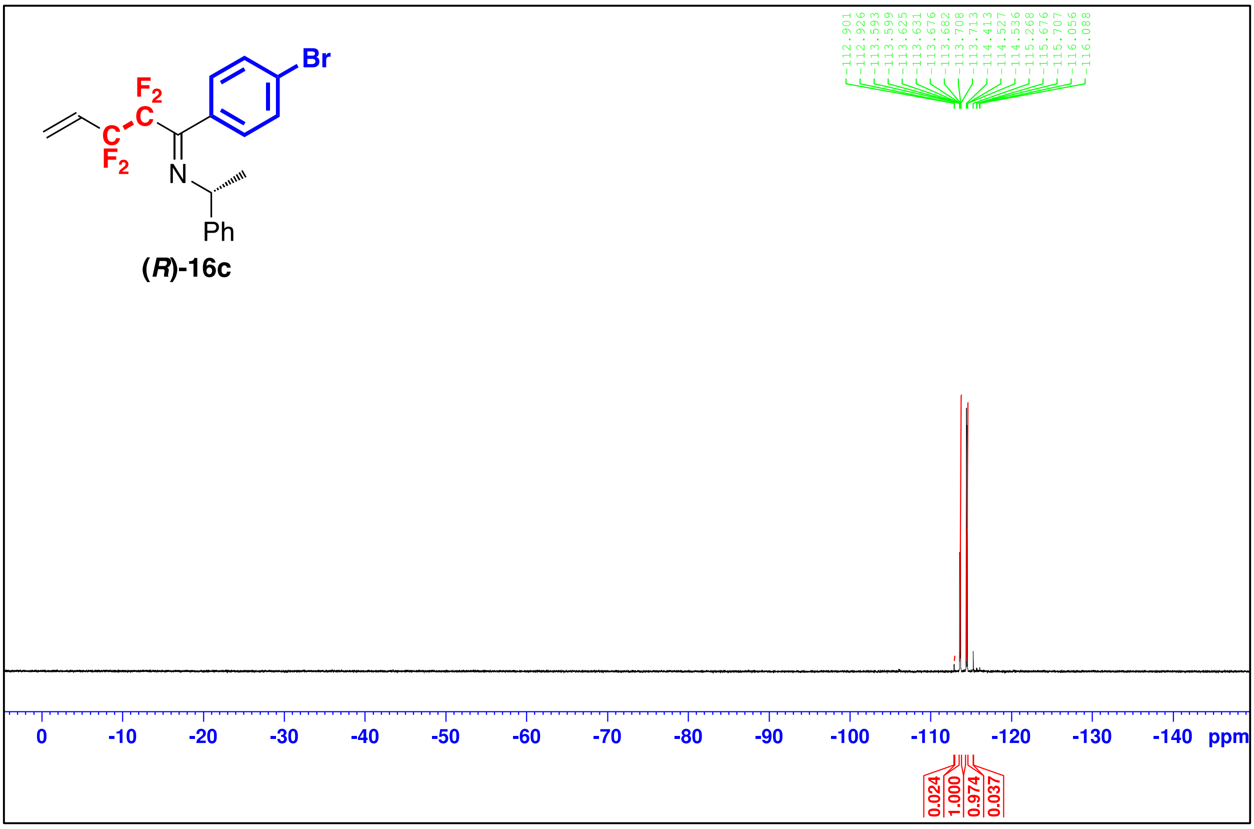

$^{1}$H NMR Spectrum of (*R*)-*N*-(2,2,3,3-tetrafluoro-1-(4-methoxyphenyl)pent-4-en-1-ylidene)-1-phenylethylamine ((*R*)-**16d**)

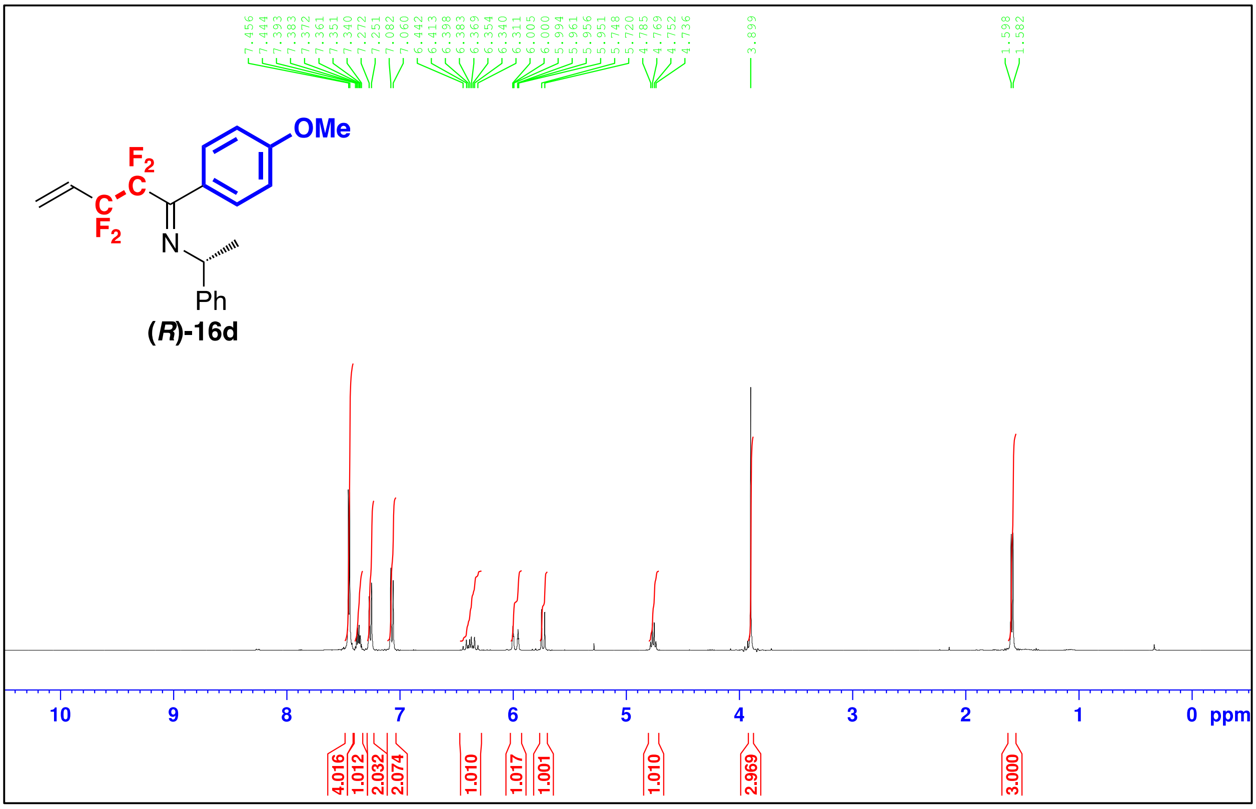


$^{13}$C NMR Spectrum of (*R*)-*N*-(2,2,3,3-tetrafluoro-1-(4-methoxyphenyl)pent-4-en-1-ylidene)-1-phenylethylamine ((*R*)-**16d**)

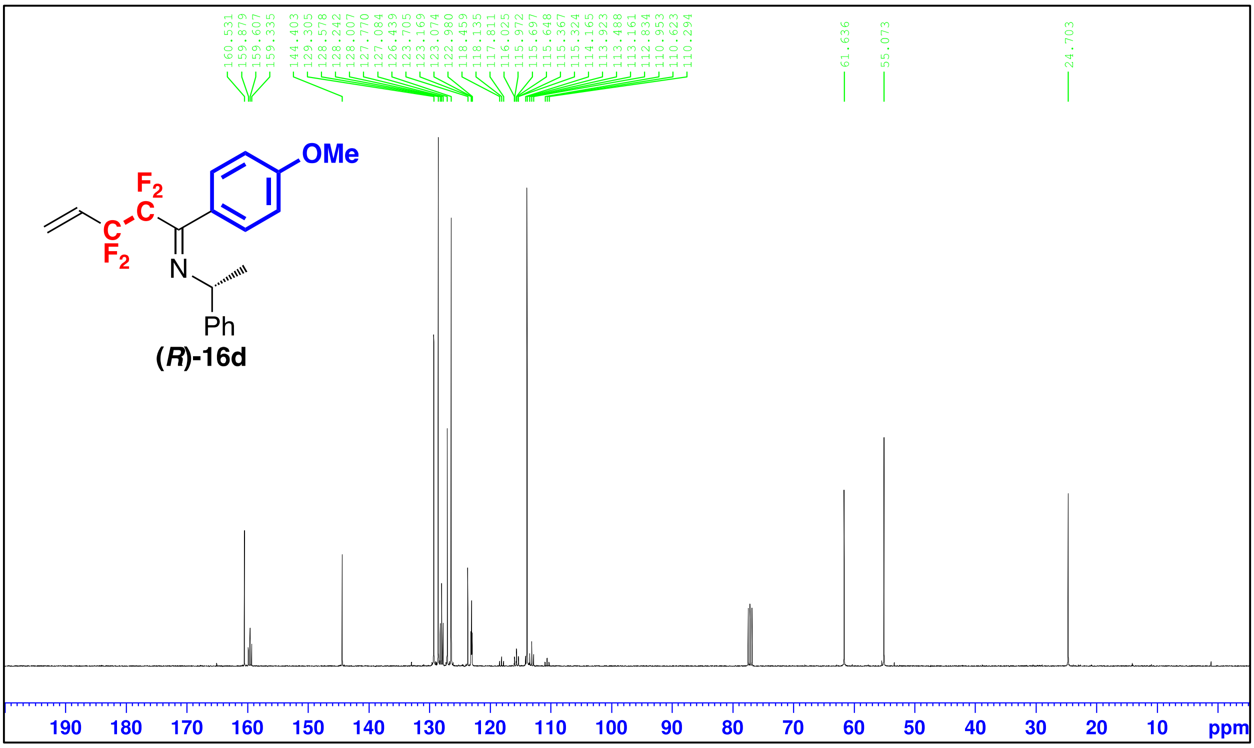

$^{19}$F NMR Spectrum of (*R*)-*N*-(2,2,3,3-tetrafluoro-1-(4-methoxyphenyl)pent-4-en-1-ylidene)-1-phenylethylamine ((*R*)-**16d**)

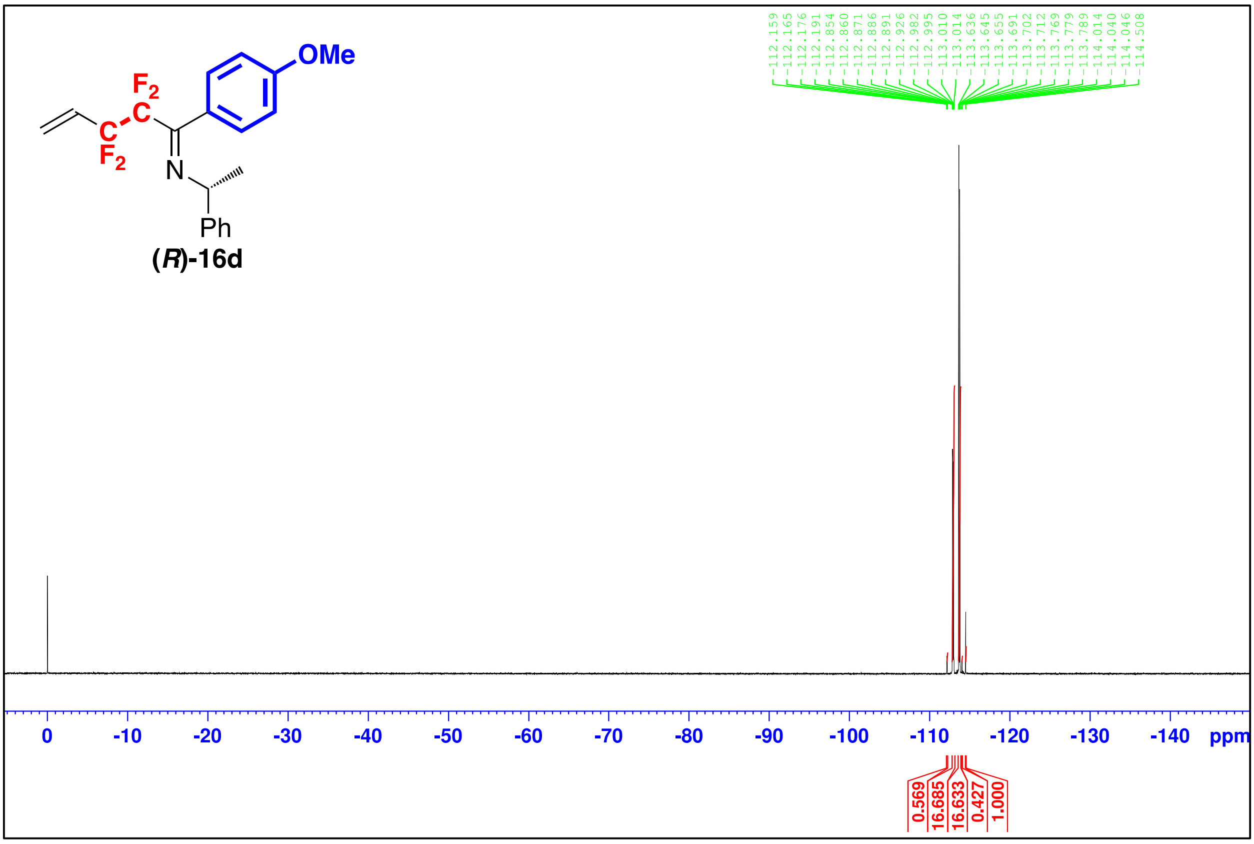

$^{1}$H NMR Spectrum of (*R*)-*N*-(2,2,3,3-tetrafluoro-1-(4-methylphenyl)pent-4-en-1-ylidene)-1-phenylethylamine ((*R*)-**16e**)

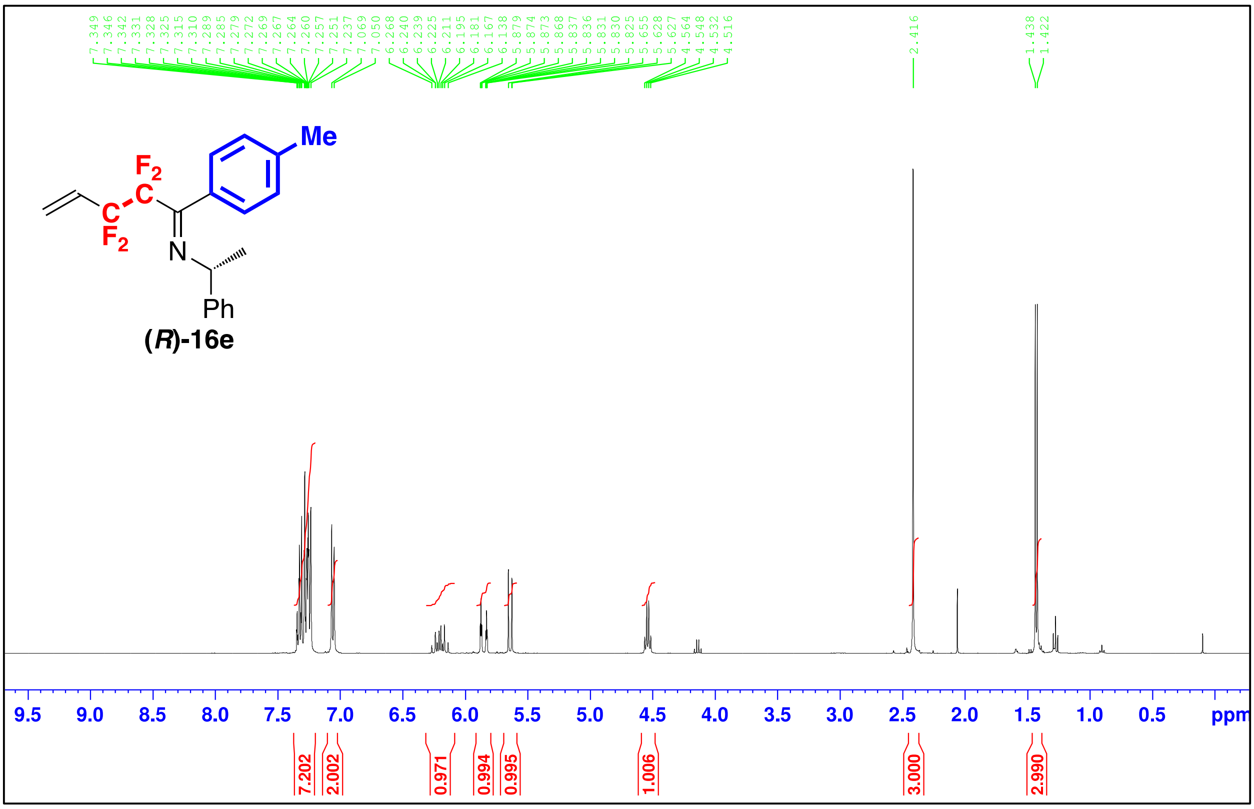

$^{13}$C NMR Spectrum of (*R*)-*N*-(2,2,3,3-tetrafluoro-1-(4-methylphenyl)pent-4-en-1-ylidene)-1-phenylethylamine ((*R*)-**16e**)

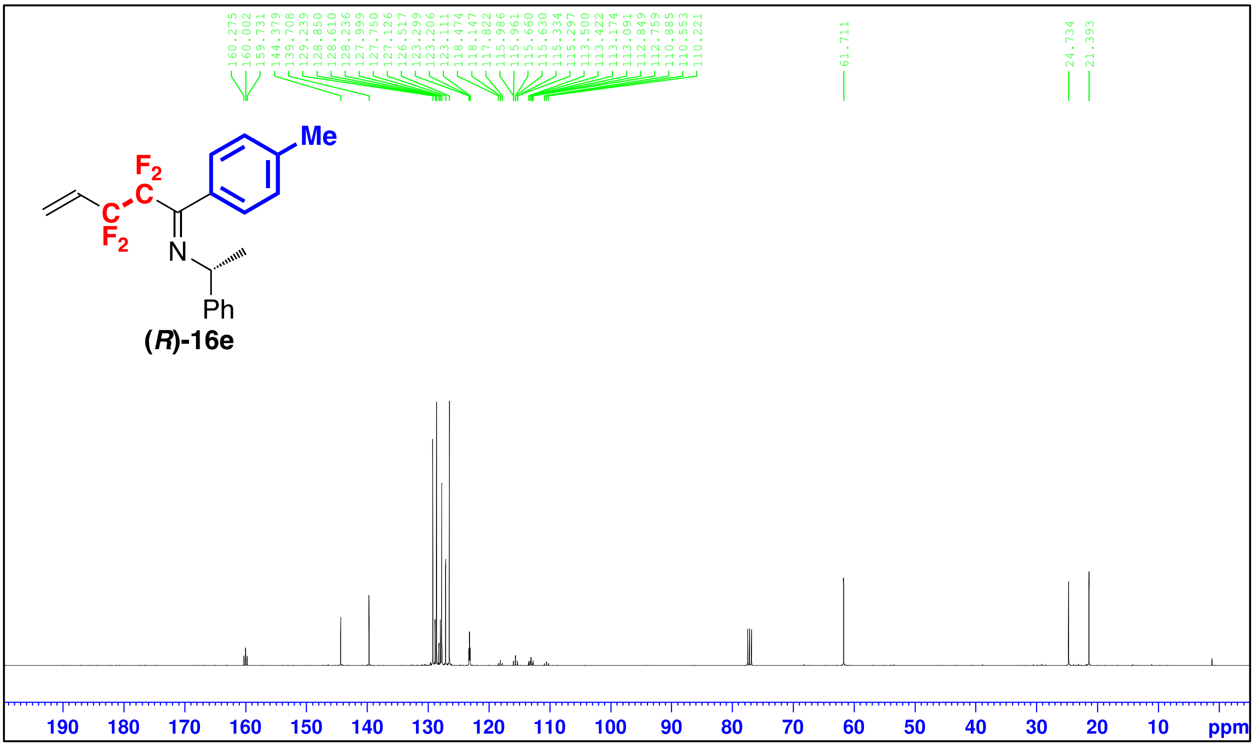

$^{19}$F NMR Spectrum of (*R*)-*N*-(2,2,3,3-tetrafluoro-1-(4-methylphenyl)pent-4-en-1-ylidene)-1-phenylethylamine ((*R*)-**16e**)

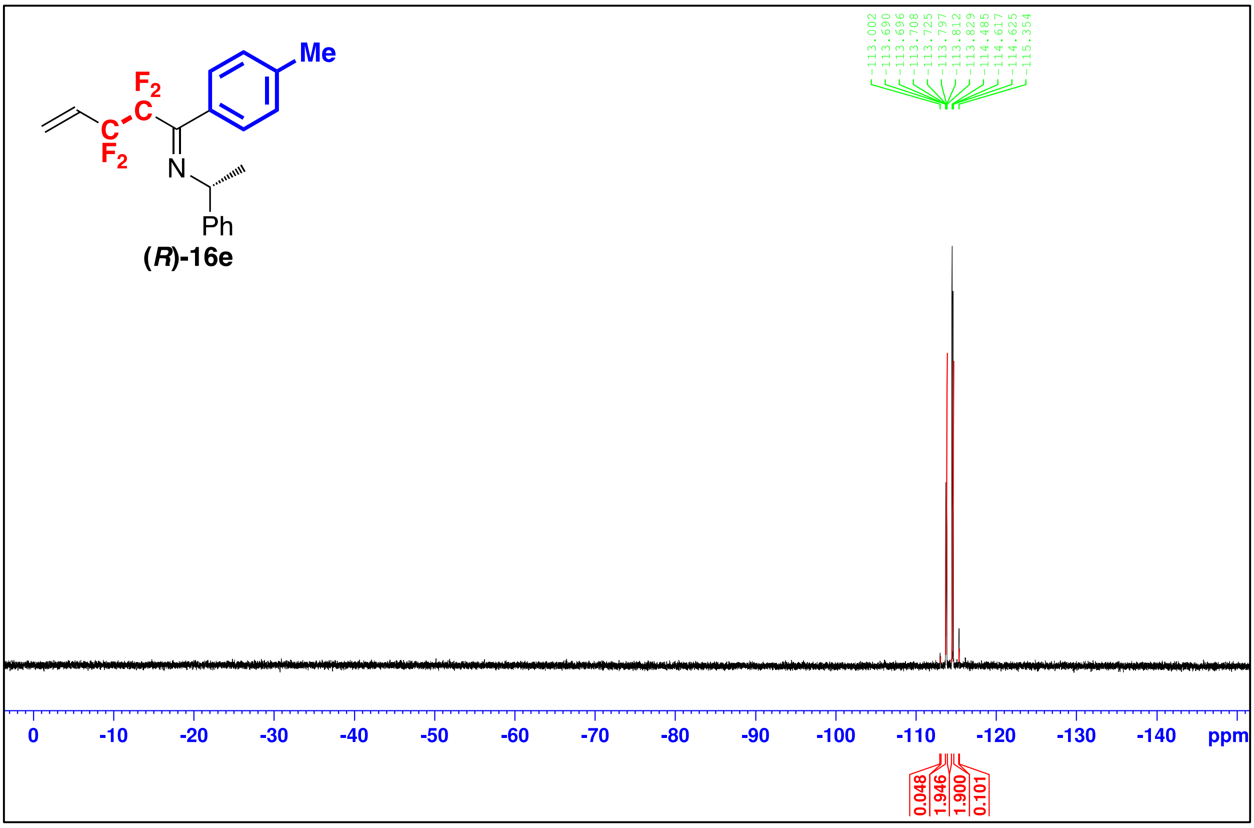

$^{1}$H NMR Spectrum of (*R*)-*N*-(2,2,3,3-tetrafluoro-1-(3-methylphenyl)pent-4-en-1-ylidene)-1-phenylethylamine ((*R*)-**16f**)

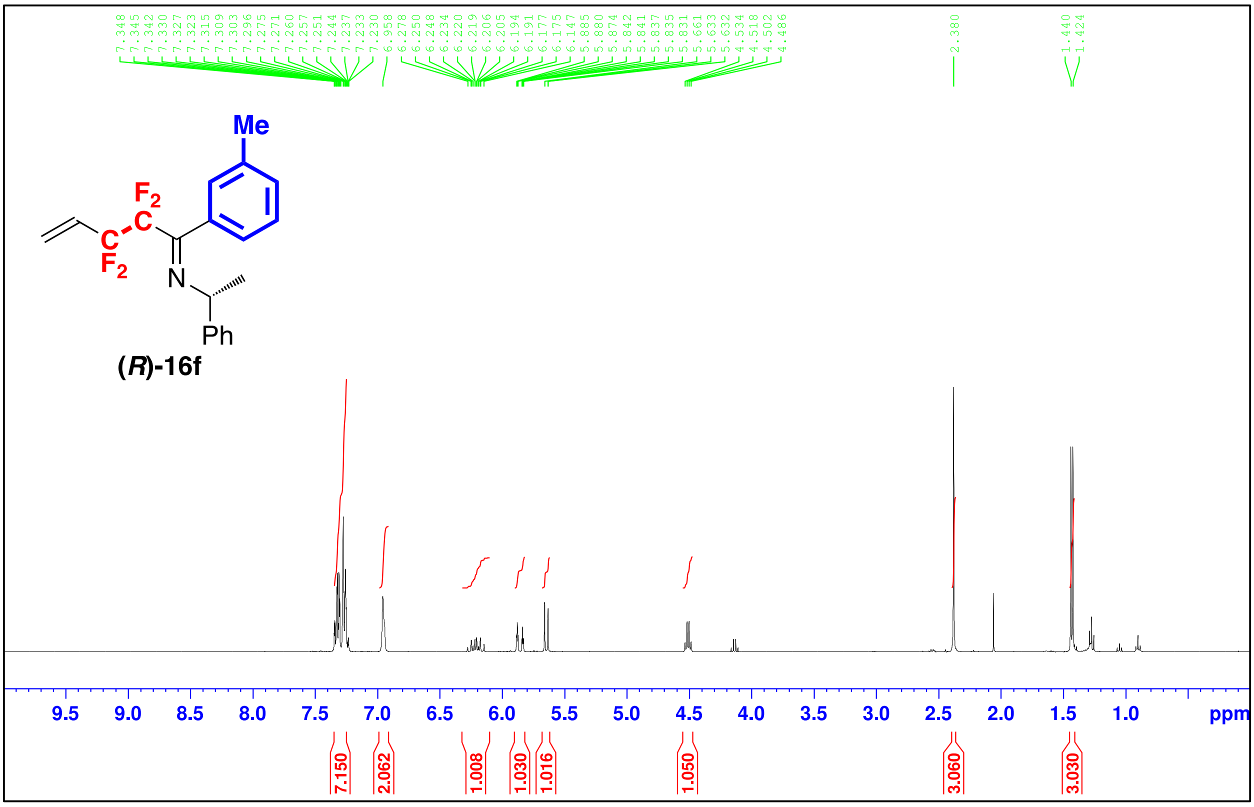


$^{13}$C NMR Spectrum of (*R*)-*N*-(2,2,3,3-tetrafluoro-1-(3-methylphenyl)pent-4-en-1-ylidene)-1-phenylethylamine ((*R*)-**16f**)

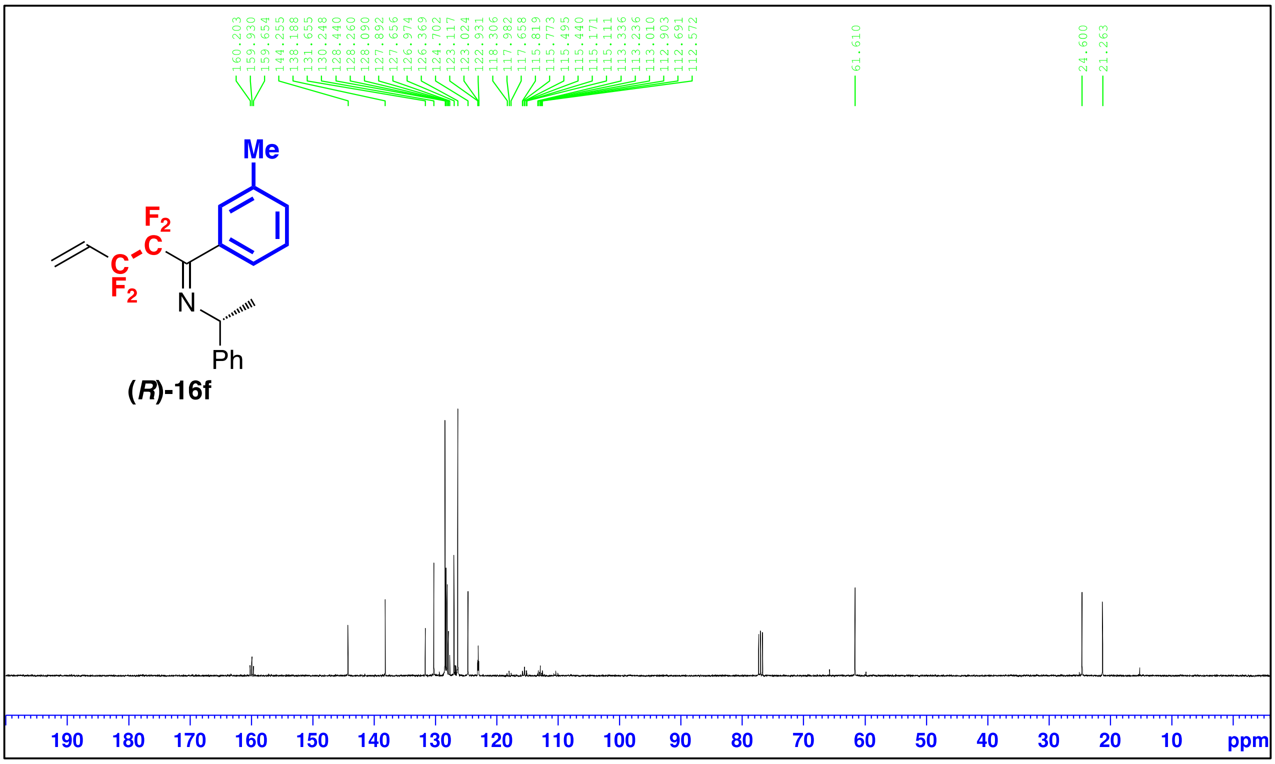

$^{19}$F NMR Spectrum of (*R*)-*N*-(2,2,3,3-tetrafluoro-1-(3-methylphenyl)pent-4-en-1-ylidene)-1-phenylethylamine ((*R*)-**16f**)

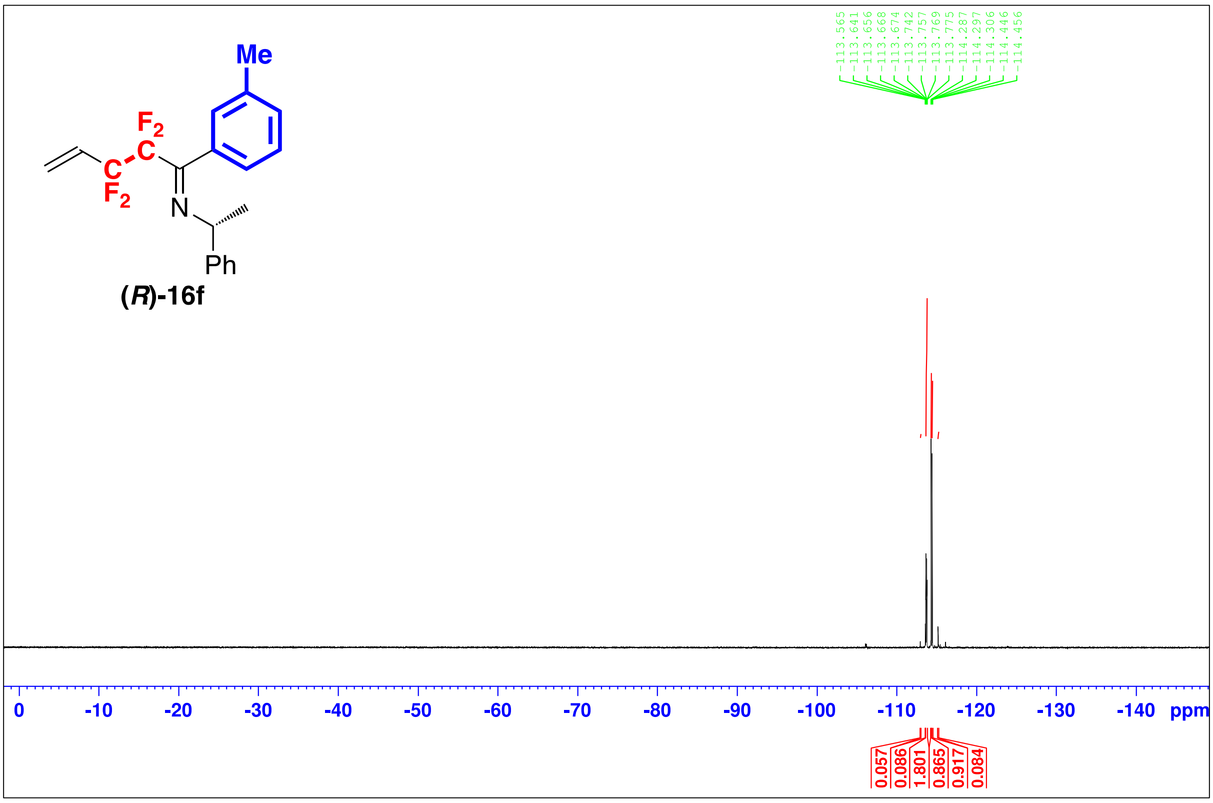

$^{1}H$ NMR Spectrum of (*R*)-*N*-(2,2,3,3-tetrafluoro-1-(2-methylphenyl)pent-4-en-1-ylidene)-1-phenylethylamine ((*R*)-**16g**)

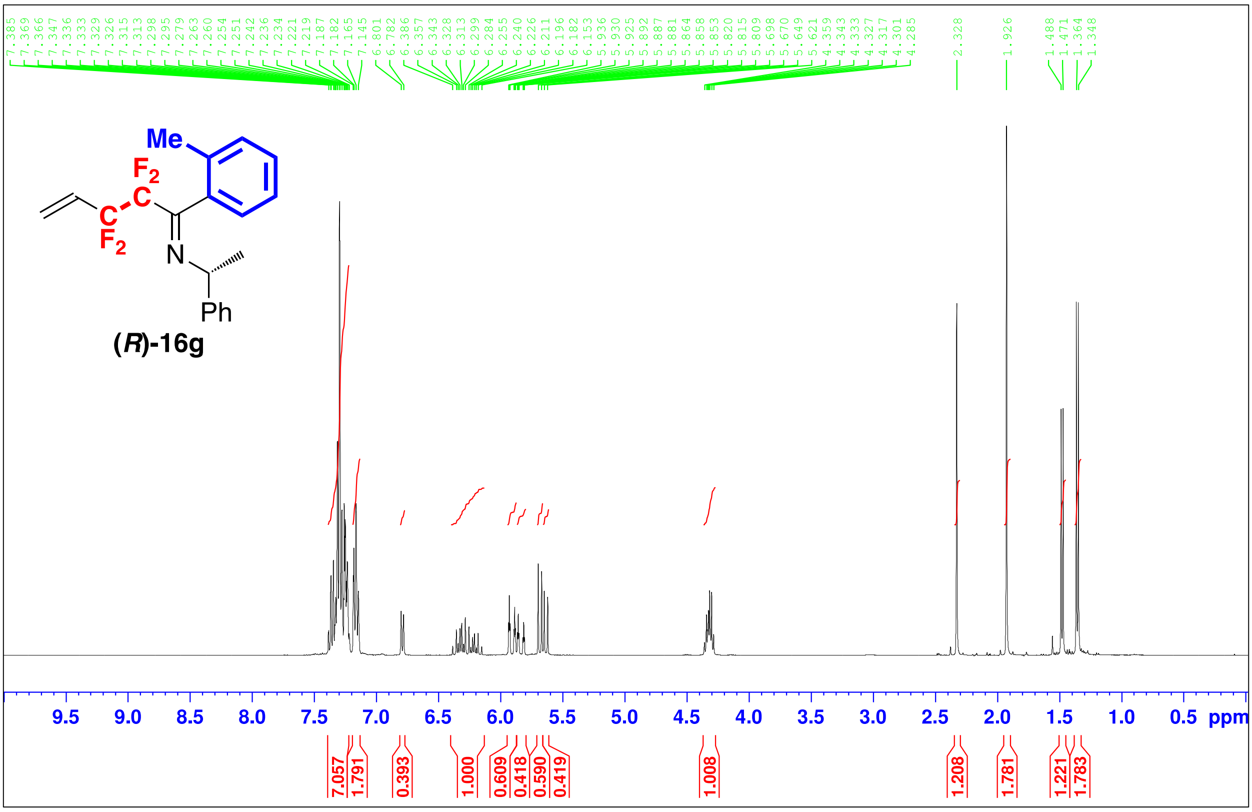


$^{13}C$ NMR Spectrum of (*R*)-*N*-(2,2,3,3-tetrafluoro-1-(2-methylphenyl)pent-4-en-1-ylidene)-1-phenylethylamine ((*R*)-**16g**))

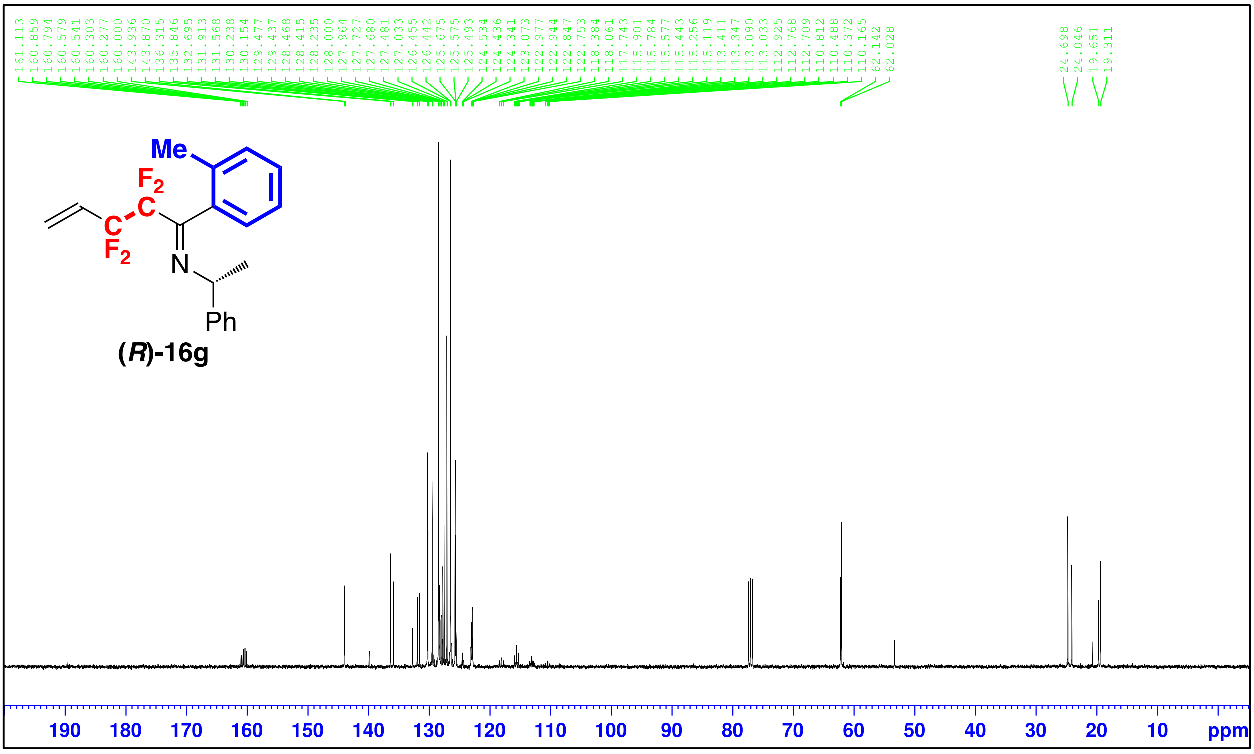

$^{19}$F NMR Spectrum of (*R*)-*N*-(2,2,3,3-tetrafluoro-1-(2-methylphenyl)pent-4-en-1-ylidene)-1-phenylethylamine ((*R*)-**16g**)

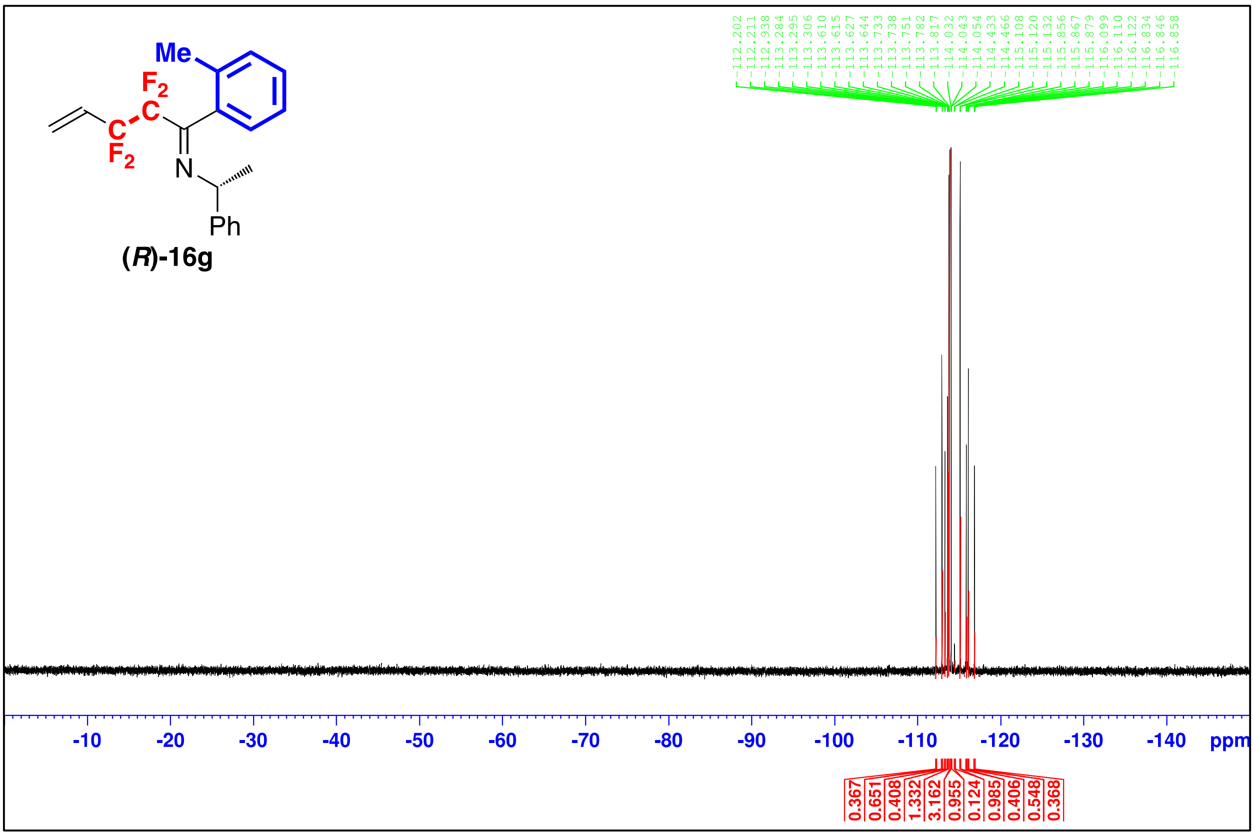

$^{1}$H NMR Spectrum of (*S*)-benzyl *N*-(2,2,3,3-tetrafluoro-1-phenylpent-4-en-1-yl)carbamate ((*S*)-**23a**)

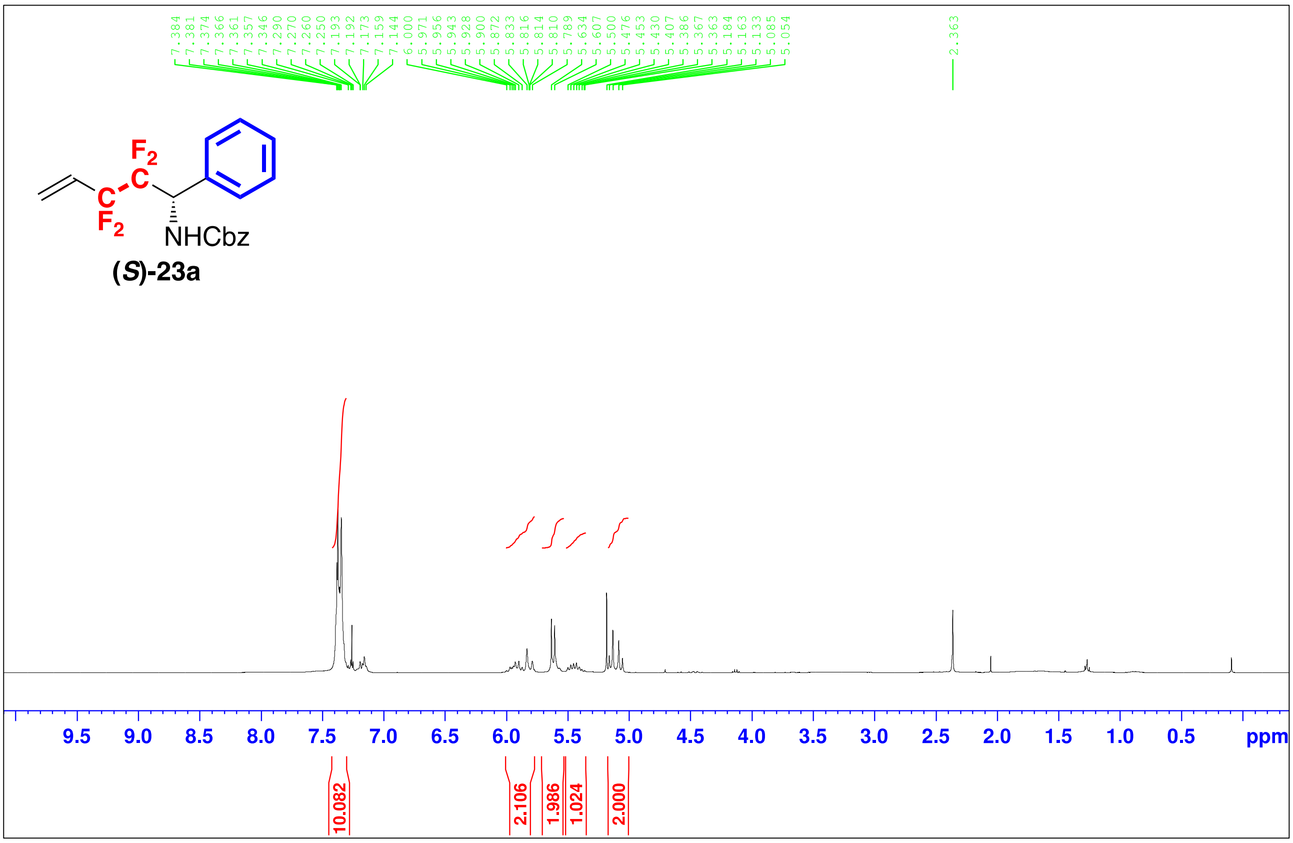


$^{13}$C NMR Spectrum of (*S*)-benzyl *N*-(2,2,3,3-tetrafluoro-1-phenylpent-4-en-1-yl)carbamate ((*S*)-**23a**)

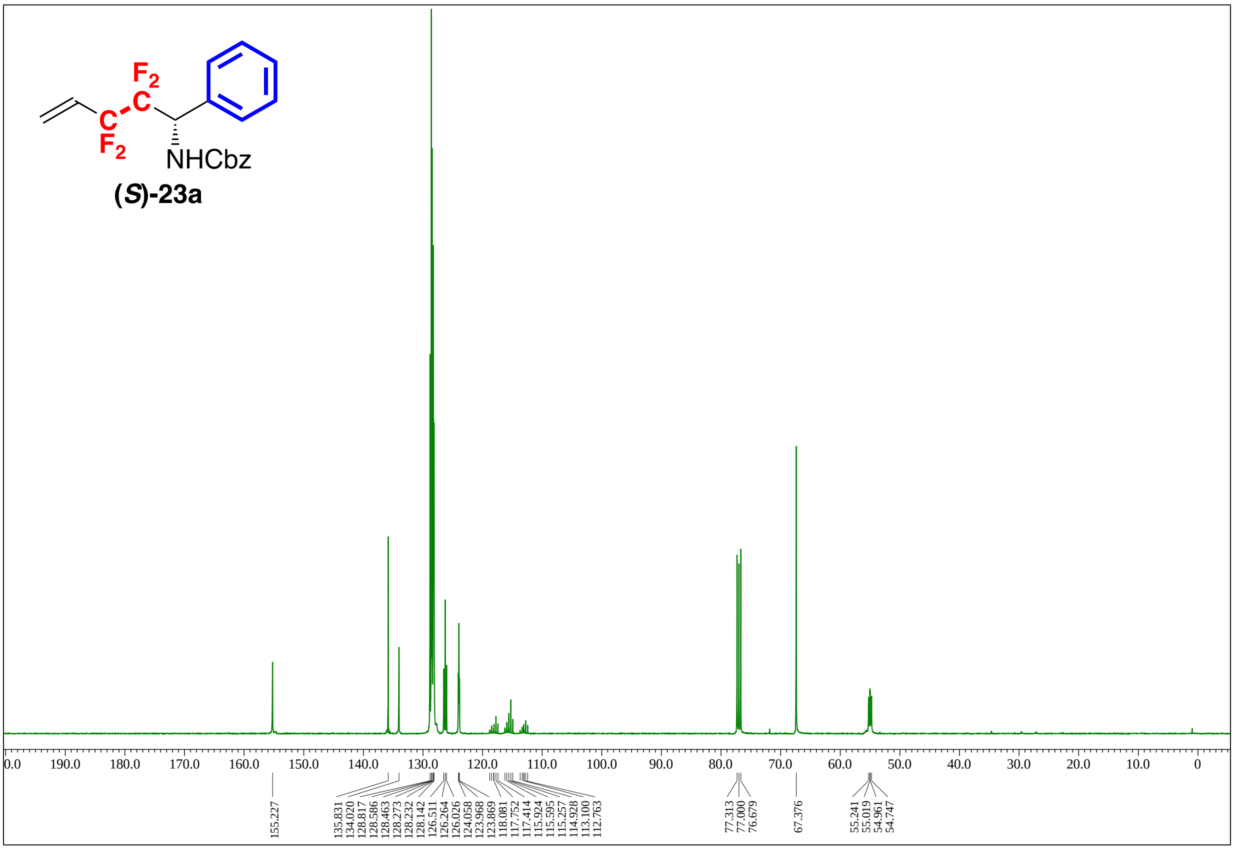

$^{19}$F NMR Spectrum of (*S*)-benzyl *N*-(2,2,3,3-tetrafluoro-1-phenylpent-4-en-1-yl)carbamate ((*S*)-**23a**)

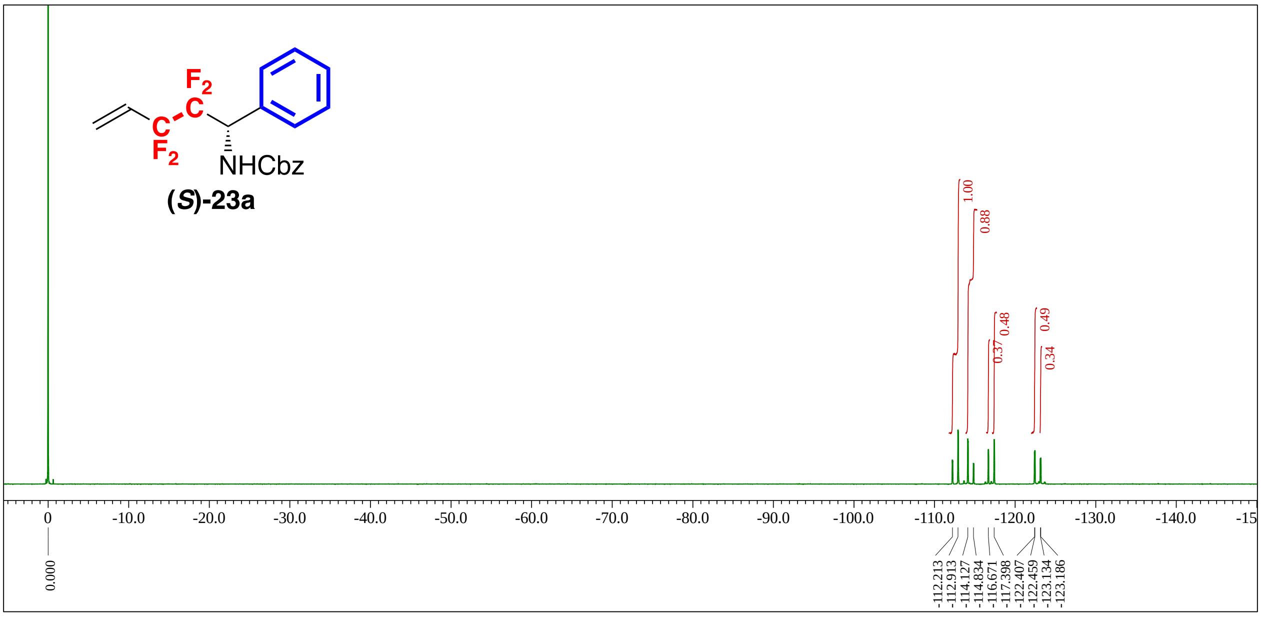

Chromatograph in HPLC for (*S*)-**23a**

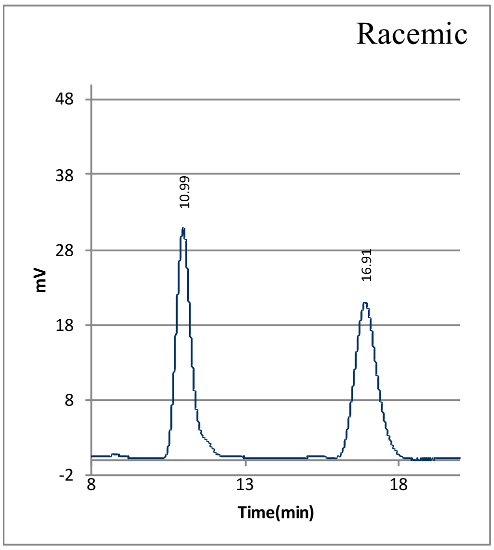

| No. | Rt | Area(%) |
|---|---|---|
| 1 | 10.99 | 50.019 |
| 2 | 16.91 | 49.981 |
| | | 100 |

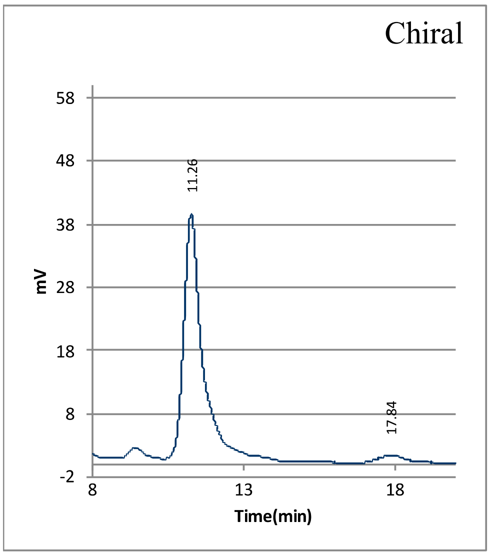

| No. | Rt | Area(%) |
|---|---|---|
| 1 | 11.26 | 96.085 |
| 2 | 17.84 | 3.915 |
| | | 100 |

$^{1}$H NMR Spectrum of (*S*)-benzyl *N*-(2,2,3,3-tetrafluoro-1-(4-chlorophenylpent-4-en-1-yl)carbamate ((*S*)-**23b**)

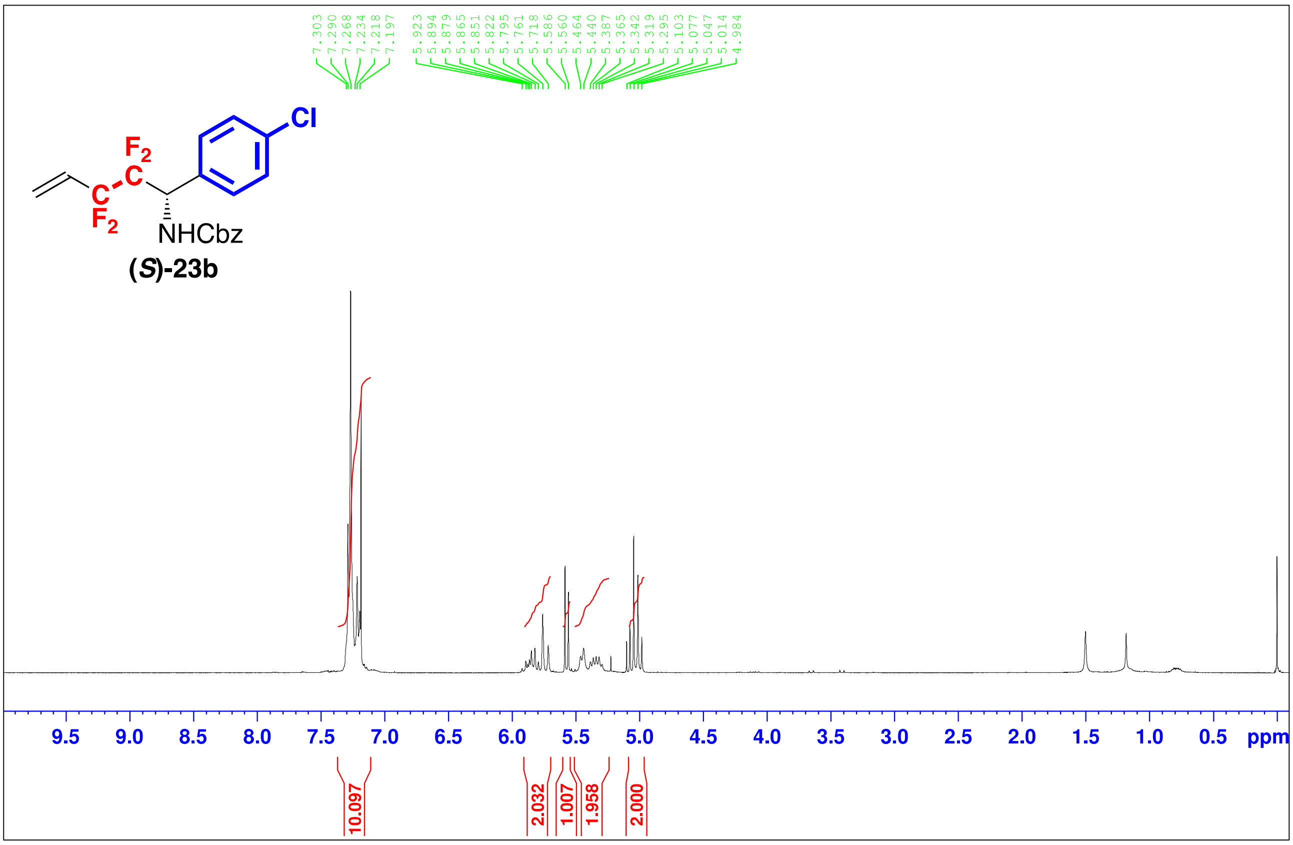

$^{13}$C NMR Spectrum of (*S*)-benzyl *N*-(2,2,3,3-tetrafluoro-1-(4-chlorophenylpent-4-en-1-yl)carbamate ((*S*)-**23b**)

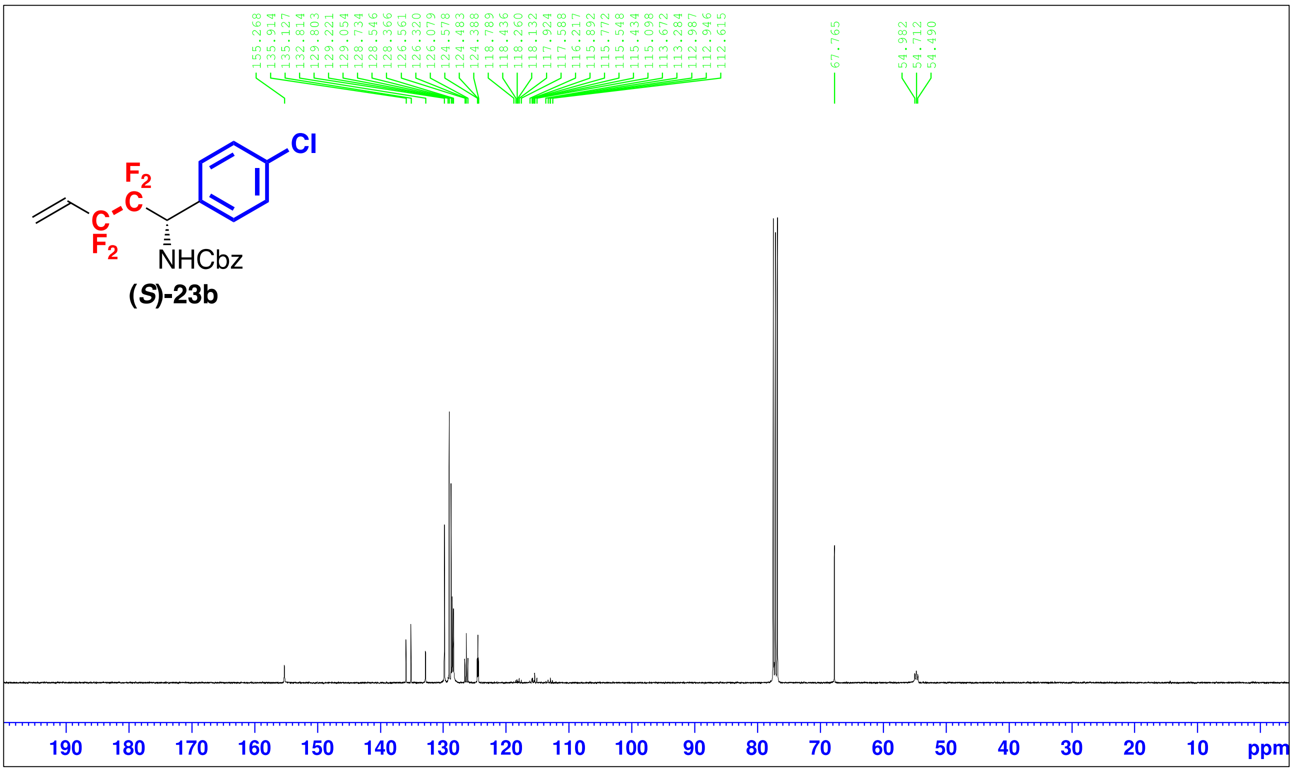

$^{19}$F NMR Spectrum of (*S*)-benzyl *N*-(2,2,3,3-tetrafluoro-1-(4-chlorophenylpent-4-en-1-yl)carbamate ((*S*)-**23b**)

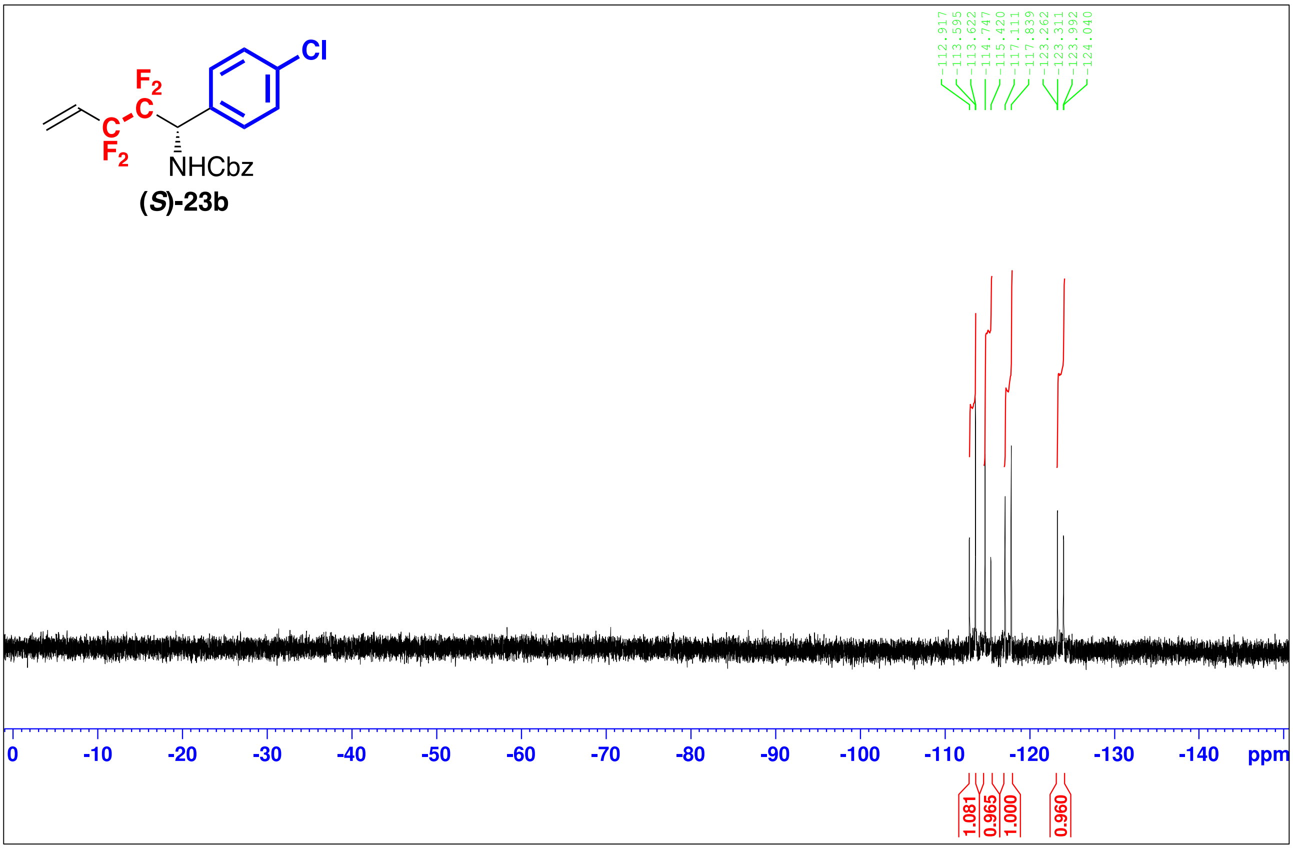


Chromatograph in HPLC for (*S*)-**23b**

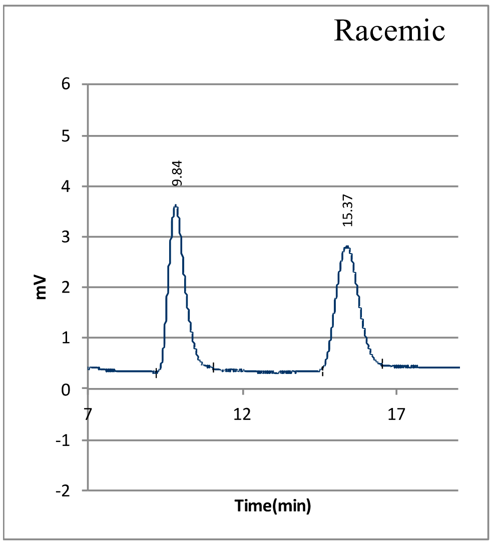


| No. | Rt | Area(%) |
|---|---|---|
| 1 | 9.84 | 49.966 |
| 2 | 15.37 | 50.034 |
| | | 100 |

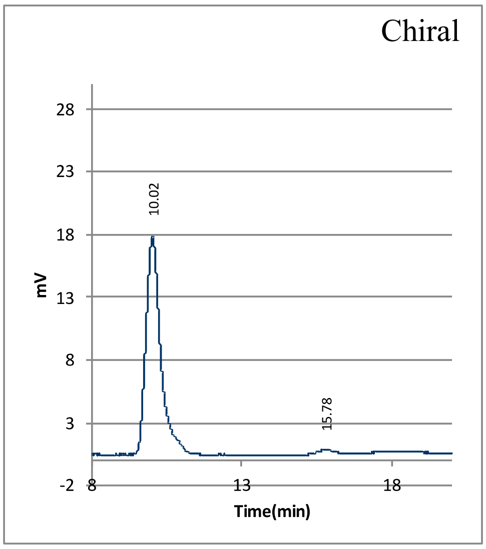


| No. | Rt | Area(%) |
|---|---|---|
| 1 | 10.02 | 99.054 |
| 2 | 15.78 | 0.946 |
| | | 100 |

$^{1}$H NMR Spectrum of (*S*)-benzyl *N*-(2,2,3,3-tetrafluoro-1-(4-bromophenylpent-4-en-1-yl)carbamate ((*S*)-**23c**)

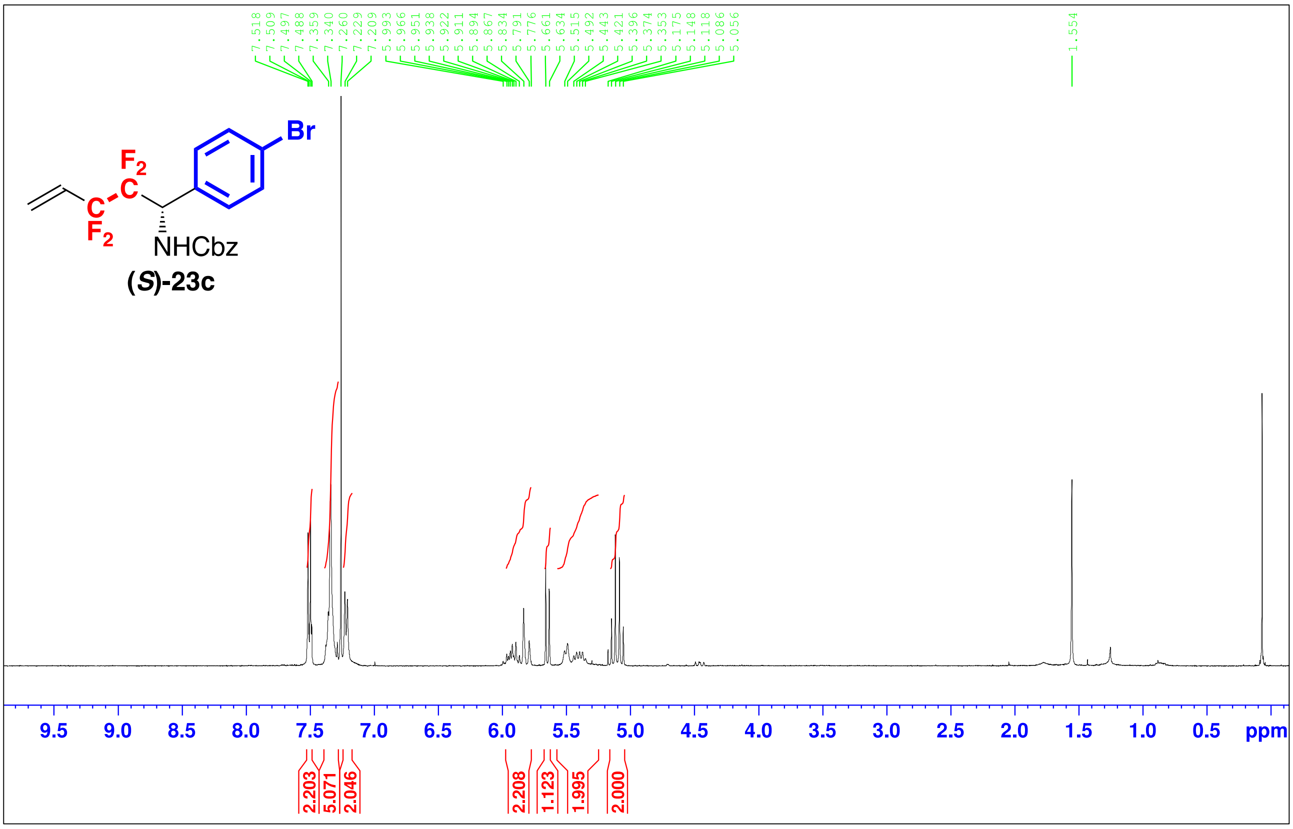


$^{13}$C NMR Spectrum of (*S*)-benzyl *N*-(2,2,3,3-tetrafluoro-1-(4-bromophenylpent-4-en-1-yl)carbamate ((*S*)-**23c**)

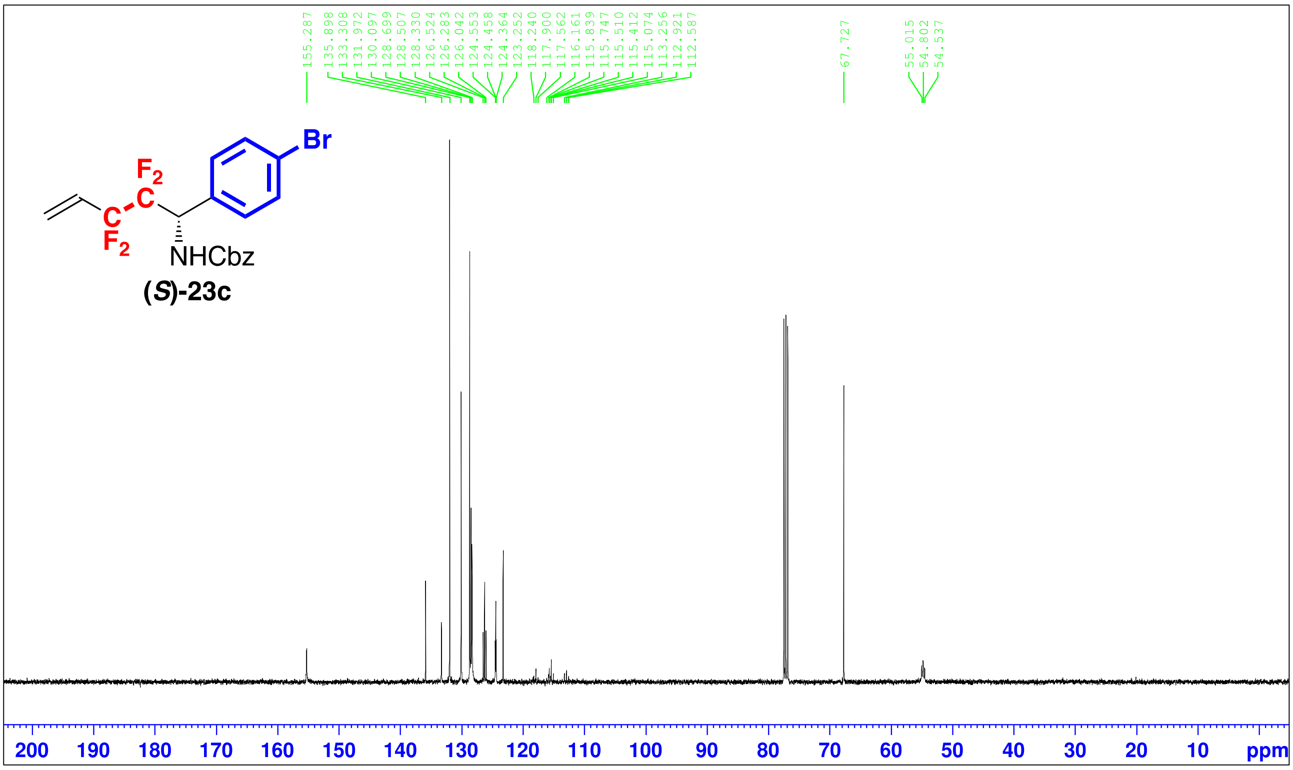

$^{19}F$ NMR Spectrum of (*S*)-benzyl *N*-(2,2,3,3-tetrafluoro-1-(4-bromophenylpent-4-en-1-yl)carbamate ((*S*)-**23c**)

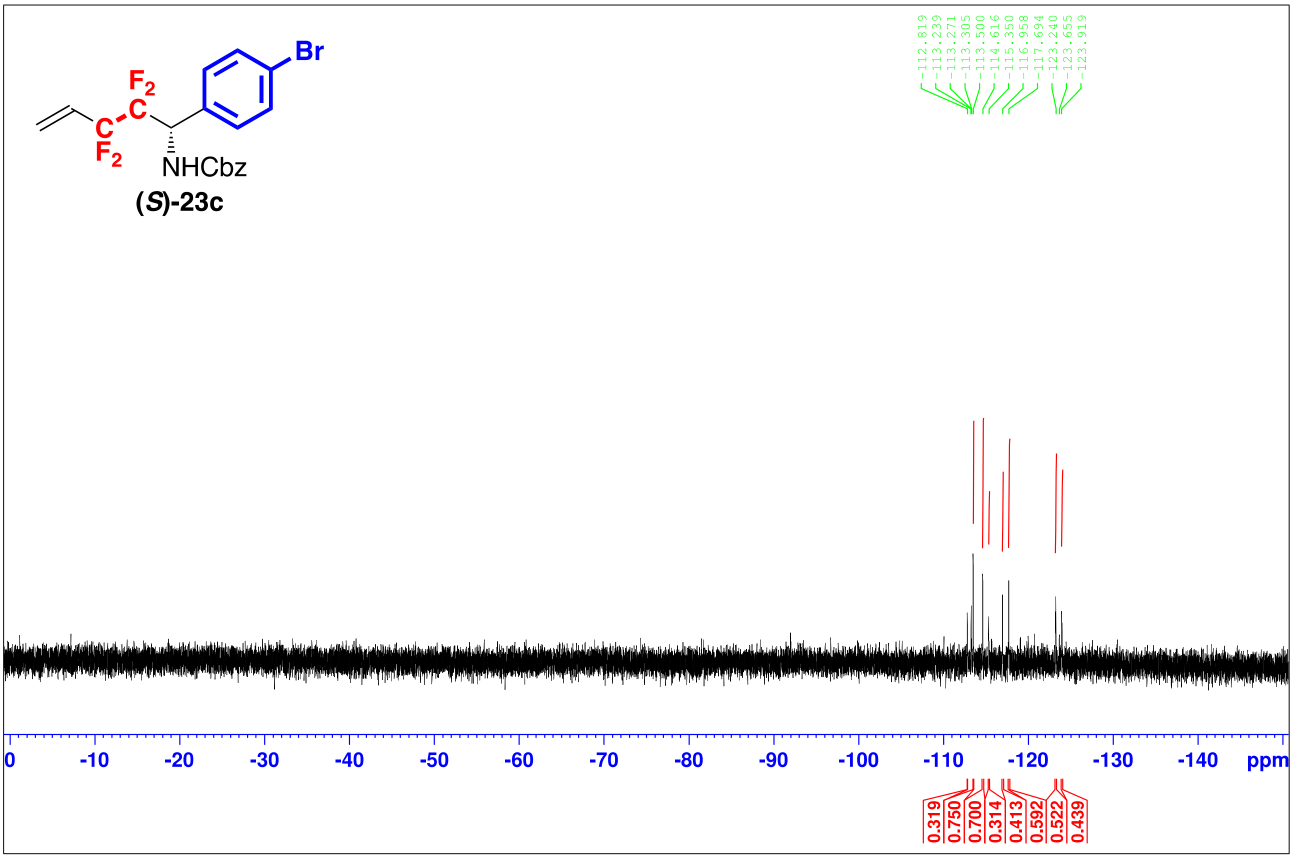


Chromatograph in HPLC for (*S*)-**23c**

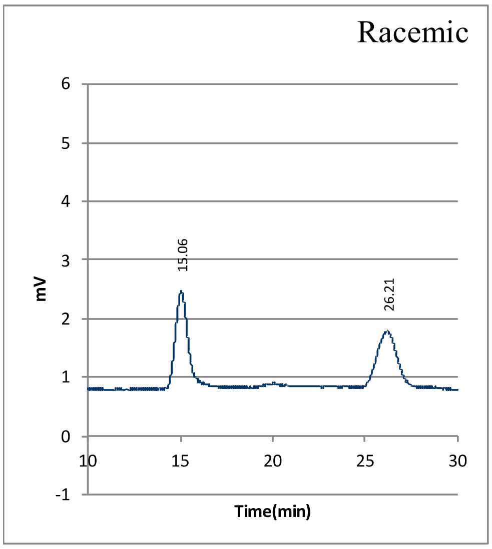


| No. | Rt | Area(%) |
|---|---|---|
| 1 | 15.06 | 49.901 |
| 2 | 26.21 | 50.099 |
| | | 100 |

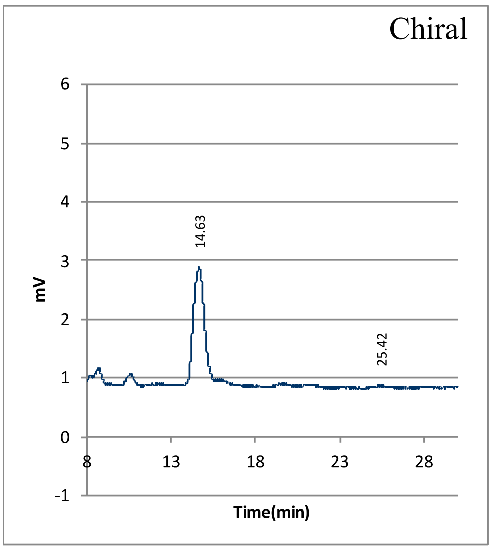


| No. | Rt | Area(%) |
|---|---|---|
| 1 | 14.63 | 98.824 |
| 2 | 25.42 | 1.176 |
| | | 100 |

$^{1}$H NMR Spectrum of (*S*)-benzyl *N*-(2,2,3,3-tetrafluoro-1-(4-methoxyphenylpent-4-en-1-yl)carbamate ((*S*)-**23d**)

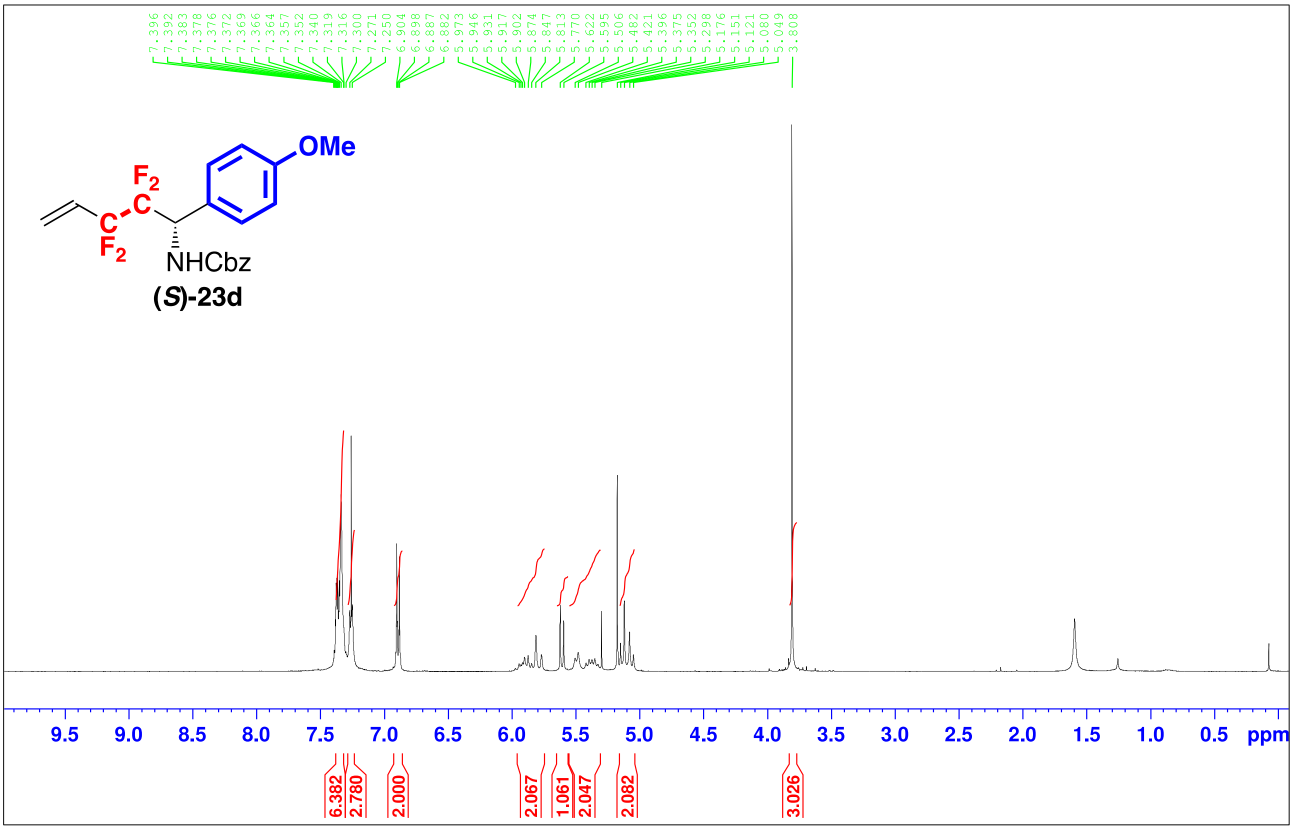


$^{13}$C NMR Spectrum of (*S*)-benzyl *N*-(2,2,3,3-tetrafluoro-1-(4-methoxyphenylpent-4-en-1-yl)carbamate ((*S*)-**23d**))

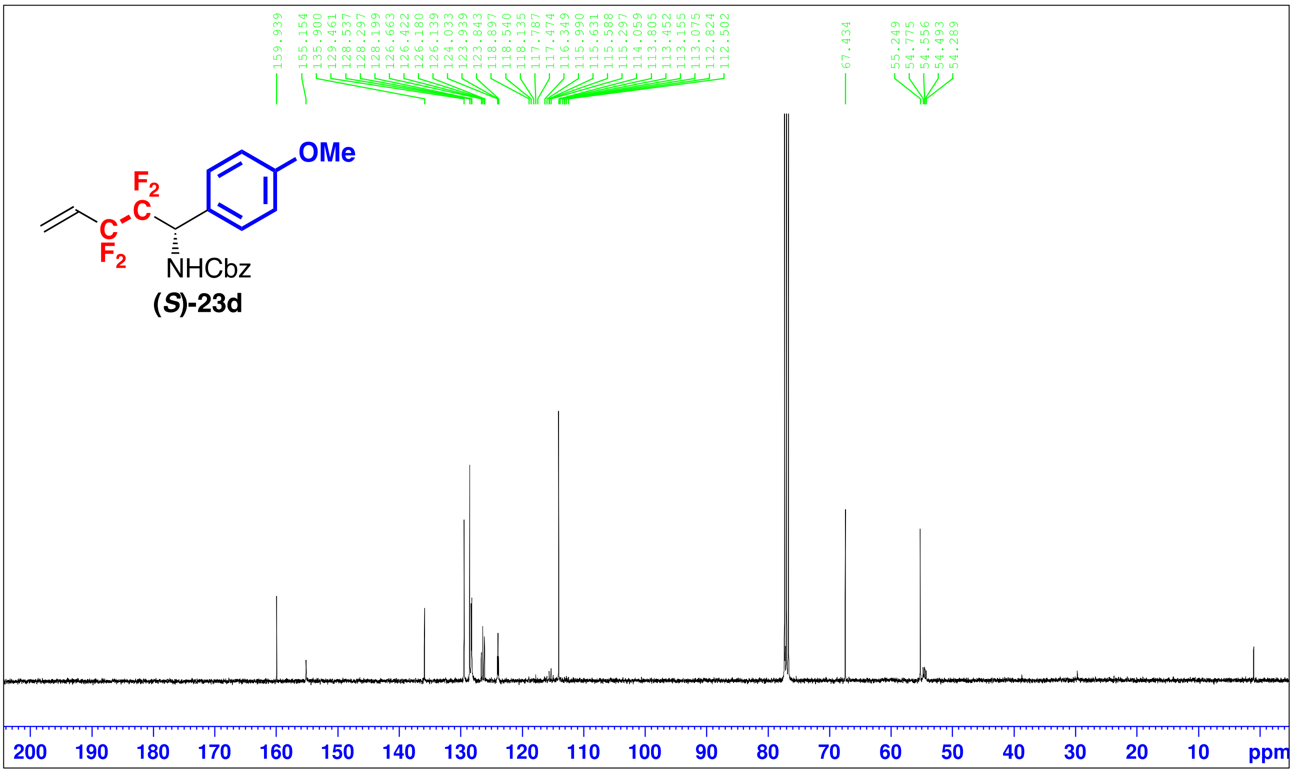

$^{19}$F NMR Spectrum of (*S*)-benzyl *N*-(2,2,3,3-tetrafluoro-1-(4-methoxyphenylpent-4-en-1-yl)carbamate ((*S*)-**23d**)

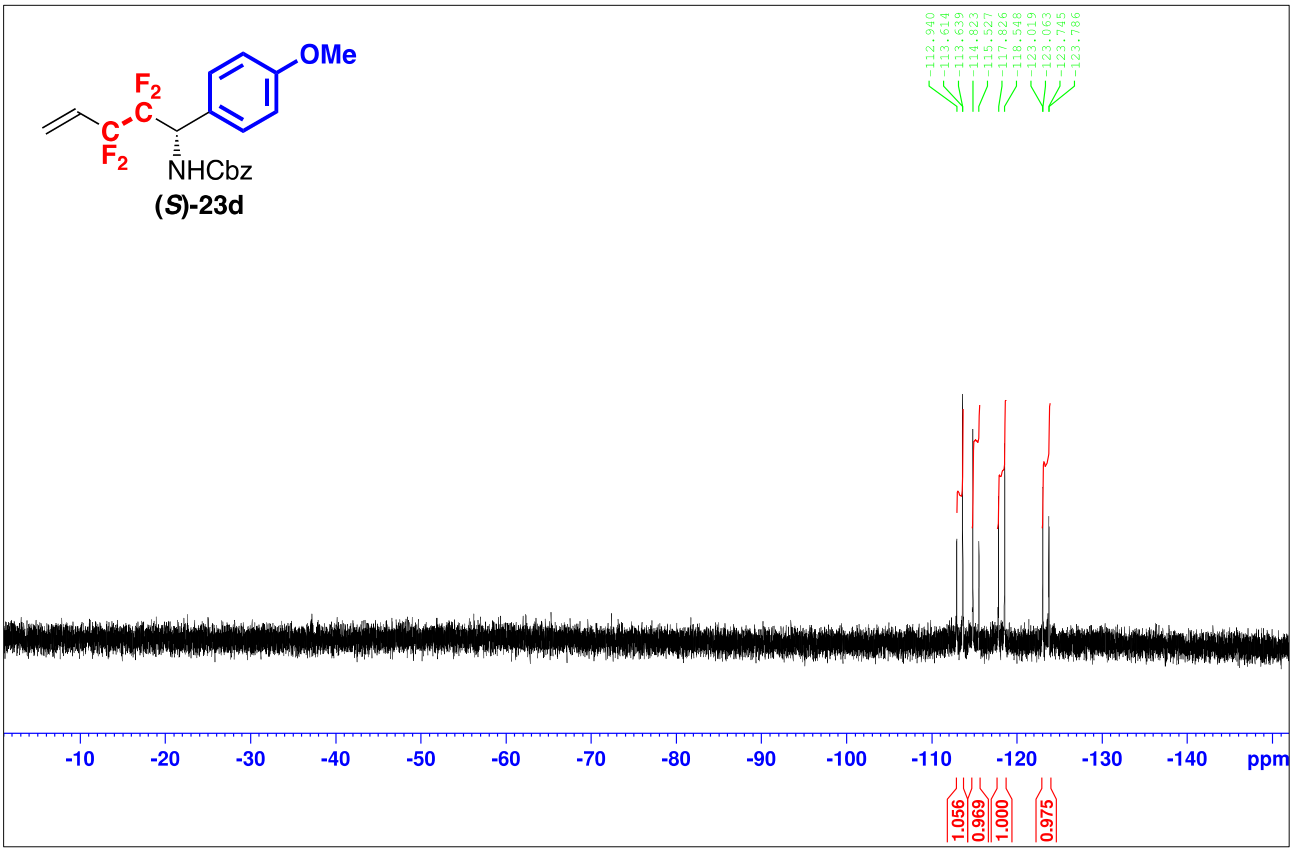


Chromatograph in HPLC for (*S*)-**23d**

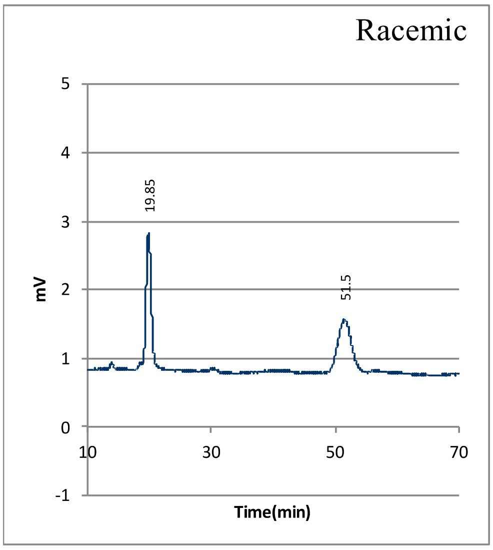


| No. | Rt | Area(%) |
|---|---|---|
| 1 | 19.85 | 50.086 |
| 2 | 51.5 | 49.914 |
| | | 100 |

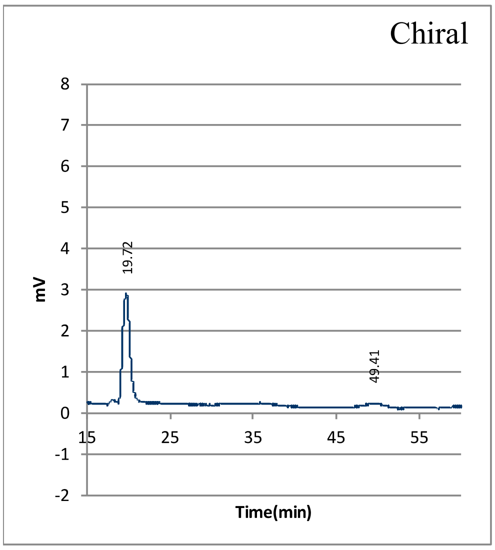


| No. | Rt | Area(%) |
|---|---|---|
| 1 | 19.72 | 95.216 |
| 2 | 49.41 | 4.784 |
| | | 100 |

$^{1}$H NMR Spectrum of (*S*)-benzyl *N*-(2,2,3,3-tetrafluoro-1-(4-methylphenylpent-4-en-1-yl)carbamate ((*S*)-**23e**)

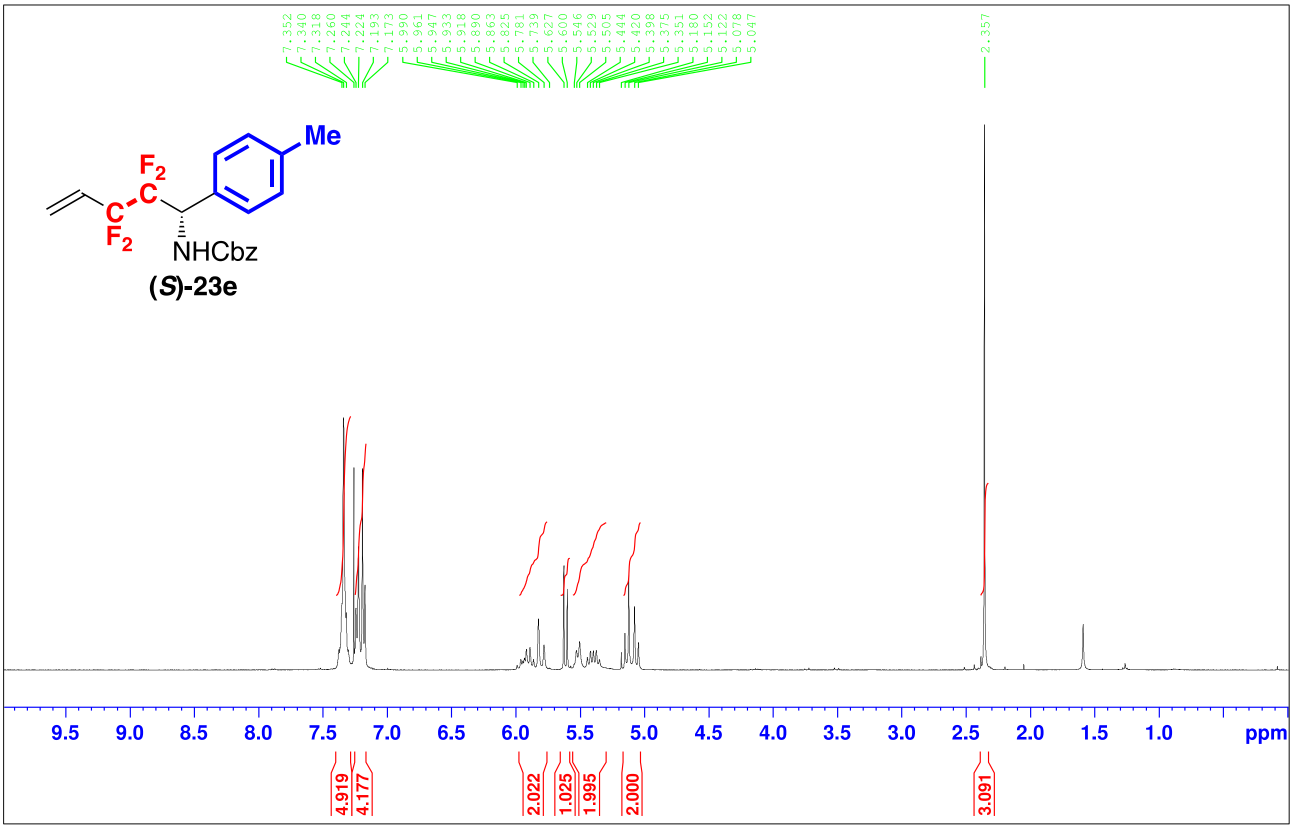


$^{13}$C NMR Spectrum of (*S*)-benzyl *N*-(2,2,3,3-tetrafluoro-1-(4-methylphenylpent-4-en-1-yl)carbamate ((*S*)-**23e**)

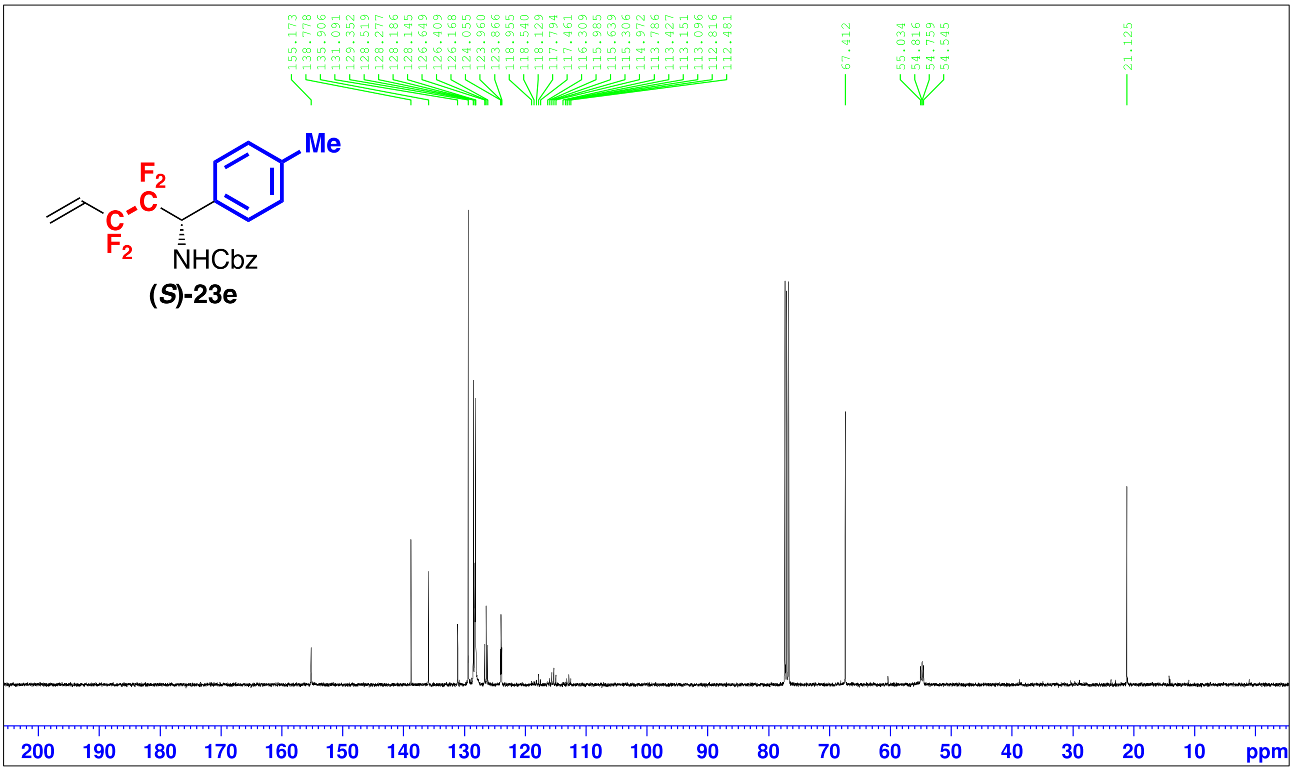

$^{19}$F NMR Spectrum of (*S*)-benzyl *N*-(2,2,3,3-tetrafluoro-1-(4-methylphenylpent-4-en-1-yl)carbamate ((*S*)-**23e**)

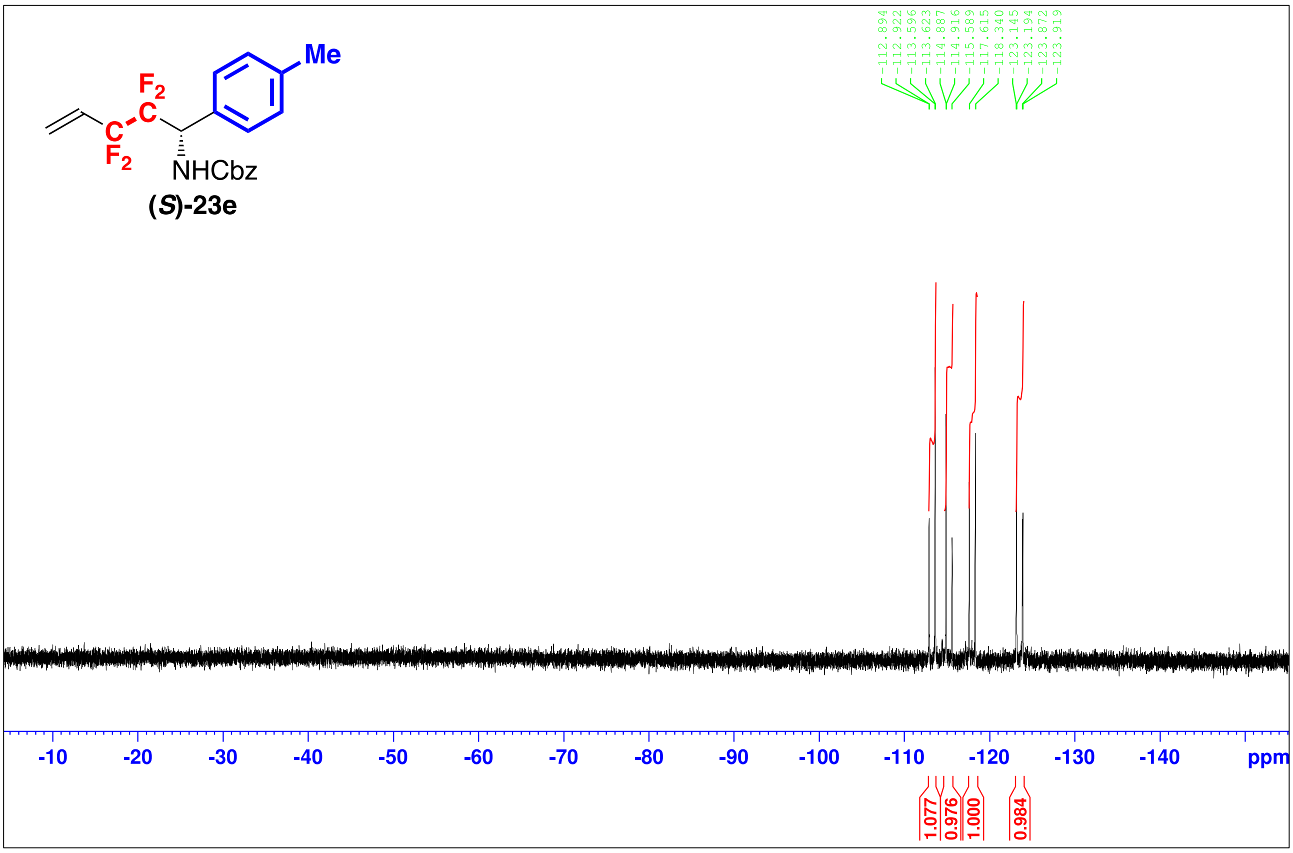


Chromatograph in HPLC for (*S*)-**23e**

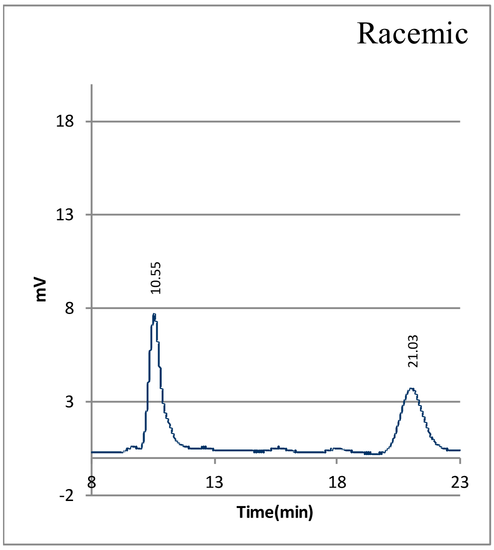


| No. | Rt | Area(%) |
|---|---|---|
| 1 | 10.55 | 49.92 |
| 2 | 21.03 | 50.08 |
| | | 100 |

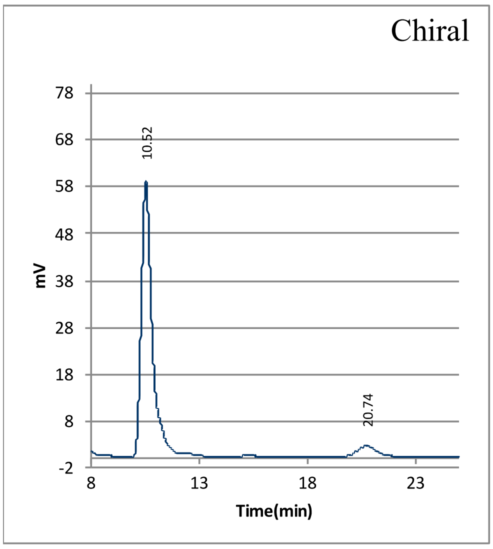


| No. | Rt | Area(%) |
|---|---|---|
| 1 | 10.52 | 95.043 |
| 2 | 20.74 | 4.957 |
| | | 100 |

$^{1}$H NMR Spectrum of (*S*)-benzyl *N*-(2,2,3,3-tetrafluoro-1-(3-methylphenylpent-4-en-1-yl)carbamate ((*S*)-**23f**)

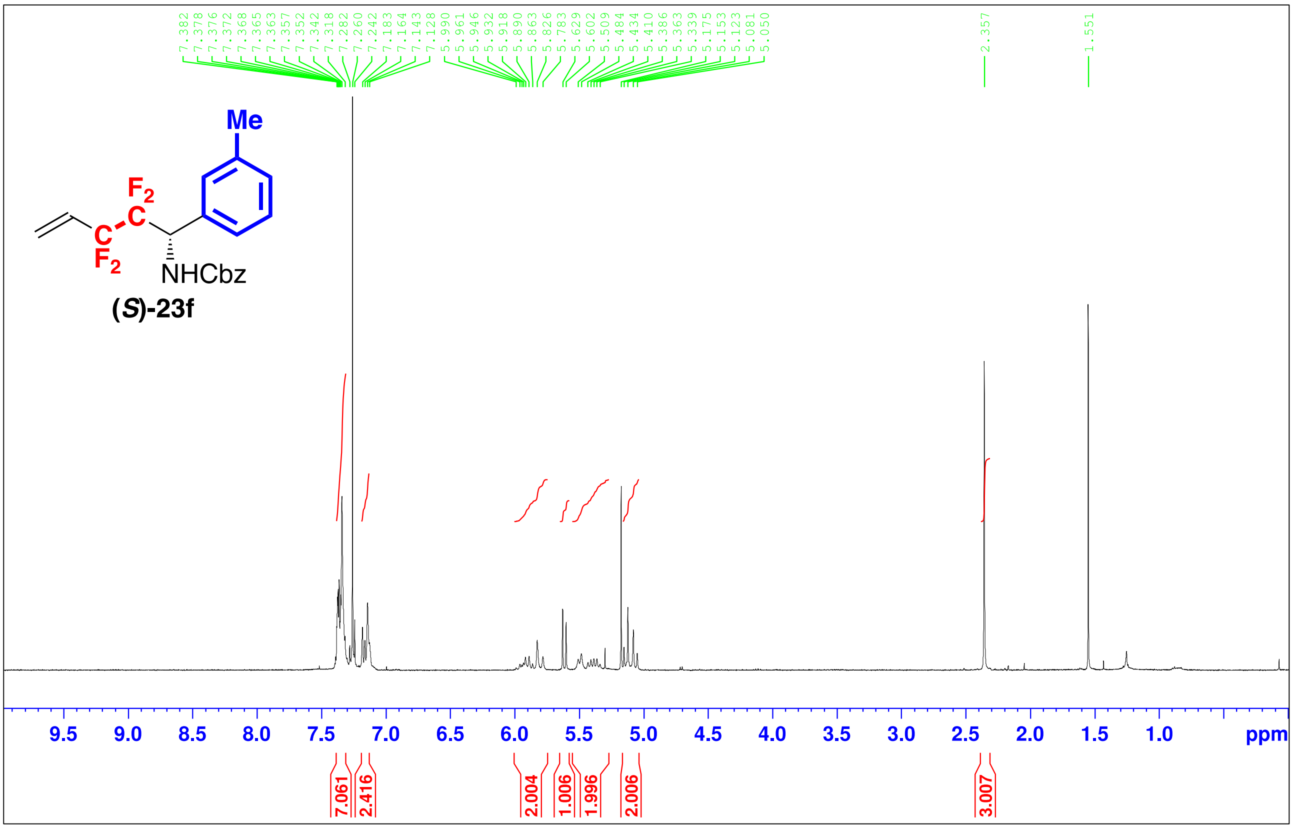


$^{13}$C NMR Spectrum of (*S*)-benzyl *N*-(2,2,3,3-tetrafluoro-1-(3-methylphenylpent-4-en-1-yl)carbamate ((*S*)-**23f**)

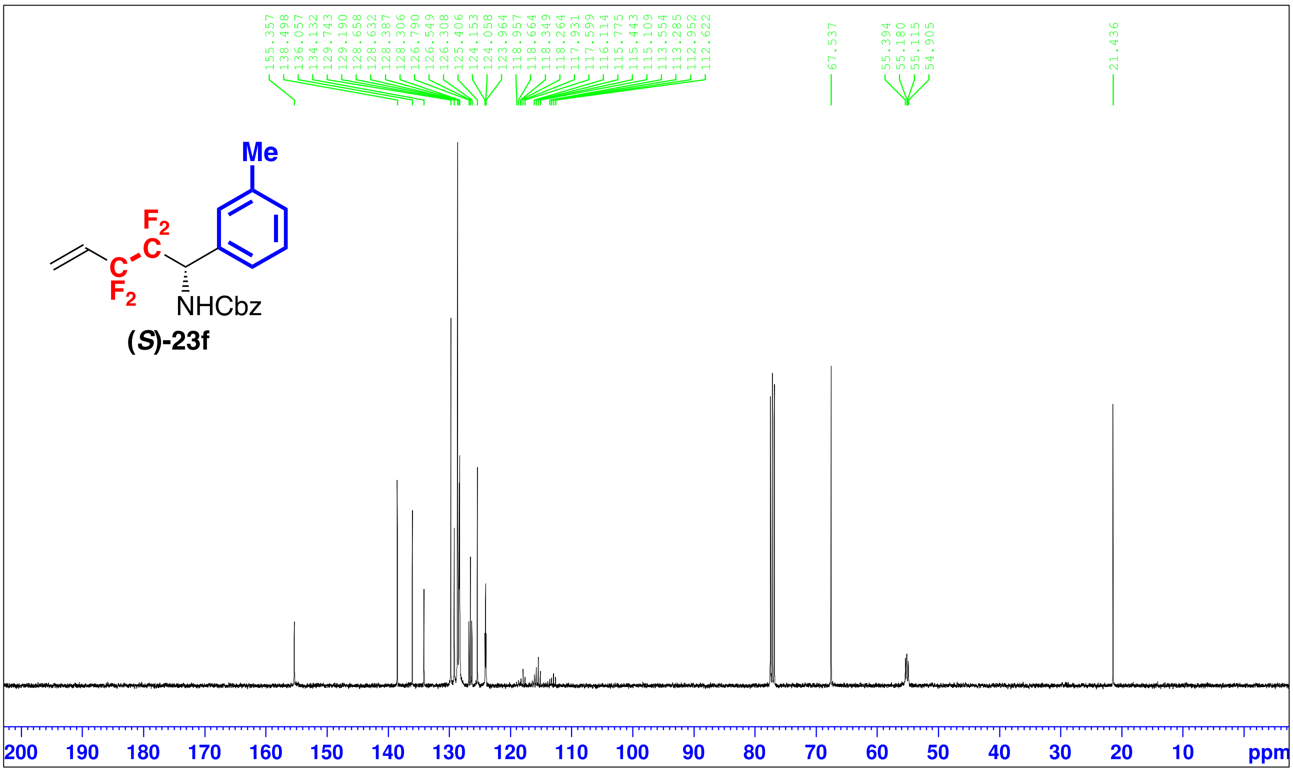

$^{19}$F NMR Spectrum of (*S*)-benzyl *N*-(2,2,3,3-tetrafluoro-1-(3-methylphenylpent-4-en-1-yl)carbamate ((*S*)-**23f**)

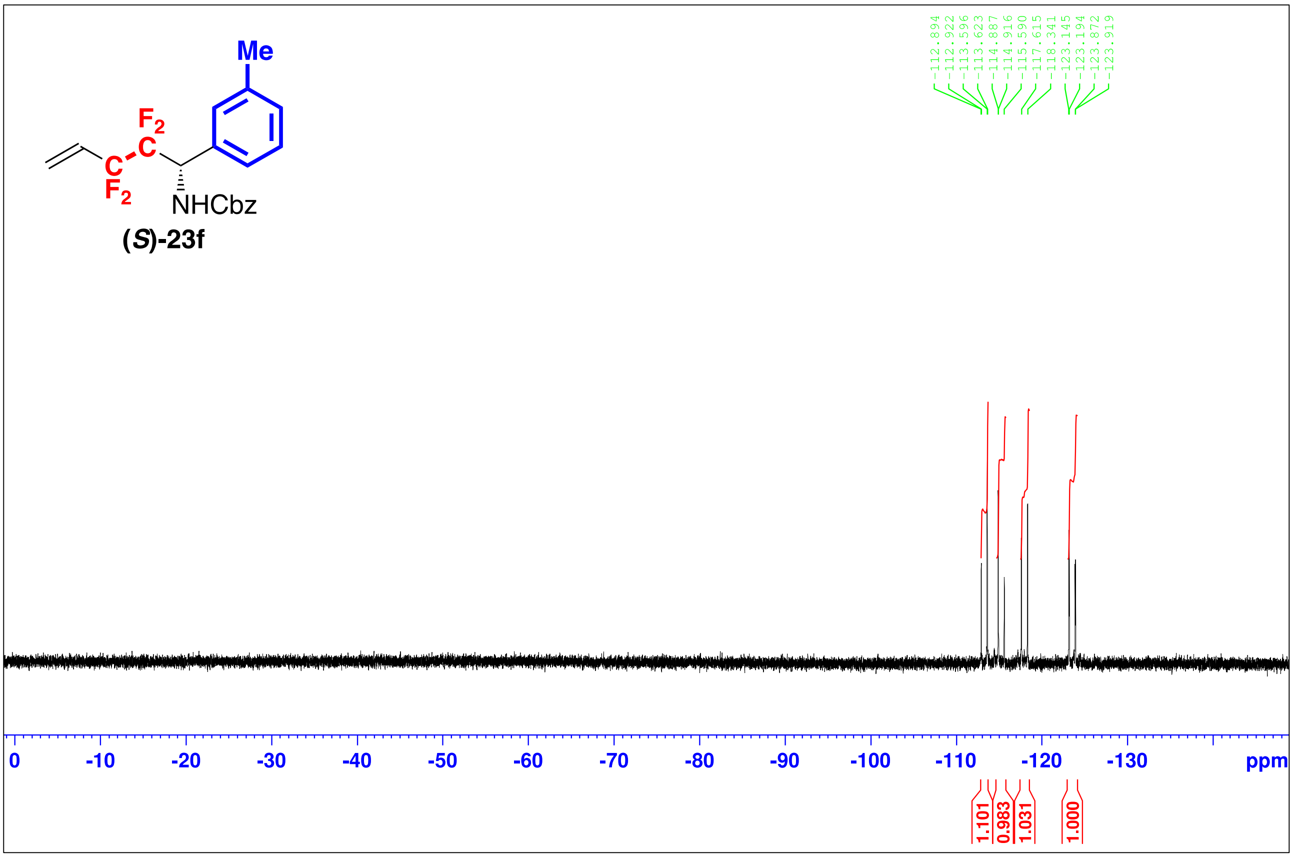


Chromatograph in HPLC for (*S*)-**23f**

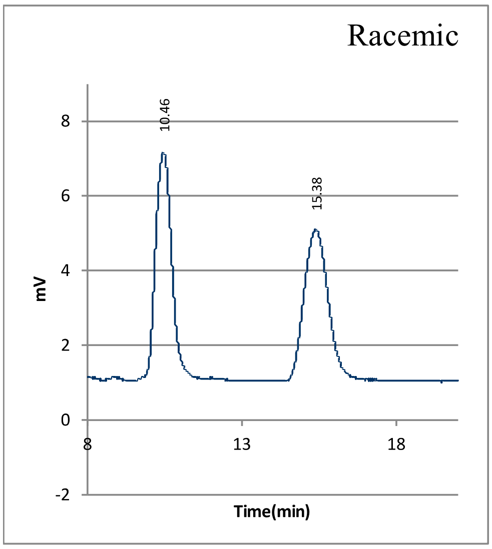


| No. | Rt | Area(%) |
|---|---|---|
| 1 | 10.46 | 50.086 |
| 2 | 15.38 | 49.914 |
| | | 100 |

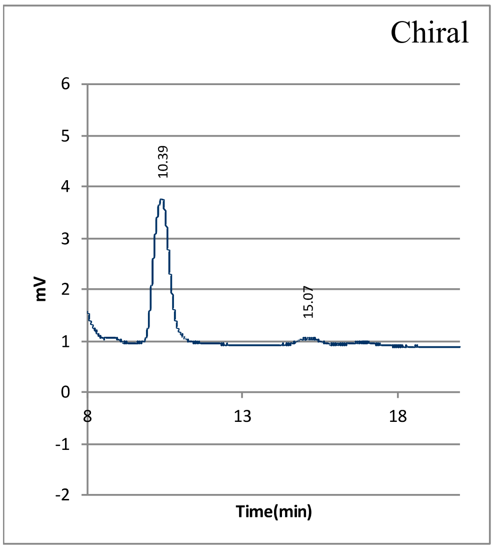


| No. | Rt | Area(%) |
|---|---|---|
| 1 | 10.39 | 95.504 |
| 2 | 15.07 | 4.496 |
| | | 100 |

$^{1}$H NMR Spectrum of (*S*)-benzyl *N*-(2,2,3,3-tetrafluoro-1-(2-methylphenylpent-4-en-1-yl)carbamate ((*S*)-**23g**)

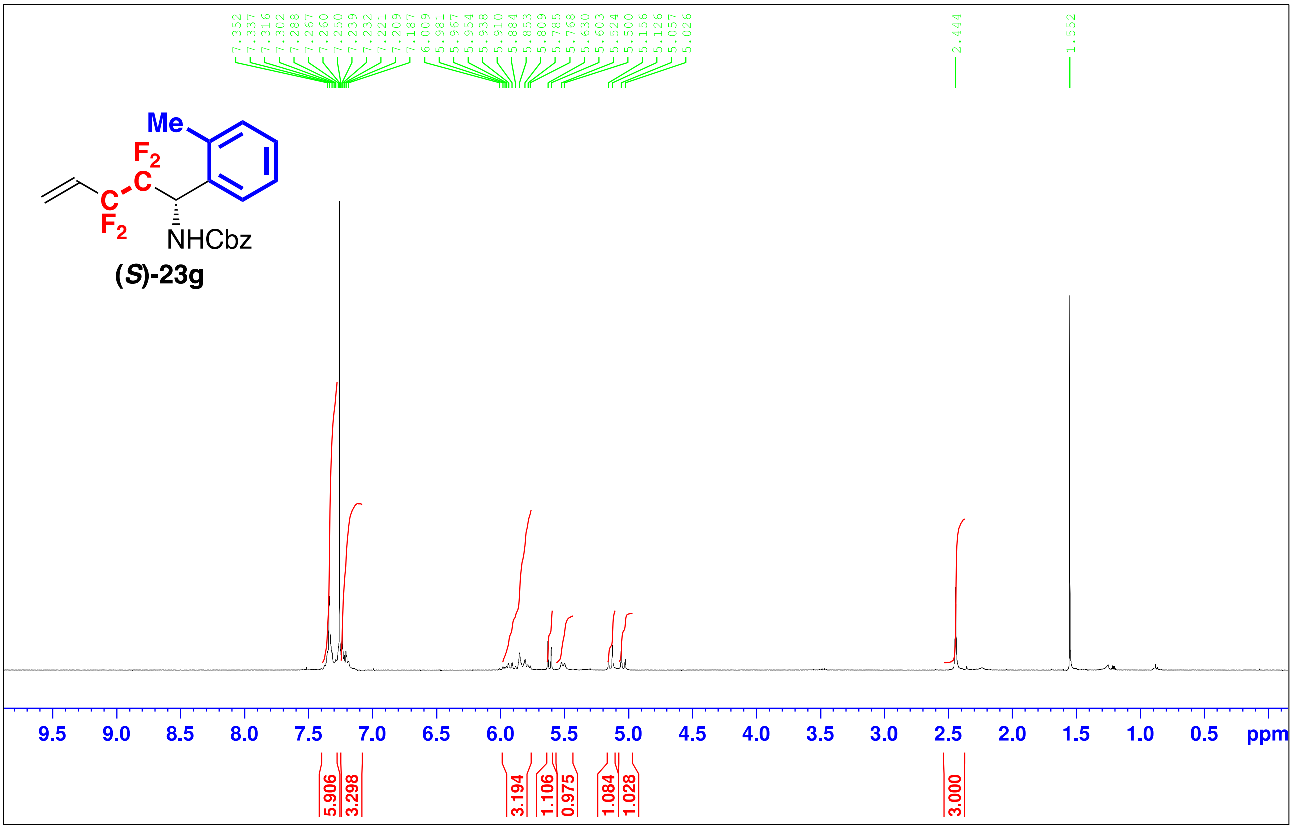

$^{13}$C NMR Spectrum of (*S*)-benzyl *N*-(2,2,3,3-tetrafluoro-1-(2-methylphenylpent-4-en-1-yl)carbamate ((*S*)-**23g**)

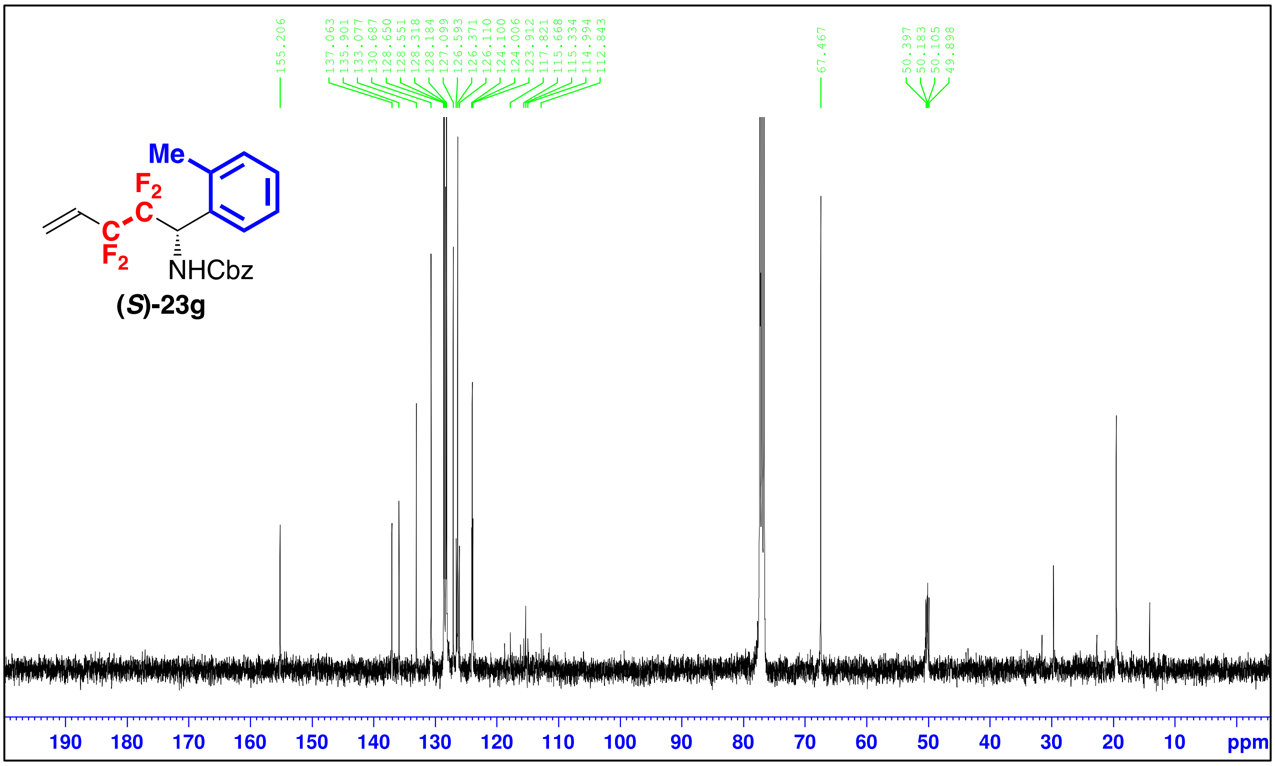

$^{19}$F NMR Spectrum of (*S*)-benzyl *N*-(2,2,3,3-tetrafluoro-1-(2-methylphenylpent-4-en-1-yl)carbamate ((*S*)-**23g**)

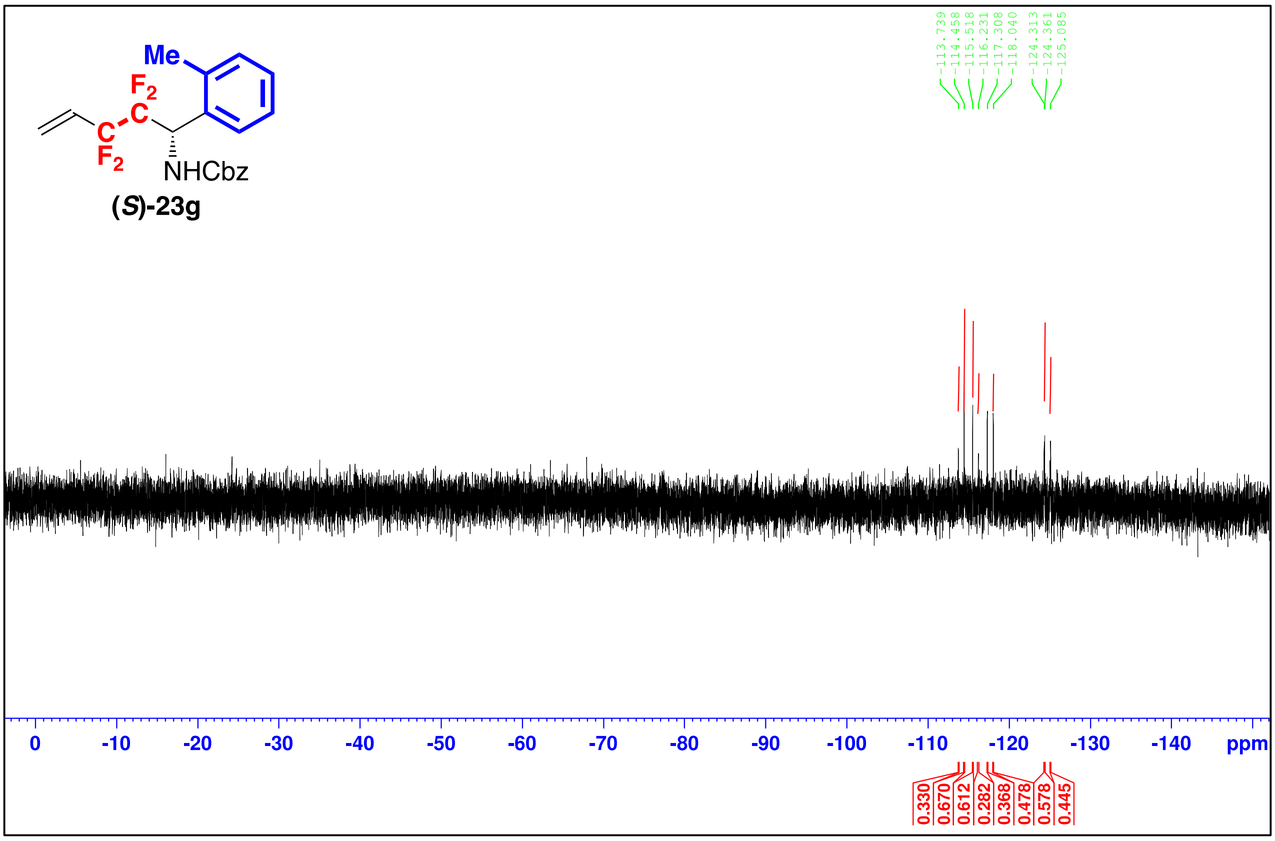


Chromatograph in HPLC for (*S*)-**23g**

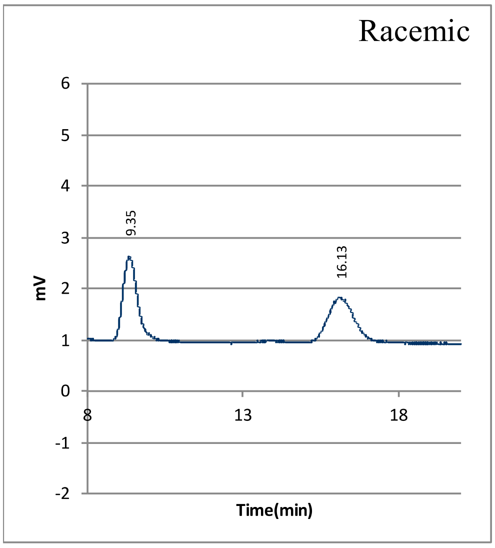


| No. | Rt | Area(%) |
|---|---|---|
| 1 | 9.35 | 49.913 |
| 2 | 16.13 | 50.087 |
| | | 100 |

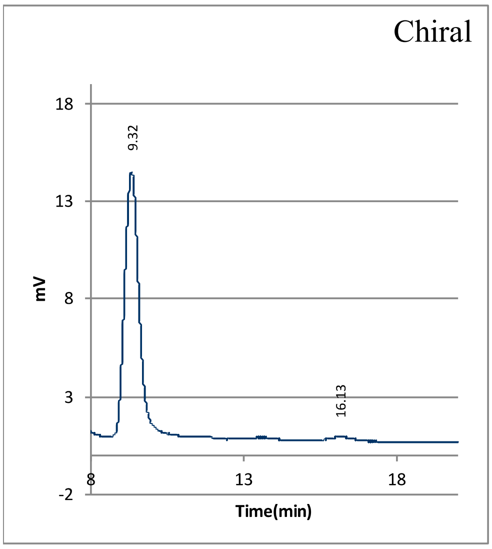


| No. | Rt | Area(%) |
|---|---|---|
| 1 | 9.32 | 97.056 |
| 2 | 16.13 | 2.944 |
| | | 100 |
